# Identification of Suppressors of *mbk-2/DYRK* by Whole-Genome Sequencing

**DOI:** 10.1534/g3.113.009126

**Published:** 2013-12-17

**Authors:** Yuemeng Wang, Jennifer T. Wang, Dominique Rasoloson, Michael L. Stitzel, Kevin F. O’ Connell, Harold E. Smith, Geraldine Seydoux

**Affiliations:** * Department of Molecular Biology and Genetics, Johns Hopkins University School of Medicine, Baltimore, Maryland 21205; † National Institute of Diabetes and Digestive and Kidney Diseases, National Institutes of Health, Bethesda, Maryland 20892

**Keywords:** whole-genome sequencing, single nucleotide polymorphism mapping, suppressors, DYRK kinase, MBK-2, *C. elegans*

## Abstract

Screening for suppressor mutations is a powerful method to isolate genes that function in a common pathway or process. Because suppressor mutations often do not have phenotypes on their own, cloning of suppressor loci can be challenging. A method combining whole-genome sequencing (WGS) and single nucleotide polymorphism (SNP) mapping (WGS/SNP mapping) was developed to identify mutations with visible phenotypes in *C. elegans*. We show here that WGS/SNP mapping is an efficient method to map suppressor mutations without the need for previous phenotypic characterization. Using RNA-mediated interference to test candidate loci identified by WGS/SNP mapping, we identified 10 extragenic and six intragenic suppressors of *mbk-2*, a DYRK family kinase required for the transition from oocyte to zygote. Remarkably, seven suppressors are mutations in cell-cycle regulators that extend the timing of the oocyte-to-zygote transition.

Whole-genome sequencing (WGS) has become a cost-effective method for the identification of mutations (Minevich *et al.* 2012). A protocol was recently developed for *C. elegans* that combines WGS with single nucleotide polymorphism (SNP) mapping to simultaneously map and sequence mutants generated by ethylmethanesulfonate (EMS) mutagenesis (Doitsidou *et al.* 2010). The mutant of interest is backcrossed against a polymorphic strain to generate ∼20–50 recombinants that are subjected to WGS in a single pool. The genomic region linked to the mutation is identified by its lower frequency of polymorphic SNPs compared to the rest of the genome. The low SNP region is then scanned for new EMS-induced mutations. This approach has been used to identify the causative molecular lesions for mutants with visible phenotypes (Doitsidou *et al.* 2010; Labed *et al.* 2012). We sought to investigate whether this approach could also be used to identify genetic suppressors, *i.e.*, mutations with no known phenotype other than the ability to suppress the phenotype of another mutation. Screening for genetic suppressors is a powerful method to identify genes that function in a common pathway or process (Hodgkin 2005). Because suppressors can be dominant or recessive and do not always cause a phenotype when outcrossed away from the original mutation, the molecular lesions associated with suppressors can be difficult to identify by traditional methods. We sought to determine whether one-step WGS/SNP mapping could be used to systematically map suppressor mutations using a collection of 20 suppressors isolated against a temperature-sensitive allele in the kinase MBK-2.

MBK-2 is a dual-specificity tyrosine-regulated kinase that functions during the transition from the oocyte to the zygote (Robertson and Lin 2013). During this transition, the egg undergoes two meiotic divisions before switching to mitosis in preparation for the first cleavage (Figure 1A). MBK-2 phosphorylates several oocyte proteins whose activities and/or levels need to be modified before the first cleavage (Robertson and Lin 2013). One conserved target of MBK-2 is the microtubule severing protein and meiotic regulator MEI-1 (Srayko *et al.* 2000). MEI-1 is abundant in oocytes but must be degraded before mitosis to allow the formation of the large mitotic spindle. MBK-2 phosphorylates MEI-1 beginning in the anaphase of meiosis I, and this phosphorylation stimulates MEI-1 turnover. In zygotes lacking MBK-2, MEI-1 degradation is delayed and MEI-1 activity interferes with the formation of the mitotic spindle, causing the first cleavage to fail (Figure 1A). MBK-2 is also required for segregation of the P granules (Pellettieri *et al.* 2003; Quintin *et al.* 2003). P granules are RNA granules specific to the germline that segregate to the posterior of the zygote for inheritance by the germline blastomere P_1_ (Strome 2005). The MBK-2 target required for P granule segregation is not yet known.

**Figure 1:**
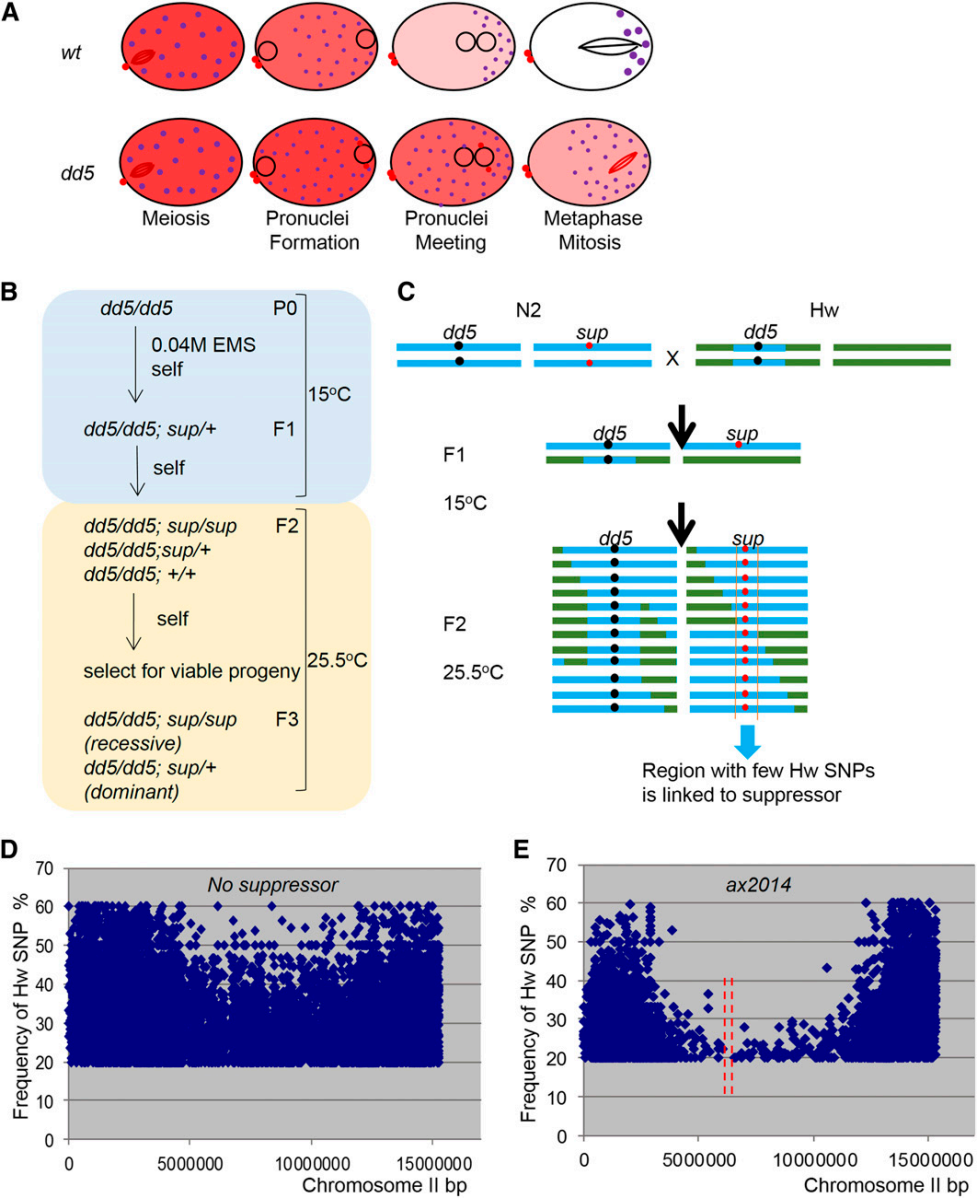
Isolation of suppressors of *mbk-2(dd5)*. (A) Schematic of the oocyte-to-embryo transition comparing the distribution of MEI-1 (red, high level; pink, intermediate level; white, absent) and P granules (purple) in wild-type and *mbk-2(dd5)* zygotes. Pronuclei (○) and spindle are also shown. (B) Scheme for the isolation of mutants that suppress the embryonic lethality of *mbk-2(dd5)*. (C) Scheme for the isolation of F2 Hawaiian/N2 recombinants carrying the suppressor mutations. Blue represents chromosomes region with N2 SNPs, and green represents chromosomes with Hawaiian SNPs. Note that for semi-dominant suppressors, a proportion of *mbk-2(dd5)*; *sup/Hw* recombinants will also be present in the sequenced pool. Graphs show the percentage of Hawaiian SNPs (Y axis) along linkage group (LG) II (X axis) in a population of F2 recombinants [derived as in (C)] carrying no suppressor (D) or the recessive suppressor *ax2014* (E). Each dot represents a unique Hawaiian SNP. Only SNPs with frequencies >20% are displayed.

We report the isolation and characterization by WGS/SNP mapping of 20 suppressors of the partial loss-of-function allele *mbk-2(dd5)* (Quintin *et al.* 2003). The suppressors include both recessive and semi-dominant alleles as well as intragenic mutations. Surprisingly, most of the extragenic suppressor loci encode cell-cycle regulators.

## Materials and Methods

### Nematode strains and maintenance

*C. elegans* strains were maintained as described by Brenner (1974). *mbk-2(dd5)* is an EMS-induced allele in the wild-type N2 (Bristol) background. Males carrying *mbk-2(dd5)* were backcrossed six times to CB4856 hermaphrodites (Hawaiian polymorphic strain) (Hodgkin and Doniach 1997) to generate *mbk-2(dd5)*^*Hw*^. Strains used in this study are listed in Supporting Information, Table S1.

### EMS suppressor screen

*mbk-2(dd5)* L4 hermaphrodites raised at 15° were soaked in 0.04 M EMS for 4 hr, washed, and allowed to recover for 2 hr at 15°. Approximately 5000 L4s (P0s) were picked to 54 enriched peptone plates (∼100 per plate), and every plate was processed separately to ensure the recovery of independent mutational events. In total, 500,000 gravid F1 progeny (10^6^ F1 genomes) were bleached to isolate approximately eight F2s per F1. The F2 progeny were grown at 25° and their progeny were screened for viable animals. Twenty stable lines (each derived from independent P0s) were backcrossed four times to unmutagenized *mbk-2(dd5)* and kept for further analysis.

#### Genetic analyses:

To distinguish between dominant and recessive suppressors, each suppressor line [*mbk-2(dd5)*; *sup*] was crossed with PD4790 *mbk-2(dd5)/mbk-2(dd5)*; *mIs12/mIs12* males [*mIs12* is a GFP transgene inserted on linkage group (LG) II]. GFP-positive cross-progeny [*mbk-2(dd5)/mbk-2(dd5)*; *sup/+*; *+/mIs12*] were allowed to lay eggs at 25°. Viable progeny indicated that the suppressor was dominant or semi-dominant. No viable progeny indicated that the suppressor was recessive. The *ax2002*, *ax2001*, and *ax2009* gave fewer progeny as heterozygotes compared to homozygotes (semi-dominant suppressors; data not shown).

To verify the linkage of potential intragenic suppressors, *ax2004*, *ax2005*, and *ax2006* were crossed to JH1279, which carries a GFP transgene inserted on LG IV, where *mbk-2* also resides. The *mbk-2(dd5 sup)*/GFP were allowed to self, and >20 GFP-negative progeny were tested for live progeny at 25°. All gave viable progeny, consistent with the suppressors being closely linked to *mbk-2(dd5)*.

#### Whole-genome sequencing and SNP mapping:

The *mbk-2(dd5)*; *sup* hermaphrodites were crossed to *mbk-2(dd5)^Hw^* males. F1 cross-progeny [*mbk-2(dd5)/mbk-2(dd5)^Hw^*; *sup/Hw*] were identified by PCR genotyping and 160–300 of their progeny (F2s) were allowed to self-fertilize *en masse* at the nonpermissive temperature for two more generations before harvesting for sequencing in a single pool. For recessive suppressors, only *mbk-2(dd5)*; *sup/sup* F2s (one-fourth of all F2s) will be propagated by this scheme because all other F2s will give rise only to dead progeny. For semi-dominant suppressors, a proportion of *mbk-2(dd5)*; *sup/Hw* will also be present in the sequenced pool.

For the semi-dominant suppressor *ax2001*, we preselected for homozygous F2 recombinants [*mbk-2(dd5)*; *sup/sup*] as follows: 120 F2 N2/Hw recombinants were plated singly and screened for >60% survivors among their progeny; 26 F2s were identified; and eight progeny (F3) per F2 were tested again for suppression. All 26 F2s gave 100% F3s with live progeny, confirming homozygosity at the suppressor locus. These 26 F2 recombinants lines were grown for two more generations before harvesting for sequencing in a single pool.

Libraries were prepared from genomic DNA according to the manufacturer’s protocol (Illumina, San Diego, CA). Briefly, DNA was sheared by sonication, end-repaired, A-tailed, adapter-ligated, size-fractionated by gel electrophoresis, and PCR-amplified. Sequence data were obtained using the HiSequation 2000 instrument (Illumina). A minimum of 36 million 50-nucleotide high-quality reads, equal to 18-fold coverage, were obtained from each library. Mutations were identified by a custom pipeline of BFAST for alignment (Homer *et al.* 2009), SAMtools for variant calling (Li *et al.* 2009), and ANNOVAR for annotation (Wang *et al.* 2010) with *C. elegans* genome version WS220 as the reference. Hawaiian SNP density was plotted against chromosome position and inspected visually. The introgressed mapping strain *mbk-2(dd5)^Hw^* contained a gap on LG I (1.5–12.8 Mbp) and a gap on LG IV (0–15.8 Mbp) flanking *mbk-2(dd5)*. Candidate intervals were defined by the absence (for recessive suppressors) or reduction (for dominant suppressors) of Hawaiian SNPs. Non-Hawaiian SNPs common to multiple strains were defined as background variants and removed. Candidate suppressor mutations were identified by unique variants (>85% for recessive suppressors; >67% for semi-dominant suppressors) within the relevant candidate interval.

#### RNAi experiments:

RNAi experiments were performed using feeding clones from the OpenBiosystem library (B0523.3, C03H5.4, C40H1.2, F25B5.7, F37A4.8, F46F5.11, F54C8.3, T05H10.5, T24H7.5, W08F4.8, Y48G1A.5, ZK1307.8, ZK177.6) or from the Arhinger library (C14B1.3, C14B9.4, F13H8.7, F23F12.3, F33G12.6, F37D6.1, R10F2.6, W10C6.1, ZC84.6), except for the *such-1* RNAi clone, which was made by amplifying a 0.9-kb *such-1* cDNA fragment using primers CGTGAGCAACTGTGATGCTC and TTAAATAAACATCCACGTGCATGG. This fragment was cloned using Gateway technology and pDEST-L4440 (Invitrogen). An empty pDEST-L4440 was used as a negative control.

RNAi was introduced into worms by feeding (Timmons and Fire 1998). The feeding vectors were transformed into *E. coli* HT115 strain, grown in liquid LB plus ampicillin (100 μg/ml) at 37° for 4 hr, induced with IPTG (5 mM) for 30 min, and plated on nematode nutritional growth media (NNGM) plus ampicillin (100 μg/ml) plus IPTG (1 mM). The bacteria were grown at room temperature for 1 d before worms were transferred onto the plates. Worms were fed starting in the L2 stage for >48 hr (“strong RNAi”), in the L4 stage for 24–30 hr (“medium RNAi”), or for 12–16 hr for (“weak RNAi”) before scoring the viability of embryos laid for a period of 4 hr. All three conditions were tested for the suppressors shown in Table 1. Results shown in Table 1 were obtained under strong RNAi conditions unless indicated otherwise.

**Table 1:** Candidate genes for the extragenic suppressors

Dominant/Recessive	Suppressor Allele	Mapping Interval*^a^*	Candidate Genes	Mutation Frequency*^b^*	RNAi on *mbk-2(dd5)**^c^* % Hatch at 25°
			blank		0% (n > 500)
Recessive	*ax2011*	I 2.0–11.0 M	*mus-101*	>90%	27% (n = 620)*^d^*
	*ax2013*	I 0–12.8 M	*xpo-2*	100%	23% (n = 600)*^e^*
			*Y65B4A.2*	94.12%	ND
	*ax2012*	II 4.6–11.9 M	*F33G12.6*	86.67%	0% (n = 520)
			*ZK1307.8*	93.75%	0% (n = 550)
			*ZK1307.8*	100%	0% (n = 550)
			*mat-2*	100%	15% (n = 570)*^e^*
	*ax2014*	II 5.4–7.0 M	*fzy-1*	>90%	30% (n = 780)*^e^*
	*ax2003*	III 7.8–10.1 M	*emb-30*	>90%	6% (n = 580)*^e^*
			*C40H1.2*	>90%	0% (n = 610)
	*ax2010*	III 11.4–12.5 M	*such-1*	>90%	25% (n = 610)*^e^*
	*ax2008*	III 8.0–9.8 M	*pgl-2*	>90%	0% (n = 550)
			*plk-1*	>90%	3% (n = 790)*^e^*
Semi-dominant	*ax2002*	III 2.9–12.0 M	*R10F2.6*	82.61%	0% (n = 520)
			*C14B1.3*	80.00%	0% (n = 510)
			*psf-1*	100%	0% (n = 550)
			*psf-1*	93.33%,	0% (n = 550)
			*F23F12.3*	77.78%	0% (n = 530)
			*isw-1*	85.71%	0% (n = 580)
			*plk-1*	78.05%	3% (n = 790)*^e^*
			*ZC84.6*	81.63%	0% (n = 510)
			*ZC84.6*	91.18%	0% (n = 510)
	*ax2001*	II 0–1.0 M	*C03H5.4*	87.50%	0% (n = 500)
			*cdc-37*	78.57%	0% (n = 520)
			*F46F5.11*	84.21%	0% (n = 500)
	*ax2009*	II 4.9–11.6 M	*tat-4*	95.00%	0% (n = 550)
			*upb-1*	77.78%	0% (n = 600)
			*ufd-2*	83.33%	0% (n = 500)

a Interval of low Hawaiian SNP frequency in F2 recombinants as determined by WGS.

b Frequency of the novel SNP in the gene indicated among F2 recombinants as determined by WGS. For some genes, two SNPs were detected.

c Percent of hatched (viable) progeny derived from *mbk-2(dd5)* hermaphrodites fed with RNAi bacteria against the gene listed in fourth column. n is the number of embryos scored.

d Medium RNAi: L4 hermaphrodites were fed with RNAi bacteria for 30 hr before scoring their progeny.

e Weak RNAi: L4 hermaphrodites were fed with RNAi bacteria for 16 hr before scoring their progeny.

**Figure 2:**
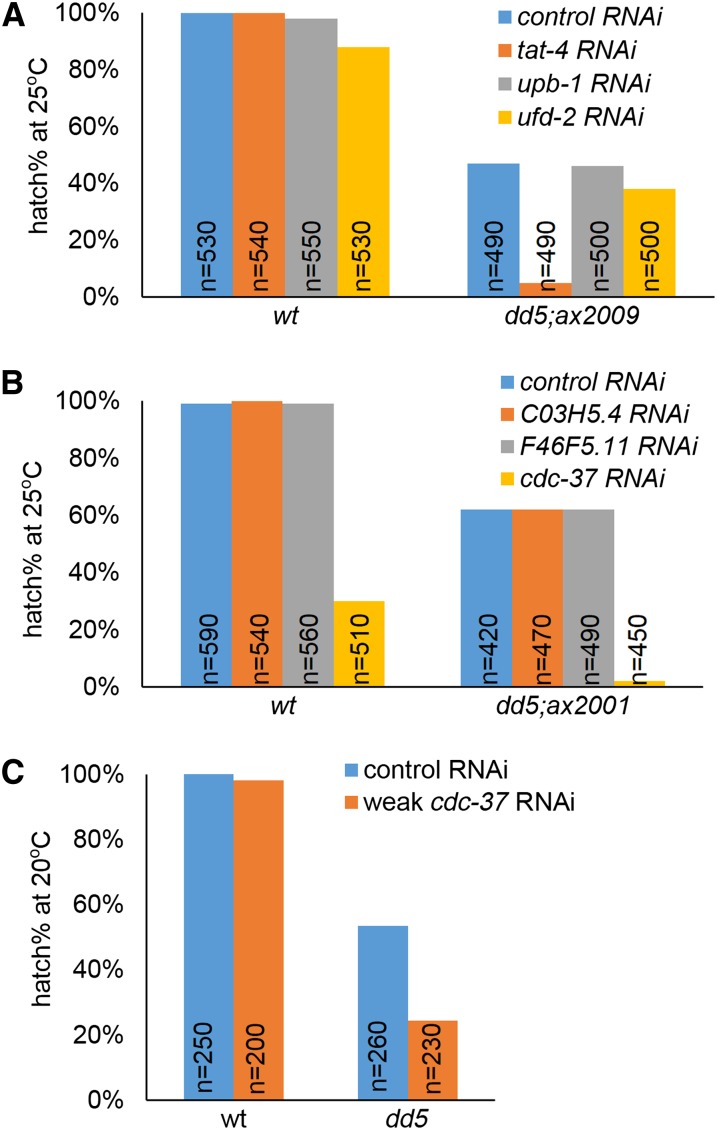
Identification of the suppressor loci for the dominant suppressors *ax2009* and *ax2001*. (A–C) Percent of hatched embryos among the progeny of hermaphrodites of the genotypes shown and fed dsRNA against the genes shown. (A) *tat-4(RNAi)* reverses the suppression of *ax2009*. *cdc-37(RNAi)* reverses the suppression of *ax2001* (B) and enhances the embryonic lethality of *mbk-2(dd5)* at the semi-permissive temperature of 20° (C).

Mild *cdc-37(RNAi)* as shown in Figure 2 was performed by feeding mothers for 16 hr and scoring embryos laid within that period.

#### Microscopy:

GFP::MEI-1 hermaphrodites were grown at 25° until they were young adults. Single-plane epifluorescence images of one-cell embryos in mitosis were taken using a 40× lens on a Zeiss Axioplan 2 equipped with Ludl shutters and a mercury lamp (exposure time, 100 ms). Slidebook 5.0 was used to normalize all images.

To determine the length of the oocyte-to-embryo transition, young adult hermaphrodites were anesthetized in 300 μM levamisole (Sigma) in M9 buffer for 30 min, placed on 3% agarose pad, and their eggs were examined by light microscopy (DIC optics) using a 63× 1.4NA plan apochromat lens to measure the time from ovulation (entry into spermatheca) to the appearance of pronuclei (meiotic divisions). Embryos dissected out of mothers were examined every minute to determine the time between the onset of pronuclear migration and the last time point before the pronuclei were no longer visible (pronuclear breakdown).

#### Immunostaining for P granules:

Adult hermaphrodites grown at 26° were processed for immunofluorescence as described by Gallo *et al.* (2010) using the anti-P granule antibody K76 (1:10; DSHB) and Cy-conjugated goat-anti-mouse IgM (1:100; Jackson Immunoresearch). DAPI was added in the mounting medium to stain DNA. Images were taken using a Zeiss Axio Imager fitted with a Yokogawa spinning-disc confocal scanner (63× 1.4NA plan apochromat lens). Z-stack images (0.27-μm intervals spanning entire embryo) were acquired and projected to a single plane under maximum projection. Slidebook 5.0 software was used to normalize all images.

#### In vitro kinase assay:

MBK-2 kinase assays were performed with partially purified MBP::MBK-2 and MBP::MEI-1 as described by Stitzel *et al.* (2006) and by Cheng *et al.* (2009). Point mutations were introduced using QuickChange site-directed mutagenesis kit (Invitrogen); 0.1 μM MBP::MBK-2(wt) and 0.3 μM MBP::MBK-2(KD), (dd5), (dd5ax2004), (dd5ax2005), and (dd5ax2006) were incubated with 0.6 μM MBP::MEI-1, 200 μM cold ATP and 300 μCi/μmol γ-^32^P-ATP (Amersham Pharmacia) at 30° for 2 hr. Reactions were stopped using LDS (Invitrogen) and applied to 7% SDS-PAGE (Invitrogen). Incorporated ^32^P signal was seen at the 99-kD position and was detected using phosphorimager (Amersham Pharmacia). In parallel, the same amounts of MBP::MBK-2 fusions were loaded on a second 7% SDS-PAGE and visualized by SimplyBlue SafeStain (Invitrogen). ^32^P signal intensity and kinase amount were quantified using ImageJ software to estimate relative kinase activity.

## Results

### Isolation, mapping, and sequencing of suppressors

*mbk-2(dd5)* is a missense mutation in a conserved residue in the kinase domain of MBK-2 (Quintin *et al.* 2003). When raised at 15°, *mbk-2(dd5)* hermaphrodites lay embryos that are all viable (100% hatching). In contrast, when raised at 25.5°, *mbk-2(dd5)* hermaphrodites lay embryos that do not survive embryogenesis (0% hatching) (Quintin *et al.* 2003). To identify suppressors of *mbk-2(dd5)*, we treated L4 *mbk-2(dd5)* hermaphrodites with EMS and collected rare survivors among F3 progeny grown at 25.5° (see *Materials and Methods*) (Figure 1B). Twenty independent suppressor lines were recovered from 10^6^ mutagenized genomes. Each suppressor line was backcrossed against the original unmutagenized *mbk-2(dd5)* line to reduce background mutations and to determine whether the suppressor mutation was dominant or recessive (see *Materials and Methods*). In total, we recovered 11 dominant suppressors and nine recessive suppressors. RNAi knock-down of MBK-2 caused 100% lethality in the suppressor lines, as it does in wild-type, indicating that none of the suppressors acts by bypassing the requirement for *mbk-2* activity (data not shown).

We mapped and sequenced the suppressors simultaneously using the WGS/SNP mapping method (Doitsidou *et al.* 2010). To simplify the identification of recombinants containing the suppressor alleles, we first introgressed *mbk-2(dd5)* into the polymorphic Hawaiian strain to create *mbk-2(dd5)^Hw^* . This strategy introduced Hawaiian SNPs throughout the genome, with the exception of two regions. One region flanks the *mbk-2* locus on LG IV. The second spans a large region on LG I (1.5–12.8 Mb) (Figure S1C), which contains a known locus of genetic incompatibility between the Hawaiian strain and the *mbk-2(dd5)* N2 (Bristol) background (Seidel *et al.* 2008). These gaps in Hawaiian SNP distribution in the introgressed line precluded high-resolution mapping on LG I and LG IV. However, we were still able to detect linkage to LG I and LG IV using Hawaiian SNPs on the ends of these chromosomes.

We crossed each suppressor line to *mbk-2(dd5)^Hw^* to create F1 hybrids [*mbk-2(dd5)*; *mbk-(dd5)^Hw^*; *sup/Hw*]; 160–300 hermaphrodites in the F2 generation [*mbk-2(dd5)*; *sup/Hw*, *sup/sup* or *Hw/Hw*] were grown *en masse* for two more generations at 25.5° to select for the suppressor mutation (Figure 1C). For three suppressor lines, no survivors were recovered; these suppressors were not pursued further. For each of the remaining 17 suppressor lines, survivors in the F4 generation were harvested for whole-genome DNA sequencing (one pool for each suppressor line; see *Materials and Methods*).

Mapping intervals were identified by a reduction in the allelic frequency of Hawaiian SNPs (low SNP frequency region). For one suppressor, the only low SNP frequency regions identified by sequencing were those regions also present in *mbk-2(dd5)^Hw^*, and this suppressor was not pursued further. For each of the 16 remaining suppressors, we detected a single new (unique) low SNP frequency region on LG I, LG II, or LG III (10 extragenic suppressors) (Table 1), or LG IV where *mbk-2* also resides (six linked suppressors). All six linked suppressors contained a new missense mutation in *mbk-2* (in addition to *dd5*) (Table 2). Remarkably, three carried the same base pair change; of these, only one (*ax2004*) was kept for further studies. *ax2004* is the strongest suppressor in our collection (Table 2). We subsequently confirmed linkage of *ax2004*, *ax2005*, and *ax2006* to *mbk-2* using a standard genetic test (see *Materials and Methods*).

**Table 2:** Summary of suppressors

Gene	Description*^a^*	Allele	Mutation	Conservation*^b^*	Domain	% Hatch*^c^*
*mbk-2*	Intragenic	*ax2004*	R433C	Identical	Kinase	97% (n = 290)
*mbk-2*	Intragenic	*ax2005*	L283F	No	Kinase	56% (n = 300)
*mbk-2*	Intragenic	*ax2006*	L353F	Identical	Kinase	47% (n = 340)
*mbk-2*	Intragenic	*ax2007*	V311I	Identical	Kinase	39% (n = 240)
*cdc-37*	Hsp90 co-chaperone	*ax2001*	L221F	Identical	Hsp90 binding	68% (n = 250)
*tat-4*	Lipid flippase	*ax2009*	G544S	Weak	Intracellular loop	47% (n = 260)
*plk-1*	Polo kinase	*ax2008*	L74F	Strong	Kinase	48% (n = 130)
*plk-1*	Polo kinase	*ax2002*	V87M	Strong	Kinase	39% (n = 150)
*fzy-1*	APC regulator	*ax2014*	D434N	Strong	WD40	37% (n = 300)
*such-1*	APC subunit	*ax2010*	L17F	No	—	39% (n = 230)
*emb-30*	APC subunit	*ax2003*	Splice site	—	—	35% (n = 260)
*mat-2*	APC subunit	*ax2012*	V1208M	Strong	—	23% (n = 260)
*mus-101*	DNA replication	*ax2011*	S273N	No	BRCT	49% (n = 150)
*xpo-2*	Mitotic importin	*ax2013*	S679L	Strong	Nuclear transport	41% (n = 280)

a Abbreviated description as obtained from Wormbase WS239.

b This column indicates whether the residue that is mutated in the suppressor is conserved in the *D. melanogaster* and human orthologs. Identical denotes an amino acid that is fully conserved in all three species. Strong denotes a position that scores >0.5 in the Gonnet PAM 250 matrix. Weak denotes a position that scores ≤0.5 in the Gonnet PAM 250 matrix. No denotes a position that is not conserved. See Figure S4 for full alignments.

c Percent of hatched (viable) progeny derived from *mbk-2(dd5); suppressor* hermaphrodites at 25°. n is the number of embryos scored.

For one extragenic suppressor (*ax2001)*, the linked region identified by WGS/SNP mapping spanned most of LG II (Figure S1). Because *ax2001* is semi-dominant, a significant proportion of *mbk-2(dd5)*; *ax2001/Hw* recombinants were present in the sequenced pool. To avoid this problem, we screened 120 F2 recombinants for homozygosity at the suppressor locus (see *Materials and Methods*). Twenty-six homozygous *mbk-2(dd5)*; *ax2001/ax2001* recombinants were grown for two generations and pooled for sequencing. This approach narrowed the mapping interval to a 1.0-Mb region on LG II (Figure S1).

Mapping intervals were scanned for missense, nonsense, or splice-site mutations that were absent in the parental *mbk-2(dd5)* strain and present at >85% allele frequency (recessive suppressors) or >67% allele frequency (semi-dominant suppressors) in the sequenced pool. These analyses yielded between one and seven candidate loci for each of the 10 extragenic suppressors (Table 1).

### Identification of the extragenic suppressor loci using RNAi

#### Recessive suppressors:

Because the WGS/SNP mapping approach identified more than one gene for seven of the 10 extragenic suppressors, we devised additional tests to identify the locus responsible for the suppression. We reasoned that, in cases in which the suppression is caused by a reduction or loss-of-function allele in the suppressor locus, knockdown of the suppressor locus by RNAi should also suppress the embryonic lethality of *mbk-2(dd5)* (phenocopy). We knocked-down each candidate by RNAi in *mbk-2(dd5)* hermaphrodites raised at 25° and scored as positive any RNAi treatment that gave viable progeny. We used three strengths of feeding RNAi (see *Materials and Methods*) to maximize the chances of finding positives (Fievet *et al.,* 2012). This approach identified one positive locus for each of the seven recessive extragenic suppressors (Table 1). Remarkably, all seven loci code for proteins implicated in cell-cycle regulation (Table 2). In all cases, the degree of suppression observed by RNAi was significantly weaker than that observed in the suppressor lines. Strong loss-of-function of several of these genes has been shown to cause cell-cycle arrest and embryonic lethality (Fraser *et al.* 2000; Golden *et al.* 2000; Davis *et al.* 2002; Kitagawa *et al.* 2002; Holway *et al.* 2005; Budirahardja and Gonczy 2008; Rivers *et al.* 2008; Stein *et al.* 2010). Consistent with these findings, we found that strong RNAi conditions for all of the cell-cycle genes caused nearly 100% lethality in wild-type animals (*Materials and Methods* and data not shown). No suppression of *mbk-2(dd5)* was observed under strong RNAi conditions. However, weaker RNAi conditions that caused less lethality in wild-type could suppress the embryonic lethality of *mbk-2(dd5)* (in a minority of embryos) (Table 1). These observations suggest that the suppressor alleles are partial loss-of-function alleles (hypomorphs) that retain enough activity to allow some embryos to survive embryogenesis.

#### Dominant suppressors:

Among the dominant suppressors, the RNAi approach described identified a positive locus (*plk-1*) only for *ax2002*. *plk-1* was also found as a candidate for the recessive suppressor *ax2008*. *ax2002* and *ax2008* both map to the kinase domain of PLK-1 but affect different conserved amino acids (Table 2), which may explain why one allele is recessive and the other is weakly semi-dominant.

For the two remaining dominant suppressors, RNAi in the *mbk-2(dd5)* background failed to identify any positive candidates (Table 1). We reasoned that these suppressor mutations could correspond to gain-of-function, rather than loss-of-function, alleles. If so, then RNAi of the suppressor loci would not be expected to suppress the embryonic lethality of *mbk-2(dd5)*. RNAi of the suppressor loci, however, should reverse the suppression in the *sup; mbk-2(dd5)* strain. Using this approach, we found that among the candidates for *ax2001* and *ax2009*, RNAi of *cdc-37* and *tat-4*, respectively, reversed the suppression (Figure 2, A and B).

To verify the specificity of the reversal of suppression, we also inactivated *cdc-37* and *tat-4* by RNAi in wild-type and in the other suppressor lines. In the case of *tat-4*, a nonessential gene (Lyssenko *et al.* 2008), *tat-4(RNAi)*, reduced the viability of *mbk-2(dd5); ax2009*, but not of wild-type or the other suppressor lines, confirming specificity (Figure 2A and Figure S2). Using the same test, we were not able to demonstrate specificity for *cdc-37* because, unlike *tat-4*, *cdc-37* is an essential gene (Beers and Kemphues 2006). RNAi of *cdc-37* also reduced the viability of wild-type at 25° (Figure 2B) and of other suppressor lines (data not shown).

We reasoned that if *ax2001* was indeed a gain-of-function allele of *cdc-37* that suppresses the embryonic lethality of *mbk-2(dd5)*, loss of *cdc-37* might have the opposite effect and enhance the embryonic lethality of *mbk-2(dd5)* at the semi-permissive temperature. We first identified *cdc-37(RNAi)* conditions that had no effect on the viability of wild-type embryos at 20° (see *Materials and Methods*). We then tested these same RNAi conditions on *mbk-2(dd5)* raised at 20°. We found that *cdc-37(RNAi*) significantly reduced the viability of *mbk-2(dd5)*, confirming that *cdc-37* and *mbk-2* interact genetically (Figure 2C).

Unlike the other suppressor loci described, *tat-4* and *cdc-37* are not predicted to be cell-cycle regulators. *tat-4* codes for one of six predicted aminophospholipid translocases (flippase) in the *C. elegans* genome (Lyssenko *et al.* 2008). *cdc-37* is the sole *C. elegans* Cdc37 homolog, a co-chaperone for Hsp90 that helps stabilize kinase domains (Beers and Kemphues 2006).

### Phenotypic characterization of the suppressors

**Figure 3:**
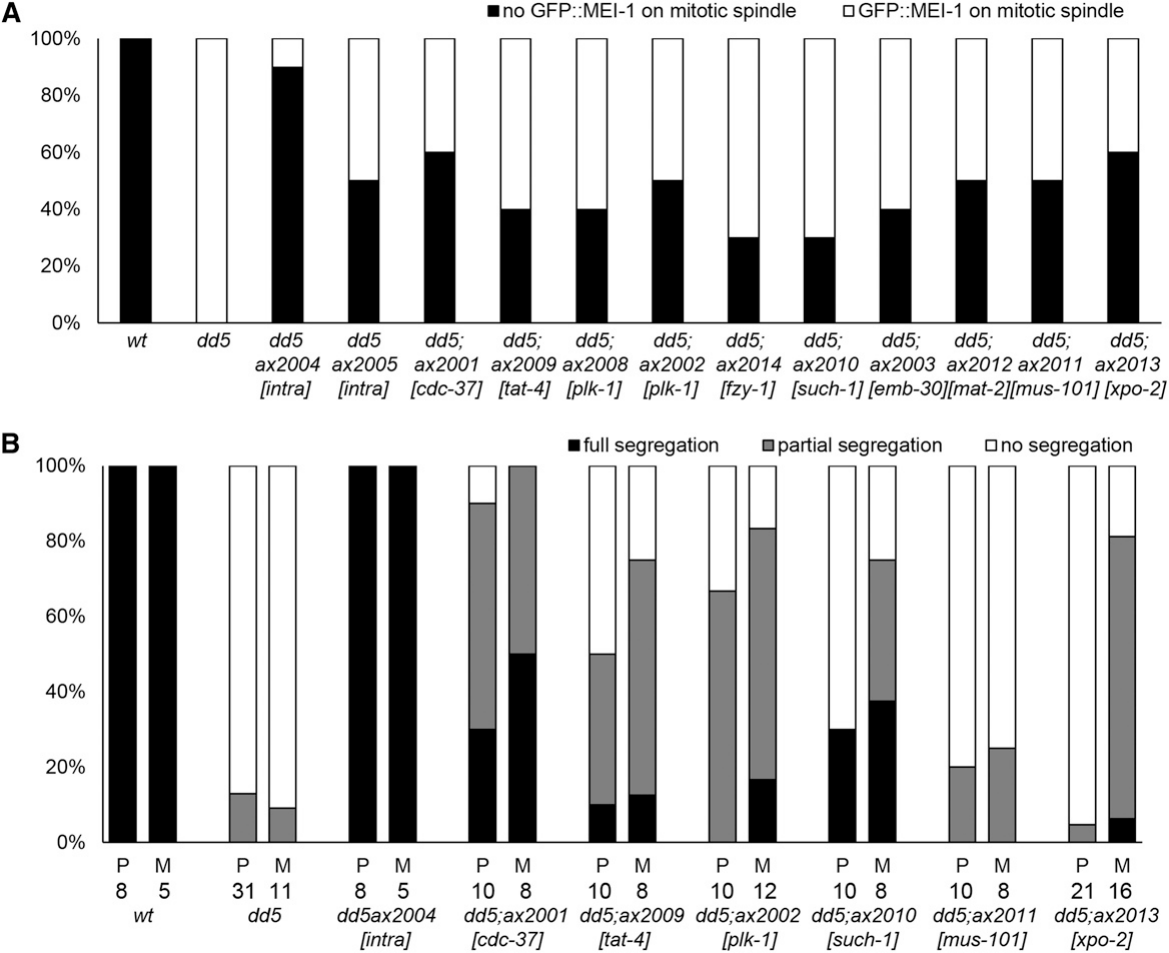
Rescue of the MEI-1 and P granule defects by the suppressors. (A) The genotypes indicated were scored for GFP::MEI-1 on the mitotic spindle. Black indicates no GFP::MEI-1 on spindle (as in wild-type) and white indicates GFP::MEI-1 on spindle [as in *mbk-2(dd5)*]. Ten zygotes scored for each genotype (see Figure S3 for representative images). The intragenic suppressor *ax2004* shows the most efficient rescue. (B) Zygotes at pronuclear meeting (P) or metaphase of the first mitosis (M) were scored for P granule asymmetry. Black indicates full segregation (all P granules in posterior half of the zygote as in wild-type), gray indicates partial segregation (some P granules remaining in anterior half of the zygote), and white indicates no segregation (P granules uniformly distributed throughout the cytoplasm. The numbers of zygotes scored are indicated below each bar (see Figure S3 for representative images). The intragenic suppressor *ax2004* was also the most efficient suppressor by this assay.

The suppressors rescue the embryonic lethality of *mbk-2(dd5)* with varying penetrance (Table 2). To determine whether the suppressors rescue *mbk-2(dd5)* phenotypes in zygotes, we first examined the suppressors for their ability to rescue MEI-1 degradation. We crossed a GFP::MEI-1 fusion (Stitzel *et al.* 2006) into *mbk-2(dd5)* and the suppressor lines and scored the percent of zygotes with GFP::MEI-1 on the mitotic spindle. GFP::MEI-1 was absent from the mitotic spindle in all wild-type zygotes and was present in all *mbk-2(dd5)* zygotes. We found that all suppressors at least partially suppressed the GFP::MEI-1 defect of *mbk-2(dd5)* zygotes (Figure 3A). The most efficient suppressor was the intragenic suppressor *ax2004*.

We also tested a subset of suppressors for their ability to suppress the P granule segregation defect of *mbk-2(dd5)* zygotes. We stained fixed zygotes with the anti-P granule antibody K76 (Strome and Wood 1983) (see *Materials and Methods*). P granules become restricted to the posterior half of the zygote by pronuclear meeting in wild-type, but not in *mbk-2(dd5)*, zygotes. By this assay, the degree of suppression was highly variable among the suppressors tested, ranging from complete (intragenic suppressor *ax2004*) to very weak (Figure 3B). For the suppressors *mus-101* and *xpo-2*, no rescue was observed when zygotes were scored at pronuclear meeting, and only weak, partial rescue was detected in zygotes scored at mitosis.

### Cell-cycle suppressors extend the timing of the oocyte-to-zygote transition

**Figure 4:**
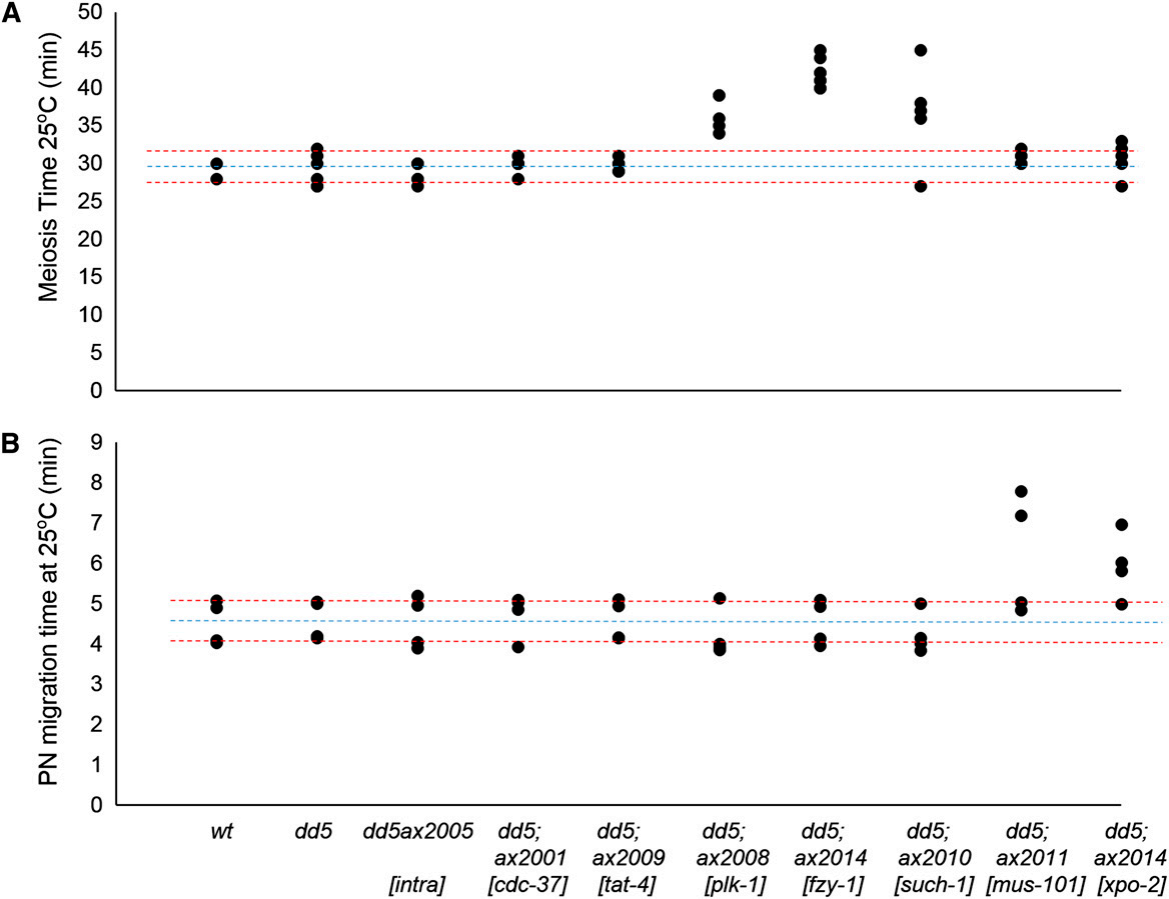
Cell-cycle suppressors extend the timing of the oocyte-to-zygote transition. Time spent from ovulation to pronuclear formation (A) and from the onset of pronuclear migration to pronuclear breakdown (B) for zygotes of the indicated genotypes (see *Materials and Methods*). Each dot represents a single zygote. Blue lines show the average time and red lines show the 95% confidence interval for wild-type zygotes.

Because several of the suppressor loci code for cell-cycle regulators, we hypothesized that some could suppress *mbk-2(dd5)* indirectly by extending the timing of the oocyte-to-zygote transition when MBK-2 phosphorylates its substrates. To investigate this possibility, we filmed live zygotes to measure time spent from ovulation to pronuclear formation (meiotic divisions) and from the beginning of pronuclear migration to pronuclear breakdown (S phase/mitotic entry). We found that *mbk-2(dd5)*, *mbk-2(dd5ax2005)*, *mbk-2(dd5); cdc-37(ax2001)*, and *mbk-2(dd5); tat-4(ax2009)* all had normal timing. In contrast, we found that all cell-cycle suppressors tested extended the timing of the transition (Figure 4). Suppressor lines with mutations in *plk-1*, the anaphase-promoting complex (APC) regulator *fzy-1*, and the APC subunit *such-1* had a longer first period, consistent with the known role for these genes in meiosis (Golden *et al.* 2000; Budirahardja and Gonczy 2008; Rivers *et al.* 2008). Suppressor lines with mutations in *mus-101* and *xpo-2* (http://www.wormbase.org version WS238) had a longer second period, consistent with the predicted role for these genes in DNA replication and mitotic entry, respectively (Holway *et al.* 2005).

We observed four *mbk-2(dd5)*; *mus-101(ax2011)* zygotes until cytokinesis. We found that the two zygotes with the shortest pronuclear migration times failed cytokinesis, whereas the two with the longest pronuclear migration times completed cytokinesis (Figure 4 and data not shown). These observations suggest a correlation between cell-cycle delay and rescue of the *mbk-2(dd5)* phenotype.

### Characterization of the intragenic suppressors

**Figure 5:**
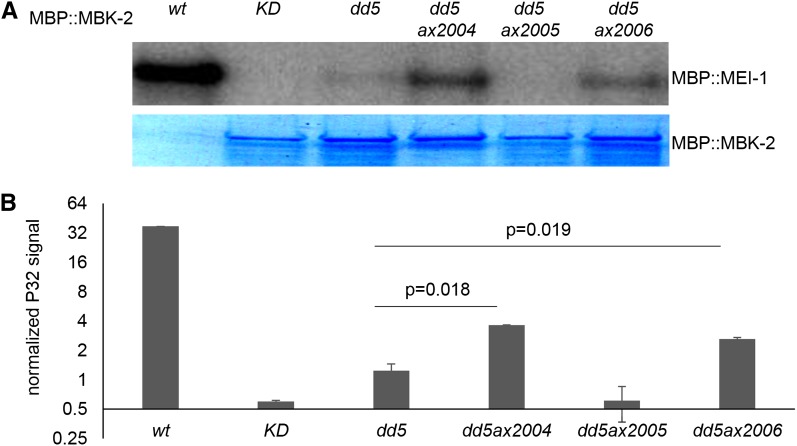
Rescue of kinase activity by intragenic suppressors *ax2004* and *ax2006*. (A) Representative example of a kinase assay using recombinant MBP-tagged MBK-2 expressed in *E. coli*. Upper panel shows ATP incorporation into the substrate MBP::MEI-1. Lower panel shows Coomassie blue–stained MBP::MBK-2 used in the assay to control for loading. KD is MBK-2(K196R) with a mutation in the ATP binding site. (B) Graph showing the relative activity (arbitrary units) of MBP::MBK-2 fusions obtained from two independent kinase assays [as in (A)]. Double-tail Student *t* test was applied to compare each suppressor to MBK-2(dd5).

The intragenic suppressors all map to the kinase domain (Table 2), where *dd5* also resides, raising the possibility that these suppressors might rescue kinase activity. To test this possibility, we performed *in vitro* kinase assays using recombinant MBK-2 and substrate MEI-1 (Stitzel *et al.* 2006). As expected, we found that recombinant MBK-2(*dd5*) has reduced kinase activity compared to recombinant MBK-2(WT). MBK-2(*dd5ax2005*) showed no detectable improvement over MBK-2(*dd5*), whereas MBK-2(*dd5ax2004*) and MBK-2(*dd5ax2006*) exhibited activity levels intermediate between that of MBK-2(*dd5)* and wild-type (Figure 5, A and B). Surprisingly, these results did not correlate well with the ability of each suppressor to rescue the embryonic lethality of *mbk-2(dd5) in vivo* (Table 2). For example, although *ax2005* showed no detectable rescue in the kinase assay, *ax2005* rescued the embryonic lethality of *mbk-2(dd5)* to a similar extent as *ax2006* (56% *vs.* 47%) (Table 2). These results raise the possibility that the intragenic suppressors use different mechanisms to restore MBK-2 function (see *Discussion*).

## Discussion

### WGS/SNP mapping is an efficient method to identify candidate suppressor mutations

The WGS/SNP mapping strategy was originally devised to identify mutations with a visible phenotype (Doitsidou *et al.* 2010). In this study, we show that this approach is also an efficient method to identify suppressors of a temperature-sensitive embryonic lethal mutation. The WGS/SNP mapping approach relies on deep sequencing of a combined pool of 20–50 recombinants containing the mutation of interest in the background of the polymorphic CB4856 Hawaiian strain. We greatly simplified this step by first backcrossing the embryonic lethal mutation *mbk-2(dd5)* into the Hawaiian strain [*mbk-2(dd5)^Hw^*]. In this way, we could select (rather than screen) for recombinants containing the suppressors, because these were the only survivors among the progeny of *mbk-2(dd5)/mbk-2(dd5)^Hw^; sup/Hw* hybrids raised at the nonpermissive temperature. This approach does not require previous phenotypic characterization of the suppressors.

For the seven recessive suppressors, WGS/SNP mapping was sufficient to define a single linked region containing only one to four candidates. The mapping resolution was lower for the dominant suppressors, because those alleles can rescue when heterozygous. Nevertheless, we were able to restrict two of the three semi-dominant suppressors to intervals containing manageable numbers of candidates (three or seven). This success may have been attributable to the fact that these suppressors produce more live progeny as homozygotes than as heterozygotes (semi-dominant), and we propagated the recombinants *en masse* at the nonpermissive temperature for two generations before sequencing. The continuous selection is likely to have enriched for hermaphrodites homozygous for the suppressor mutation. This approach, however, was not sufficient to map dominant suppressor *ax2001*. For that suppressor, we manually screened the progeny of F1 hybrids for homozygous *sup/sup* recombinants. Sequencing of 26 homozygous recombinants in a single pool reduced the size of the mapping interval from 12 Mb to 1 Mb.

We used RNAi to identify the suppressor locus among the candidates in each linked region. RNAi reduces gene activity and therefore can be used to phenocopy loss-of-function suppressors or to reverse the suppression of gain-of-function suppressors. We used three strengths of RNAi (strong, medium, and weak) to maximize our chances of finding conditions strong enough to reveal the genetic interaction but weak enough to maintain any essential functions of the suppressor loci. This approach was successful in identifying a single locus for each of the 10 mapped extragenic suppressors.

The intragenic suppressors could not be mapped precisely by WGS/SNP mapping because of their dominance and linkage to *mbk-2*. Nevertheless, we identified six candidate intragenic suppressors, representing four different mutations, based on their linkage to LG IV and the presence of a second mutation in *mbk-2* (in addition to *dd5)*. We subsequently confirmed linkage to *mbk-2* for *ax2004*, *ax2005*, and *ax2006*. Ultimately, to confirm the identification of each suppressor locus and to exclude possible contributions from closely linked mutations, it will be necessary to reintroduce each suppressor mutation in a clean unmutagenized background. The development of methods for CRISPR-mediated homologous recombination should soon make this final test possible (Chen *et al.* 2013; Chiu *et al.* 2013; Dickinson *et al.* 2013; Friedland *et al.* 2013; Katic and Grosshans 2013; Tzur *et al.* 2013; Waaijers *et al.* 2013).

We failed to map four suppressors out of 20. Three suppressors could not be recovered after crossing to the polymorphic Hawaiian strain. All three were among our weakest suppressors, and Hawaiian alleles may have interfered with the already weak suppression. One other suppressor (dominant) yielded no unique linked region by WGS/SNP mapping and had no mutations in *mbk-2* besides *dd5*. One possibility is that this suppressor lies in one of the gaps in Hawaiian SNPs present in the *mbk-2(dd5)^Hw^* introgressed strain. Despite backcrossing *mbk-2(dd5)* six times into CB4856, *mbk-2(dd5)^Hw^* still retained primarily non-Hawaiian SNPs on most of LG IV (0–15.8 Mb out of 17.5 Mb), where *mbk-2* resides, and on LG I (1.5–12.8 Mb out of 15 Mb), where the *peel-1-zeel-1* N2/Hw incompatibility locus resides (Seidel *et al.* 2008). Both of these gaps could be reduced or eliminated by judicious design of the backcrosses used to generate the introgressed strain. The N2/Hw incompatibility locus causes paternal effect/zygotic lethality for Hw/Hw embryos sired by sperm derived from N2/Hw heterozygotes (Seidel *et al.* 2011). Therefore, we recommend using Hw/Hw males in the backcrosses to permit introgression of chromosome I. The gap surrounding *mbk-2(dd5)* could be reduced by selecting for recombinants with LG IV Hawaiian SNPs after each backcross (Wicks *et al.* 2000) until the desired minimal interval flanked by homozygous Hawaiian SNPs is obtained. Finally, we recommend sequencing the introgressed strain to identify any remaining gaps before use.

### Intragenic suppressors map to different regions in the kinase domain

**Figure 6:**
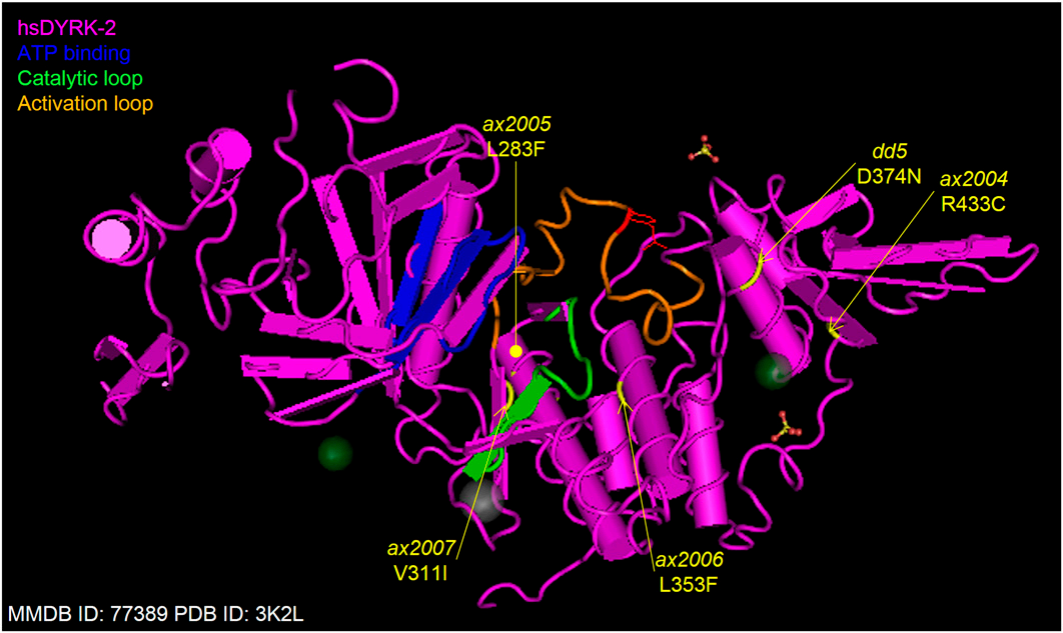
Position of the intragenic suppressors in MBK-2. *C. elegans* MBK-2 sequence was superimposed onto the structure of human DYRK2 (MMDB ID: 77389; PDB ID: 3K2L). The catalytic loop, ATP binding region, and activation loop are shown in different colors. The phosphorylated tyrosine in the activation loop is shown in red. Amino acids mutated in *dd5* and the four intragenic suppressors are highlighted in yellow. *ax2005* maps near the activation loop on the opposite side of the molecule and is not visible in this view.

We tested three of the intragenic suppressors in an *in vitro* kinase assay for rescue of the intrinsic kinase activity of MBK-2(dd5). Two suppressors partially rescued activity (*ax2004* and *ax2006*) and one did not (*ax2005*), suggesting that the intragenic suppressor mutations may use different mechanisms to suppress the *dd5* mutation. Superimposition of the MBK-2 amino acid sequence on the crystal structure of human DYRK2 (Soundararajan *et al.* 2013) (Figure 6) allowed us to predict the relative position of *dd5* and the suppressor mutations. We found that *dd5* and *ax2004* (the strongest suppressor *in vivo*) are located on neighboring alpha helices, raising the possibility that *ax2004* directly compensates for structural changes caused by *dd5*. *ax2006*, in contrast, maps to a different region closer to the catalytic pocket and *ax2005* maps to the back side of the catalytic pocket near the activation loop. Because *ax2005* does not improve kinase activity detectably *in vitro*, *ax2005* may modulate MBK-2 activity indirectly by modifying the binding of a regulator. Possible candidates are the pseudophosphatases EGG-4 and EGG-5, which recognize the phosphorylated form of the activation loop (Cheng *et al.* 2009). Alternatively, *ax2005* may not be the lesion responsible for the suppression and the suppressor mutation may reside elsewhere in a locus closely linked to *mbk-2*.

### Most extragenic suppressor loci code for cell-cycle regulators

Remarkably, we found that seven of the nine extragenic suppressors map to genes that code for cell-cycle regulators. Previous studies have shown that cyclin-dependent kinase 1 and the APC are required for MBK-2 activation at the onset of the meiotic divisions (Stitzel *et al.* 2007; Cheng *et al.* 2009). Therefore, it was surprising to find that loss-of-function mutations in cell-cycle regulators would suppress a partial loss in MBK-2 activity. Several lines of evidence suggest that this suppression is indirect. First, the suppressor loci code for a diverse set of cell-cycle regulators predicted to regulate different cell-cycle stages (metaphase-to-anaphase transition during meiosis, S phase, and mitotic entry). Second, all the cell-cycle suppressors extend the timing of the oocyte-to-zygote transition, which is the critical period when MBK-2 phosphorylates its substrates (Nishi and Lin 2005; Stitzel *et al.* 2007). Finally, the suppressors that function early in the transition (*plk-1* and APC) rescue the P granule segregation defect earlier and more efficiently than the suppressors that function later in the transition (*mus-101* and *xpo-2*). Together, these observations suggest that the suppressor mutations in the cell-cycle loci do not affect MBK-2 function directly but simply extend the time available for MBK-2 to phosphorylate its substrates.

Only two extragenic suppressors, *cdc-37* and *tat-4*, did not affect the timing of the oocyte-to-embryo transition. We suggest that these loci may encode more direct regulators of MBK-2 activity. Consistent with this possibility, *cdc-37* codes for the *C. elegans* homolog of the Hsp90 co-chaperone Cdc37, which targets Hsp90 to kinases (Beers and Kemphues 2006; Taipale *et al.* 2012). The *cdc-37* allele we recovered is a gain-of-function allele, raising the possibility that it may increase the activity of Hsp90/Cdc37 toward MBK-2(dd5), allowing it to fold more efficiently. The allele we recovered in *tat-4* is also gain-of-function. *tat-4* codes for a seven-transmembrane domain protein predicted to function as an aminophospholipid translocase (Lyssenko *et al.* 2008). The suppressor mutation lies in a poorly conserved intracellular loop. During the oocyte-to-zygote transition, MBK-2 localizes to the cytoplasmic face of the plasma membrane and of endocytic vesicles before redistributing to the cytoplasm (Stitzel *et al.* 2007). It will be interesting to investigate whether TAT-4 regulates the ability of MBK-2 to associate with membranes.

## Conclusion

In summary, we demonstrate that WGS/SNP mapping is an efficient method to identify suppressor mutations. The technique is applicable to classes of mutations, such as semi-dominant alleles, that are difficult to clone by traditional methodologies. The method also inverts the standard approach for characterizing suppressors. In the past, labor-intensive phenotypic analyses of all suppressors were used to prioritize cloning (an even more labor-intensive process). Now, with WGS/SNP mapping, it is possible to clone all the suppressors first and use their molecular identities to guide and prioritize phenotypic characterizations. The recovery of multiple cell-cycle components led us to hypothesize that lengthening of the oocyte-to-zygote transition is a common mechanism of suppression. Such a subtle phenotype may have eluded observation, absent the molecular identities of the suppressors.

## Supplementary Material

Supporting Information
