# Supplementary material for: Identification of Suppressors of mbk-2/DYRK by Whole-Genome Sequencing
Source: G3 (Bethesda). 2013 Dec 17;4(2):231–41. doi: 10.1534/g3.113.009126 (PMC3931558; doi:10.1534/g3.113.009126)
Supplement: Supporting Information [file supp_4_2_231__index.html]

Identification of Suppressors of mbk-2/DYRK by Whole-Genome Sequencing — Supporting Information 

# Identification of Suppressors of *mbk-2/DYRK* by Whole-Genome Sequencing

## Supporting Information for Wang *et al.*, 2014

**Files in this Data Supplement:**

- Supporting Information - Figures S1-S4 and Table S1 (PDF, 1 MB)
- Figure S1 - Mapping of *ax2001* and *ax2013*. (PDF, 742 KB)
- Figure S2 - RNAi of *tat-4* reverses the suppression of *ax2009* but of no other suppressor. (PDF, 472 KB)
- Figure S3 - GFP::MEI-1 and P granules in suppressors. (PDF, 560 KB)
- Figure S4 - Multiple alignment of suppressor genes. (PDF, 568 KB)
- Table S1 - Nematode strains used in this study. (PDF, 413 KB)
