# Supplementary material for: Identification of Suppressors of mbk-2/DYRK by Whole-Genome Sequencing
Source: G3 (Bethesda). 2013 Dec 17;4(2):231–41. doi: 10.1534/g3.113.009126 (PMC3931558; doi:10.1534/g3.113.009126)
Supplement: Supporting Information [file supp_g3.113.009126_009126SI.pdf]

G3: Genes | Genomes | Genetics

Identification of suppressors of *mbk-2/DYRK* by whole-genome sequencing

Yuemeng Wang*, Jennifer T. Wang*, Dominique Rasoloson*, Michael L. Stitzel*#, M., Kevin F. O' Connell&, Harold E. Smith& and

Geraldine Seydoux*

* Department of Molecular Biology and Genetics, Johns Hopkins University School of Medicine, Baltimore MD 21205

# Current address: The Jackson Laboratory for Genomic Medicine
Farmington, CT 06030

& NIDDK, National Institutes of Health, Bethesda, MD 20814

DOI: 10.1534/g3.113.009126

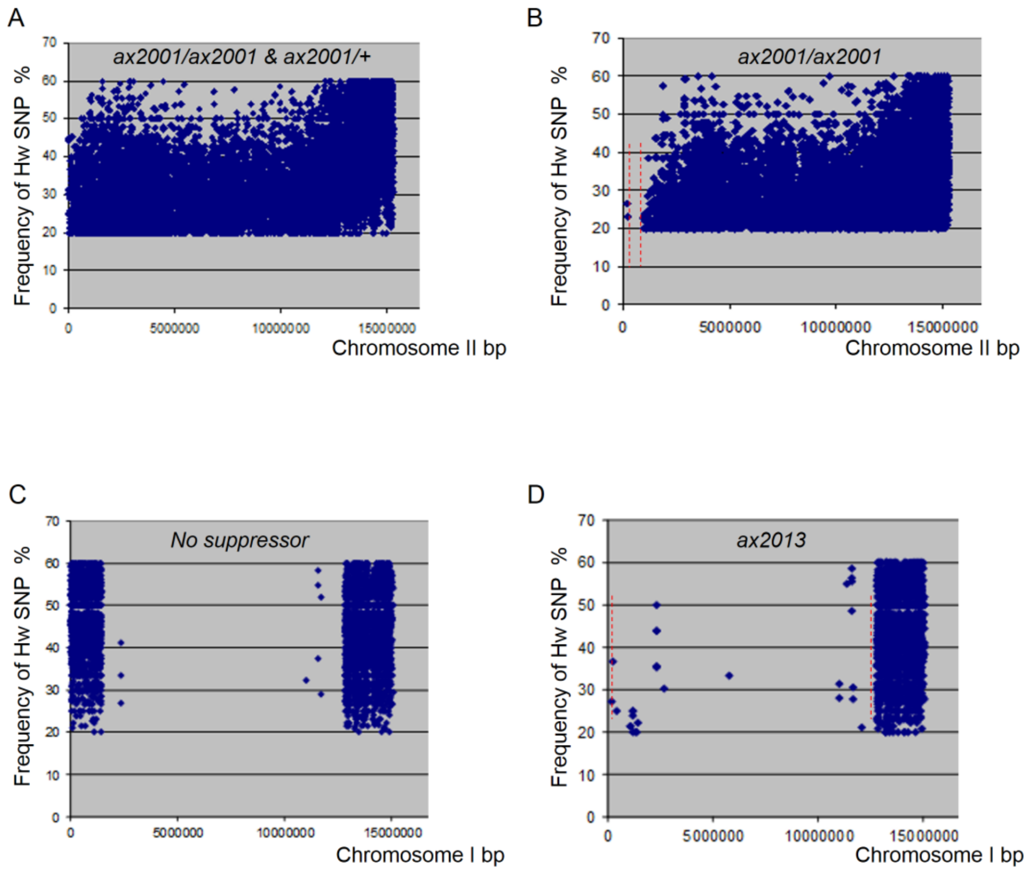

Figure S1 Mapping of *ax2001* and *ax2013*.

(A-D) Graphs showing the percentage of Hawaiian SNPs (Y axis) along the linkage group indicated (X axis). Each dot represents a unique Hawaiian SNP, only SNPs with frequencies between 20-60% are displayed.

(A) 250 progeny of F1 N2/Hw hybrids (*mbk-2(dd5)/mbk-2(dd5)*Hw; *ax2001*/Hw) were grown at 25°C for two generations on one plate and sequenced in one pool. Since ax2001 is dominant, the recombinants are a mixture of *ax2001*/Hw and *ax2001/ax2001* animals. As a result, no distinct region with low Hw SNP frequencies was detected.

(B) Same as in A, except that the recombinants were screened for homozygosity at the suppressor locus before sequencing (Methods). 26 *ax2001/ax2001* F2 recombinants were grown for 2 more generations and sequenced in one pool. A 1Mb region with low Hw SNP frequency was detected at the extreme right end of LGII.

(C) Same as in A showing linkage group I for the introgressed *mbk-2(dd5)*Hw strain. Note the large gap lacking Hw SNPs. This gap is likely due to the N2/Hw incompatibility at the peel-1/zeel-1 locus on LGI and the fact that we used Hw/N2 males to make the introgressed *mbk-2(dd5)*Hw strain .

(D) Same as in C for a population of F2 recombinants carrying suppressor *ax2013*. Note the loss of Hw SNPs denoting linkage to the right end of LGI. However because the large gap in the introgressed strain (C), the entire region demarcated by red lines was scanned for novel mutations.

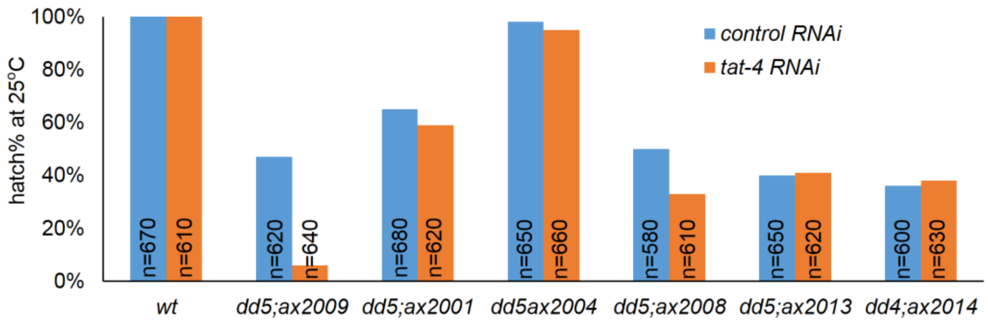

Figure S2 RNAi of *tat-4* reverses the suppression of *ax2009* but of no other suppressor. Percentage of hatched embryos among the progeny of hermaphrodites of the genotypes shown and fed with blank (control) or *tat-4* dsRNA.

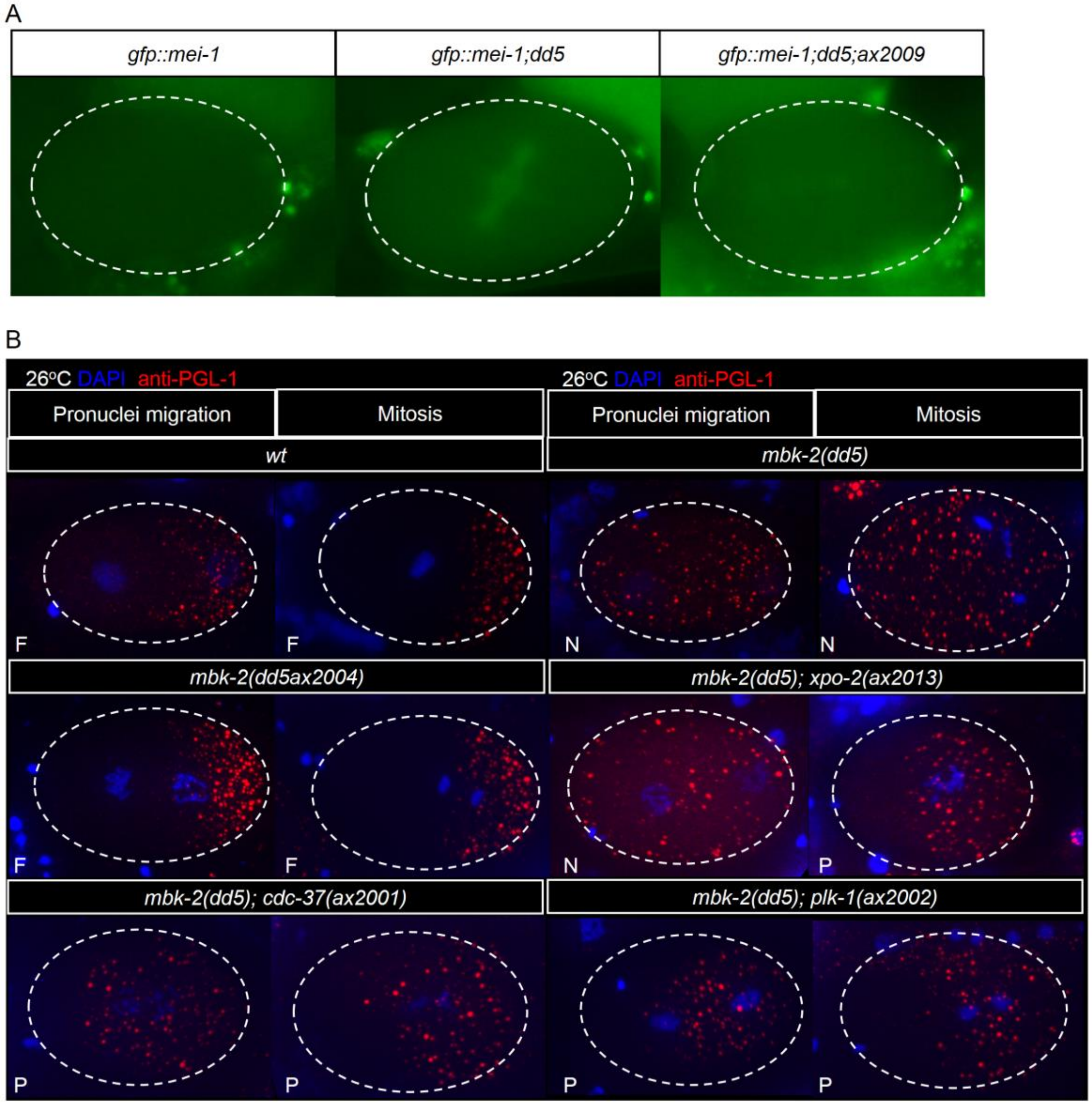

Figure S3 GFP::MEI-1 and P granules in suppressors. (A) Examples of zygotes expressing GFP::MEI-1. Note GFP::MEI-1 on the spindle in the dd5 zygotes. (B) Examples of zygotes stained with anti-PGL-1 antibody. Example of full (F), partial (P) and no segregation (N).

MBK-2

```
CeMBK-2.a       -----------MTLFEPSTSG------NRMG--------------------YRGSSNSSS 23
DmDYRK3-PE      MVGSQEKKNNHIELSETPATDKNNLNTTHLENTQLSKALSPPTSLPQIQIQMINQNLTHT 60
HsDYRK2         -----------MLTRKPSAAAPAAYPTGRGGDSAVRQLQASP---------GLGAGATRS 40
                           :   :..::        :                        . . : :

CeMBK-2.a       GVGSG------------GSGSLMTQSIGGP----NKHLSASHSTLNTAS----------- 56
DmDYRK3-PE      GIAQNNTEKANRHQYRDSGLQYLTRCFEPLAMLNDSKEDFPTQPSNNIANYPGDIQILPI 120
HsDYRK2         GVGTGPPSPIALPPLRASNAAAAAHTIGGSKHTMNDHLHVGSHAHGQIQ----------- 89
                *:. .            ..    :: :       :.:      . .

CeMBK-2.a       --THDMMHSKIPKSPSNESLSRSHTSS------------------SGGSQGGHNSNSGS- 95
DmDYRK3-PE      FDCCEISESIQAISLPNVTSPSKTKDVPGLFLRTISENSKSKSEPECESLISVKESSVME 180
HsDYRK2         --VQQLFEDNSNKRTVLTTQPNGLTTVGKTGLPVVPERQLDSIHRRQGSSTSLKSMEGMG 147
                    :: ..         : .   .                       *  . :. .

CeMBK-2.a       -------------------------NSGFRPEDAVQTFGAKLVPFEKNEIYNYTRVFFVG 130
DmDYRK3-PE      NHTFLFHEQIIMSGQQKCELHEKPKVLVVSPQQVMILYMNKLTPYERTEILTYPQIYFIG 240
HsDYRK2         ----------------------KVKATPMTPEQAMKQYMQKLTAFEHHEIFSYPEIYFLG 185
                                            . *::.:  :  **..:*: ** .*..::*:*

CeMBK-2.a       SHAKKQAGVIGGANNGGYDDENGSYQLVVHDHIAYRYEVLKVIGKGSFGQVIKAFDHKYQ 190
DmDYRK3-PE      ANAKKRPGVYG-PNNSEYDNEQGAYIHVPHDHVAYRYEMLKIIGKGSFGQVIKAYDHKTH 299
HsDYRK2         LNAKKRQGMTGGPNNGGYDDDQGSYVQVPHDHVAYRYEVLKVIGKGSFGQVVKAYDHKVH 245
                 :***: *: * .**. **:::*:*  * ***:*****:**:*********:**:*** :

CeMBK-2.a       QYVALKLVRNEKRFHRQADEEIRILDHLRRQDSDGTHNIIHMLDYFNFRNHKCITFELLS 250
DmDYRK3-PE      EHVALKIVRNEKRFHRQAQEEIRILHHLRRHDKYNTMNIIHMFDYFTFRNHTCITFELLS 359
HsDYRK2         QHVALKMVRNEKRFHRQAAEEIRILEHLRKQDKDNTMNVIHMLENFTFRNHICMTFELLS 305
                ::****:*********** ******.***::*. .* *:***:: *.**** *:******
                                            ax2005 L283F
CeMBK-2.a       INLYELIKRNKFQGFSLMLVRKFAYSMLLCLDLLQKNRLIHCDLKPENVLLKQQGRSGIK 310
DmDYRK3-PE      INLYELIKKNGFKGFSLQLVRKFAHSLLQCLDALYKNDIIHCDMKPENVLLKQQGRSGIK 419
HsDYRK2         MNLYELIKKNKFQGFSLPLVRKFAHSILQCLDALHKNRIIHCDLKPENILLKQQGRSGIK 365
                :*******:* *:**** ******:*:* *** * ** :****:****:***********
                ax2007 V311I                           ax2006 L353F
CeMBK-2.a       VIDFGSSCFDDQRIYTYIQSRFYRAPEVILGTKYGMPIDMWSLGCILAELLTGYPLLPGE 370
DmDYRK3-PE      VIDFGSSCFENQRIYTYIQSRFYRAPEVILGGKYGRAIDMWSLGCILAELLSGHALFPGE 479
HsDYRK2         VIDFGSSCYEHQRVYTYIQSRFYRAPEVILGARYGMPIDMWSLGCILAELLTGYPLLPGE 425
                ********::.**:***************** :** .**************:*:.*:***
                 dd5 D374N
CeMBK-2.a       DENDQLALIIELLGMPPPKSLETAKRARTFITSKGYPRYCTATSMPDGSVVLAGARSKRG 430
DmDYRK3-PE      NESDQLACIIEVLGMPNKNILASSKRSKSFFSPKGYPRYCTVRTMSDGMVVLIGGQSRRG 539
HsDYRK2         DEGDQLACMIELLGMPSQKLLDASKRAKNFVSSKGYPRYCTVTTLSDGSVVLNGGRSRRG 485
                :*.**** :**:****  : * ::**::.*.:.********. ::.** *** *.:*:**
                ax2004 R433C
CeMBK-2.a       KMRGPPASRSWSTALKNMGDELFVDFLKRCLDWDPETRMTPAQALKHKWLRRRLPNPPRD 490
DmDYRK3-PE      KQRGPPCSKSLSKALDGCKDPLFLNFIRGCLEWDADKRLTPSEALKHPWLRRRLPRPPSS 599
HsDYRK2         KLRGPPESREWGNALKGCDDPLFLDFLKQCLEWDPAVRMTPGQALRHPWLRRRLPKPPTG 545
                * **** *:. ..**..  * **::*:: **:**.  *:**.:**:* *******.** .

CeMBK-2.a       ----------------------GLESMGGLADHEVCFIIF-------------------- 508
DmDYRK3-PE      SSGCGGVSGLCSSRNESPVTGQNRNFAAETTASSTSATSISLTIKRENSHSSLRLHHGAV 659
HsDYRK2         ----------------------EKTSVKRITESTGAITSISKLPPPSSSASKLRTNLAQM 583
                                              :    .   :
```

CDC-37

```
CeCDC-37.a       MPIDYSKWKDIEVSDDEDDTHPNIDTPSLFRWRHQARLERMAEKKMEQEKIDKEKGTTSK 60
DmCDC-37-PA      -MVDYSKWKNIEISDDEDDTHPNIDTPSLFRWRHQARVERMAEMDHEKDELKKKRQSYQA 59
HsCDC-37         -MVDYSVWDHIEVSDDEDETHPNIDTASLFRWRHQARVERMEQFQKEKEELDRGCRECKR 59
                   :*** *..**:*****:*******.**********:*** : . *::::.:     .

CeCDC-37.a       KMEELEKKLAAADVTDKSDIQKQIDEVKAQEEAWRKKEA-------ELEEKERLEPWNVD 113
DmCDC-37-PA      RLMDVKERISKKDGDEE-ALKKELEKIEAEGKELDRIES-------EMIKKEKKTPWNVD 111
HsCDC-37         KVAECQRKLKELEVAE--GGKAELERLQAEAQQLRKEERSWEQKLEEMRKKEKSMPWNVD 117
                 :: : :.::   :  :    : :::.::*: :   : *        *: :**:  *****
CeCDC-37.a       TIGHEAFSTSRINKITEKKPQAPKTDEEDTHAMSTFFETHESLLEKMAVLKNGAKSTELF 173
DmCDC-37-PA      TISKPGFEKTVINKKAGRKPDENLSEEEREQRMKQFVKENEKLCQQYGMLRKYDDS-KRF 170
HsCDC-37         TLSKDGFSKSMVNTKPEKTEED--SEEVREQKHKTFVEKYEKQIKHFGMLRRWDDS-QKY 174
                 *:.: .*..: :*. . :. :   ::*   :  . *.:  *.  :: .:*:.  .* : :
```

```
                                                                 ax2001 L221F
CeCDC-37.a       LAEHPHMASEYTANWLTIEALNAAIDFNEEKMKTMAEQCIIIQYLLELSKSLNAVATNTT 233
DmCDC-37-PA      LQEHLHLVGEETANYLVIWSINLEMEEKHELMAHVAHQCICMQYILELAKQLDVDPR--A 228
HsCDC-37         LSDNVHLVCEETANYLVIWCIDLEVEEKCALMEQVAHQTIVMQFILELAKSLKVDPR--A 232
                 * :: *:. * ***:*.* .::  :: :    *  :*.* * :*::***:*.*.. .   :

CeCDC-37.a       VQKQFFKKFEAAEPVYMKHYQDEVKAFEDRLRTRAQTKRDAAMEEAEAEEKAERMKSAPG 293
DmCDC-37-PA      CVSSFFSKIQHCHPEYRAQFDSEIEGFKGRIQKRAQEKIQEAIAQAEEEERKERLG--PG 286
HsCDC-37         CFRQFFTKIKTADRQYMEGFNDELEAFKERVRGRAKLRIEKAMKEYEEEERKKRLG--PG 290
                    .**.*:: ..  *   ::.*::.*: *:: **: : : *: : * **: :*:   **

CeCDC-37.a       GIDPQEVFEQLPEEMRKCFEAHDIEALKGVAQKMDEEVFKYHFDRCIASGLWVPG-KADD 352
DmCDC-37-PA      GLDPADVFESLPDELKACFESRDVELLQKTIAAMPVDVAKLHMKRCVDSGLWVPNAADLE 346
HsCDC-37         GLDPVEVYESLPEELQKCFDVKDVQMLQDAISKMDPTDAKYHMQRCIDSGLWVPNSKASE 350
                 *:** :*:*.**:*:: **: :*:: *: .   *     * *:.**: ******.    :

CeCDC-37.a       DDDDEEAAPAE----------EEPTTSS---------------- 370
DmCDC-37-PA      GDKKEEDDSDD-VAGGEEKTDDAKSESAAKEEPIYTGVSTEDVD 389
HsCDC-37         AKEGEEAGPGDPLLEAVPKTGDEKDVSV---------------- 378
                  .. **  . :          :    *
```

TAT-4

```
CeTAT-4.a         ------------------------------------------------MTTPR------- 5
DmCG33298-PB      MPSANTEDLRRKFLVVQQQRSAPPNLTGDTLSSGIPNASASSATTSLMMTTSTGLGGVAM 60
HsATP10B          ----------------------------------------------MALSVDS------- 7
                                                                 ::.

CeTAT-4.a         ------------------------------------PTHLHRRSSSK------------- 16
DmCG33298-PB      GAGEPERTYVFPGGNPVVGSAPGGGGQGIPGVAMAAPSRGHARSISHGGGAIVGANGRPI 120
HsATP10B          ------------------------------------SWHRWQWRVRDG------------ 19
                                                      . :        .

CeTAT-4.a         ----------------------------------------WVPP---SAPLHNP-----I 28
DmCG33298-PB      KSAMKGHQRAFSQGQITDSPPGSAAPAGRGHSRVGSKTDFILPPGHKEEPAREPSAPTSA 180
HsATP10B          ----------------------------------------FPHCPSETTPLLSP------ 33
                                                                   *   .*

CeTAT-4.a         SIFRGRSNRETGSRVIRPNHLFESP---------------RYELNNYR------------ 61
DmCG33298-PB      TGGRGHSRQASRSESIYTLRRTEAPPWWKRLTLCNYNTGDKFEERSYRTVVPNHTVPPKT 240
HsATP10B          -EKGRQSYNLTQQRVVFPNNSIFHQ---------------DWEEVSRR------------ 65
                       :* . : .. : . .                     :*  . *

CeTAT-4.a         --------DFTDNRISTTKYTFWNFIPLNIWHQVSTKYANLYFIFIAILNWVPFFDAYTR 113
DmCG33298-PB      PKRDHPNGQFVGNKIRTTKYTLLSFIPKNLLEQFH-RVANLYFIFIVLLNWVPEISAFGK 299
HsATP10B          ---------YPGNRTCTTKYTLFTFLPRNLFEQFH-RWANLYFLFLVILNWMPSMEVFHR 115
                           : .*:  *****: .*:* *: .*.  : *****:*:.:***:* :..: :

CeTAT-4.a         YVGLIPICFVLGTTLIKDGIEDYRRYKFDNQINKKTCHVWDRDRCAFRKTEWRYILVGDF 173
DmCG33298-PB      EVAMIPVLFVLGVTAVKDLFEDRRRRASDKRINNTTCRVYDGETERYKKVKWQELRVGDI 359
HsATP10B          EITMLPLAIVLFVIMIKDGMEDFKRHRFDKAINCSNIRIYERKEQTYVQKCWKDVRVGDF 175
                   : ::*: :** .  :** :** :*   *: ** .. :::: .   : :  *: : ***:

CeTAT-4.a         VHISNNQDVPADIILLRSSSESGTCYIETCNLDGETSLKQRMVPAKVIDYSKKDSTFKPP 233
DmCG33298-PB      VHLSNNETVPADILLLRTSDPQGVCYIDTCDLDGETNLKRREV---VRGFEEMQSIFVPS 416
HsATP10B          IQMKCNEIVPADILLLFSSDPNGICHLETASLDGETNLKQRCV---VKGFSQQEVQFEPE 232
                  :::. *: *****:** :*. .* *:::*..*****.**:* *   * .:.: :  * *

CeTAT-4.a         DFTGVVTCEKPDKSIYTIRAKVEFEPGQSDVIIKENMLLRGSRIKNTTFVEGIVVYAGHD 293
DmCG33298-PB      KFVSRVEADAPTTKLYRFHGALIHPTGERVPISTECLLLRESRLKNTDYIEGIVVYAGHE 476
HsATP10B          LFHNTIVCEKPNNHLNKFKGYMEHPDQTRTGFGCESLLLRGCTIRNTEMAVGIVIYAGHE 292
                   * . : .: * . :  ::. : .       :  * :*** . ::**    ***:****:

CeTAT-4.a         TKVMQNNGRAPHKTSGIEKLTNKFIIACFIMLLLMVLYGAITSAVWVGQHPVDEQIPFIL 353
DmCG33298-PB      TKSMLNNSGPRYKRSQVEQQMNIDVIWCVIILLILCVVGAIGCRMWLSSF-THFPVPYLP 535
HsATP10B          TKAMLNNSGPRYKRSKIERRMNIDIFFCIGILILMCLIGAVGHSIWNGTFEEHPPFDVPD 352
                  ** * **. . :* * :*:  *  :: *. :*::: : **:   :* . .  .  .
```

```
CeTAT-4.a          SN--TPRPFIEGFIGIGAFFINYQLLVPISLYITVEIIKALQIYFISNDIQLYDQKSDRA 411
DmCG33298-PB       PN--KLTANMESMWIFWTYIVILQVMIPLSLYVTIELCKILQVFHIHNNVDLFDAETNKQ 593
HsATP10B           ANGSFLPSALGGFYMFLTMIILLQVLIPISLYVSIELVKLGQVFFLSNDLDLYDEETDLS 412
                   .*     . : .:  : : ::  *:::*:***:::*: *  *::.: *:::*:* :::

CeTAT-4.a          IDCRSLSIPEELGTVTHVLSDKTGTLTENMMIFRNCAFDETDYGSNGS-----QSNPDKP 466
DmCG33298-PB       TECRAMNITEELGQIQHIFTDKTGTLTENKMIFRRCVVNGSDYNHPPSELEKIYSKPGAP 653
HsATP10B           IQCRALNIAEDLGQIQYIFSDKTGTLTENKMVFRRCTIMGSEYSHQENAKRLETPKELDS 472
                    :**::.*.*:** : ::::********* *:**.*..  ::*.   .      .:   .

CeTAT-4.a          VKSDELYQRILTSMQNPVQ-----------KHFFANILLNNSVVVNHIPHTDVLELGNFD 515
DmCG33298-PB       APPLIPNDNLNSDMAQLTQGTYLTPHAQRIQEFLVVLAICNTVIVGAAPHRDMMNASGII 713
HsATP10B           DGEEWTQYQCLSFSARWAQDP----ATMRSQKGAQPLRRSQSARVPIQGHYRQRSMGHRE 528
                           .  :   . .*           :.    :   ::. *    *    . .

                                             ax2008 G544S
CeTAT-4.a          GGVYNIGNSCFYDVTEEKYKQKLAAIGKGGVDDDDVSRP-DELGLPTTSIQFDDRLTVIV 574
DmCG33298-PB       E-VQQIGNS-PANLKHGKQRQKLLASSTSTTTTTTIING-PTTQPQVVSIP-ADRYIRLA 769
HsATP10B           SSQPPVAFSSSIEKDVTPDKNLLTKVRDAALWLETLSDSRPAKASLSTTSSIADFFLALT 588
                        :. *   :      :: *     .       :           .:    *    :.

CeTAT-4.a          EEDTPSDSPAP-----------------------------------DASPRDLPETPTP- 598
DmCG33298-PB       ESRSVTPSPPPNLLFALPAQSHQPTL-------------SPISSSAESSPNSESESPSPP 816
HsATP10B           ICNSVMVSTTTEPRQRVTIKPSSKALGTSLEKIQQLFQKLKLLSLSQSFSSTAPSDTDLG 648
                     :   *...                                   :: .   .. .

CeTAT-4.a          ---------TSPIYRPLSSLSSFSRKLSTVVRRSILRPISDIIPVRKRLISFKQQAMNP- 648
DmCG33298-PB       MKNKSLSNSISPTGRAKAVINSKITSIATFLNAKTQGKRMKLPSSKTGTIYRTADGRPL- 875
HsATP10B           ESLGANVATTDSDERDDASVCSGGDSTDDGGYRSSMWDQGDILESGSGTSLEEALEAPAT 708
                                ..  *  : : *    .        .       .:    .

CeTAT-4.a          --------YEAESPDELALIEGAALYDYVLLERAATSVTISTPEK-AEKRYEILLTLPFD 699
DmCG33298-PB       --------YEAESPDELALVNAAYSYDCCLLNRSPNQILVSMPMAGATREYEILKVLPFD 927
HsATP10B           DLARPEFCYEAESPDEAALVHAAHAYSFTLVSRTPEQVTVRLPQG-TCLTFSLLCTLGFD 767
                           ******** **:..*  *.  *:.*:. .: :  *   :   :.:* .* **

CeTAT-4.a          ATRKRMSVIVNSQKGP--LMYCKGADSAIISRLS---------SDSLESKRVQDLKDHLD 748
DmCG33298-PB       SSRKCMSIVVRQIGSQEIVLYTKGADSSIMPVLVP------CSHNSPEGILREQTQQLLD 981
HsATP10B           SVRKRMSVVVRHPLTGEIVVYTKGADSVIMDLLEDPACVPDINMEKKLRKIRARTQKHLD 827
                   : ** **::*.        ::* ***** *:  *             :.          :. **

CeTAT-4.a          NYAKKGLRTLCFAMKYISKEDFEDFLDSYRFLMEDATSEREKMLSEKADELETNLKLSGV 808
DmCG33298-PB       RYAREGLRILVMAKRTLNSADYTDWWARH-QEIEMSLENRERRLRDSFAKLESNLTLLGA 1040
HsATP10B           LYARDGLRTLCIAKKVVSEEDFR-RWASFRREAEASLDNRDELLMETAQHLENQLTLLGA 886
                    **:.*** * :* : :.. *:       .     * : .:*:. * :.  .**.:*.* *.

CeTAT-4.a          TGIEDRLQDGVPDTLRALRDAGIQVWVLTGDKLETAQNIATSSGLFHPQRSLKVIET--- 865
DmCG33298-PB       TGIEDRLQDGVPETIASLLSAGISVWVLTGDKPETAINIAYSAKLFTQQMELIRLTARSR 1100
HsATP10B           TGIEDRLQEGVPDTIATLREAGIQLWVLTGDKQETAVNIAHSCRLLNQTDTVYTINTENQ 946
                   ********:***:*: :* .***.:******* *** *** *. *:      :  : :

CeTAT-4.a          ------------ETDAEEASESAG--------------LNIIMSPAAIRLAQDGN----- 894
DmCG33298-PB       DAAETAINFYLTDMENDKTTSTLG--------------YGQSLRKKQRALVVDGKTLTFI 1146
HsATP10B           ETCESILNCALEELKQFRELQKPDRKLFGFRLPSKTPSITSEAVVPEAGLVIDGKTLNAI 1006
                               : .  .  .. .                          *. **:

CeTAT-4.a          --------AHLMEALKKAKTVLCYRMTPSEKATIVNTVKKRIKGNVLAIGDGANDVPMIQ 946
DmCG33298-PB       LDPKSKLILPFLRLSKRCASVLCCRSTPLQKAYLVKVVKEELNLRTLAIGDGANDVSMIQ 1206
HsATP10B           FQG--KLEKKFLELTQYCRSVLCCRSTPLQKSMIVKLVRDKLRVMTLSIGDGANDVSMIQ 1064
                                ::.  : . :*** * ** :*: :*: *:..:.  .*:********.***

CeTAT-4.a          AAHVGIGIAGKEGLQAAMACDFAIARFKFLSRLLLVHGHWSYYRLANTFLYFLYKNANAV 1006
DmCG33298-PB       MADVGVGISGQEGMQAVMAADFTLPRFRYLERLLLAHGYWCYDRLSRMILYFFYKNAAFV 1266
HsATP10B           AADIGIGISGQEGMQAVMSSDFAITRFKHLKKLLLVHGHWCYSRLARMVVYYLYKNVCYV 1124
                    *.:*:**:*:**:**.*:.**::.**:.*.:***.**:*.* **:.  .:*::***.  *
CeTAT-4.a          FIIFYYQFYNGASGTNIVDPIWGVIYPIIFTSVQPVVVGVLDQDYDDQTLMNKPELYVIG 1066
DmCG33298-PB       FLIFWYQLYCGFSGQVMMDQMYLMLYNLIFTSLPPLAIGVYDKRVAEDLLLKNPYLYKNG 1326
HsATP10B           NLLFWYQFFCGFSSSTMIDYWQMIFFNLFFTSLPPLVFGVLDKDISAETLLALPELYKSG 1184
                    ::*:**:: * *.  ::*    ::: ::***: *:..** *:    : *:  * **  *
```

```
CeTAT-4.a         RENQLYTWKHFFRDVIDGIYQAAVIYYVAYLTLDNSTSSLWEMGFYIATSSILVNSGHLA 1126
DmCG33298-PB      RLGVAYRPHDFWLILLDALYQSLVIFFVALCAYAESDVGIWEFGTTITASCLFANLVHCA 1386
HsATP10B          QNSECYNLSTFWISMVDAFYQSLICFFIPYLAYKGSDIDVFTFGTPINTISLTTILLHQA 1244
                  : .  *    *:  ::*.:**: : :::.  :   *  .:: :*  * : .: .   * *

CeTAT-4.a         LQVRYW----------------------------------------------HWQLVAL 1139
DmCG33298-PB      IEIRSWTVLHVLSIVLSLGSFYLFAIVYDSVCMNCFGVRSSYWVIFVCFASAVHWLVIML 1446
HsATP10B          MEMKTWTIFHGVVLLGSFLMYFLVSLLYNATCVICNSPTNPYWVMEGQLSNPTFYLVCFL 1304
                  :::: *                                              .: :  *
```

PLK-1

```
CePLK-1.a       ---MNRLPNIAKPPQKSNQR------------KEKAPPEVPALIADKDRGTYYEKGRFLG 45
DmPOLO-PB       ---------MAAKPEDK-------------------STDIPDRLVDINQRKTYKRMRFFG 32
HsPLK1          MSAAVTAGKLARAPADPGKAGVPGVAAPGAPAAAPPAKEIPEVLVDPRSRRRYVRGRFLG 60
                         :*  * .                    . ::*  :.*      * : **:*
                                     ax2008 L74F   ax2002 V87M
CePLK-1.a       KGGFAHCYELTNRATREVVAGKVVPKSMLVKQYQRDKMTQEVQIHRELGHINIVKLFNFF 105
DmPOLO-PB       KGGFAKCYEIIDVETDDVFAGKIVSKKLMIKHNQKEKTAQEITIHRSLNHPNIVKFHNYF 92
HsPLK1          KGGFAKCFEISDADTKEVFAGKIVPKSLLLKPHQREKMSMEISIHRSLAHQHVVGFHGFF 120
                *****:*:*: :  * :*.***:*.*.:::*  *::* : *: ***.* * ::* :..:*

CePLK-1.a       EDNLNVYITLELCARRSLMELHKRRKAVTEPEARYFTHQIVDGVLYLHDLNIIHRDMKLG 165
DmPOLO-PB       EDSQNIYIVLELCKKRSMMELHKRRKSITEFECRYYIYQIIQGVKYLHDNRIIHRDLKLG 152
HsPLK1          EDNDFVFVVLELCRRRSLLELHKRRKALTEPEARYYLRQIVLGCQYLHRNRVIHRDLKLG 180
                **.  :::.**** :**::*******::** *.**:  **: *  ***  .:****:***

CePLK-1.a       NLFLNDDLVVKIGDFGLATTVNGD-ERKKTLCGTPNYIAPEVLNKAGHSFEVDIWAVGCI 224
DmPOLO-PB       NLFLNDLLHVKIGDFGLATRIEYEGERKKTLCGTPNYIAPEILTKKGHSFEVDIWSIGCV 212
HsPLK1          NLFLNEDLEVKIGDFGLATKVEYDGERKKTLCGTPNYIAPEVLSKKGHSFEVDVWSIGCI 240
                *****: * ********** :: : ***************:*.* *******:*::**:

CePLK-1.a       LYILLFGQPPFESKSLEETYSRIRHNNYTIPSIATQPAASLIRKMLDPEPTRRPTAKQVQ 284
DmPOLO-PB       MYTLLVGQPPFETKTLKDTYSKIKKCEYRVPSYLRKPAADMVIAMLQPNPESRPAIGQLL 272
HsPLK1          MYTLLVGKPPFETSCLKETYLRIKKNEYSIPKHINPVAASLIQKMLQTDPTARPTINELL 300
                :* **.*:****:. *::** :*:: :* :*.     **.::  **:.:*  **:  ::

CePLK-1.a       RDGFFKSGFMPTRLPVSCLTMVPKFGGHETSMMEENVAPRGVDARSQRPLNGRAGLSALP 344
DmPOLO-PB       NFEFLKGSKVPMFLPSSCLTMAPRIG----------SNDTIEDSMHRKPLMEMNGIRPDD 322
HsPLK1          NDEFFTSGYIPARLPITCLTIPPRFS----------IAPSSLDPSNRKPLTVLN------ 344
                .  *:... :*  ** :***: *::.                *.  ::**

CePLK-1.a       QHIVSNNADR---ERAQQQAAEATFREPEDAYLSQLFHQVAVLLEQRIPGLEEEEAALDG 401
DmPOLO-PB       TRLESTFLKANLHDAITASAQVCRHSEDYRSDIESLYQQLTNLINGKPR--ILQGNLGDE 380
HsPLK1          KGLENPLPER---PREKEEPVVRETGEVVDCHLSDMLQQLHSVNASKPS--ERGLVRQEE 399
                  : .   .          ..      *   . :..: :*:  :   :            :

CePLK-1.a       YQSPECLPVFWISKWVDYSDKYGIGYQLCDNSVGVLFNDNSRIMLDQAGNELTYIEKSNK 461
DmPOLO-PB       NTDPAAQPLFWISKWVDYSDKYGFGYQLCDEGIGVMFNDTTKLILLPNQINVHFIDKDGK 440
HsPLK1          AEDPACIPIFWVSKWVDYSDKYGLGYQLCDNSVGVLFNDSTRLILYNDGDSLQYIERDGT 459
                  .* . *:**:***********:******:.:**:***.::::*     .: :*::...

CePLK-1.a       EHYFSMHSGEMPGLLNKKVTLLKYFRSYMNDHLVKAGEGSEQRAGDDLARLPTLRVWFRT 521
DmPOLO-PB       ETYMTTTD--YCKSLDKKMKLLSYFKRYMIEHLVKAGANNVNIESDQISRMPHLHSWFRT 498
HsPLK1          ESYLTVSS--HPNSLMKKITLLKYFRNYMSEHLLKAGANITPREGDELARLPYLRTWFRT 517
                * *::  .       * **:.**.**: ** :**:*** .     .*:::*:* *: ****

CePLK-1.a       KSAIVLHLSNGTVQINFFNDHVKMMMCPLMQAVTFIDQNKRMLTYKLNNLQRNGCPEKFL 581
DmPOLO-PB       TCAVVMHLTNGSVQLN-FSDHMKLILCPRMSAITYMDQEKNFRTYRFSTIVENGVSKDLY 557
HsPLK1          RSAIILHLSNGSVQINFFQDHTKLILCPLMAAVTYIDEKRDFRTYRLSLLEEYGCCKELA 577
                 .*:::**:**:**:* *.** *:::** * *:*::*:::  **::. : . *  :.:

CePLK-1.a       HRLKYAKTMIERLMSDANVVSQNPARQPDMPRSMAAARSASAGSRGPNQAASHLPQSASG 641
DmPOLO-PB       QKIRYAQEKLRKMLEKMFT----------------------------------------- 576
HsPLK1          SRLRYARTMVDKLLSSRSASNRLKAS---------------------------------- 603
                 :::**:  : :::..  .
```

FZY-1

```
CeFZY-1         ---------------MNNKGR--TPGSAGRTVRSSAQQNGLTMR-------KRDMTPTRN 36
DmFZY-PA        MSQFNFVSDLQNALIMDGETR-GPAPRWKKKLEASLNGSVNTTRSVLSVSYNTSFSGVQA 59
HsCDC20         MAQFAFESDLHSLLQLDAPIPNAPPARWQRKAKEAAGPAPSPMR-----AANRSHSAGRT 55
                               ::      ..    :. . :      . *       : . :  :

CeFZY-1         TNLLPNATFVGDRFLGVRLDQDELDHANHLMTSKLYSNKENLNNSMSEP----------- 85
DmFZY-PA        PTKTPGKSSEGKTKKSNTTPSKTPGGGDRFIPNRAATNFELAHFLVNKDSGDKSDEENDK 119
HsCDC20         PGRTPGKSSS----KVQTTPSKP--GGDRYIPHRSAAQMEVASFLLSKE----------- 98
                .   *. :            ..    .:: :. :  :: *     :.:

CeFZY-1         ----NSPEKKSVEGEALKQMMRHKSTGALTDADDGDRILCYKKNLAPPPAIGYINQAKVL 141
DmFZY-PA        ATSSNSNESNVQASAHKGDRQKLISEVAQVGDSKGGRILCYQNKAPAAPET-HNNPLKVV 178
HsCDC20         ------NQPENSQTPTKKEHQKAWALNLNGFDVEEAKILRLSGKPQNAPEG-YQNRLKVL 151
                        : :       :  :  :        .  :**  . :   .*   : *  **:

CeFZY-1         YSTNSVINPASSVKKSTRHVKETATKVLDGPGLTKDLYSRHLDWGCHNWVAVALGHELYL 201
DmFZY-PA        YSIK----TPISTKSGSRYIPTTSERILDAPDFINDYYLNLMDWSADNIVAVALGSCVYL 234
HsCDC20         YSQKA---TPGSSRKTCRYIPSLPDRILDAPEIRNDYYLNLVDWSSGNVLAVALDNSVYL 208
                ** :    .. * :.  *::   . ::**.* : :* * . :**.. * :****.  :**

CeFZY-1         WNTETCVIKNLFEDNAPTNEGLITSVRWSQEGRYISLGYASGAVKIYDPNRPKTTEYVRE 261
DmFZY-PA        WNAQTGNIEQLTEFEE---GDYAGSLSWIQEGQILAIGNSTGAVELWDCSK------VKR 285
HsCDC20         WSASSGDILQLLQMEQP--GEYISSVAWIKEGNYLAVGTSSAEVQLWDVQQ------QKR 260
                *.:.:  * :* : :          *: * :**. :::* ::. *:::* .:       :.

CeFZY-1         LRTLRVGGASRCASIAWRKQGVMTCGYKSGDIVNHDVRISQHVVSSWGGDNGHCRDVTAL 321
DmFZY-PA        LRVMDG-HSARVGSLAWN-SFLVSSGSRDGTIVHHDVRAREHKLSTLSG---HTQEVCGL 340
HsCDC20         LRNMTS-HSARVGSLSWN-SYILSSGSRSGHIHHHDVRVAEHHVATLSG---HSQEVCGL 315
                ** :    ::* .*::*. . :::.* :.* * :****  :* ::: .*   * ::* .*

CeFZY-1         EWSADENMCVSGSSDRTAKIWDGRHVRGSTVIQDPEPMFTIDEHTGQVRTAQFCSFRDGI 381
DmFZY-PA        KWSTDFKYLASGGNDNLVNVWS---AASGGVGTATDPLHKFNDHQAAVRALAWCPWQPST 397
HsCDC20         RWAPDGRHLASGGNDNLVNVWP---SAPGEGG--WVPLQTFTQHQGAVKAVAWCPWQSNV 370
                .*:.* .  .**..*. .::*       .       *: .: :* . *::  :*.:: .
                                                                ax2014 D434N
CeFZY-1         LATGGGINDGTVKLWDVKRQFQKVRELNVCETGGVGGIVFNRPYSEMLTAS--DDGFLRI 439
DmFZY-PA        LASGGGTADRCIKFWNVN---NGTLMKSVDSKSQVCSLLFSRHYKELISAHGFANNQLTI 454
HsCDC20         LATGGGTSDRHIRIWNVC---SGACLSAVDAHSQVCSILWSPHYKELISGHGFAQNQLVI 427
                **:***  *  :::*:*    . .     *    . * .:::.  *.*:::.    :. * *

CeFZY-1         YRFNANYKLSHEIQASNEPIMDLVGSPFDEVLIGDMEETLKVFQLFNVDKSTNILDRTAP 499
DmFZY-PA        WKYPTMVKQADLTGHTSRVLQMAMSPDGSTVISAGADETLRLWNCFAPDPLASKKAVSTS 514
HsCDC20         WKYPTMAKVAELKGHTSRVLSLTMSPDGATVASAAADETLRLWRCFELDPARRREREKAS 487
                ::: :  * :.    :.. :   :..    *  .  :***:::. *  *        .:.

CeFZY-1         KNVGLNVR---- 507
DmFZY-PA        KGKQSVFRQSIR 526
HsCDC20         AAKSSLIHQGIR 499
                      .:
```

SUCH-1

```
                             ax2010 L17F
CeSUCH-1        -MPPKKAQTRRIVSLDSLFGHTLLNITGEPVTP--------------TKIAIFQLIRTLF 45
DmIDA           MILCLHFAGKSKIGFRGPKLTMNLFEELDTLDPSFKLEPPRIETPTAHKITVLILLKQYV 60
HsANAPC5        -MASVHESLYFNPMMTNGVVHANVFGIKDWVTP--------------YKIAVLVLLNEMS 45
                 :   :         : .       :    : : *             **::: *:.

CeSUCH-1        HAHFGVGAVPSLKPFDKDEKTRVFTVLYGLIIMKSEISYDDFRCIVRILNDGLGRSIYYR 105
DmIDA           INKKNC---LDTGISMRTQRRRMFYMLVFKLIQEQDKSYNELHSLLTTGKYKLDTLMLES 117
HsANAPC5        RTG-------EGAVSLMERRR--LNQLLLPLLQGPDITLSKLYKLIEESCPQLAN----S 92
                          .       .:   :  *   ::   : : ..:  ::      *
CeSUCH-1        FVTSMEKLAHGEDQIEMLFEDAFYTAKRPNHEKVLKRE--DSWLDELTFMNSNSFLYIWI 163
DmIDA           FEKAMSEFCAGSIEALFDFSEIQNIDEILNENYGISQFSMVGVYVRRVGVVLERLSFPEM 177
HsANAPC5        VQIRIKLMAEGELKDMEQFFDDLS-DSFSGTEPEVHKTSVVGLFLRHMILAYSKLSFSQV 151
                .   :. :. *. :    * :     .  . :  : :    .   .   :  . : :  :
```

```
CeSUCH-1        KRVMMQYTRTSQNGTFEIAEKFQKWIISG--------------------TLEIISHPILS 203
DmIDA           MDMYKNVCSYYERGVRAAASGSRMAVAGGILHREETPPPNPITEKPEDPKPKKKVEAVAR 237
HsANAPC5        FKLYTALQQYFQNGEKKTVEDADMELTS----RDEG-----------ERKMEKEELDVSV 196
                  :        :.*    ..     : .                     . :     :

CeSUCH-1        GINRALPTEIDCSIRARHWCAAQLRLVQLCPTKAMSYFQILDWCDTIHRRHHDVVDVHLL 263
DmIDA           IAQERNPLSKWAPKQAKFFINKQSELLENNERKALPPLELQKKVQEIIHDLPLTTTPYFL 297
HsANAPC5        REEEVSCSGPLSQKQAEFFLSQQASLLKNDETKALTPASLQKELNNLLKFNPDFAEAHYL 256
                  :.       .  :*..:   *  *::    **:.  .: .  : : :     .  : *

CeSUCH-1        RAAIFVQLKNSSDAVKSLKQFFDMSMLEITENSKHALETLKLMSPSQVALRFGPILQGRV 323
DmIDA           GYMNQLRVRDYYNALSALHRALDRSPVR----LMSQEKGY----------QYFCVNLAVL 343
HsANAPC5        SYLNNLRVQDVFSSTHSLLHYFDRLILTGAESKSNGEEGYGRS------LRYAALNLAAL 310
                     ::::   .:  :* : :*   :          :            ::  :  . :

CeSUCH-1        HRIFGERQIASALFAESIQQSQVNVDDMCNRIANMEVTINSIYMSGPLLQRLSGETFAAG 383
DmIDA           HATFGHRDEALAALRESIMLAQEHGDKRS-----LNLANTWYCLLRDELPLSA------- 391
HsANAPC5        HCRFGHYQQAELALQEAIRIAQESNDHVC-----LQHCLSWLYVLG------------- 351
                *  **. : *   : *:*  :*   *. .     ::   .   :

CeSUCH-1        KKEEESVENERRVQQNSVHAAVDLNVPTLRSLQKNFREDYELHAFLVSMCKFLLCIQDMM 443
DmIDA           -------------VQKSVQDANEVDGSLLQNYTLALHFAVKLGTVAGYQPLRLFDLLQHS 438
HsANAPC5        ------------------QKRSDSYVLLEHSVKKAVHFGLPR-AFAGKTANKLMDALKDS 392
                                  :   :      :.    .:      :.       *:   .

CeSUCH-1        DGKFFKHNSTADYVSVGFHRLRLLLDMNNKGFVLQAFANAIMTSGLIQSGMYHQAKRVAE 503
DmIDA           DNLTNRN-NFADHASEALALRSAVWSAYGRHELAALYSQVLLTG---------------- 481
HsANAPC5        DLLHWKH-SLSELIDISIAQKTAIWRLYGRSTMALQQAQMLLSMNSLEA----------- 440
                *    :: . ::  . .:     :    .:  :    :: :::

CeSUCH-1        TMIVSNCDAPNSPILETESHAVAGVNLVYSLAAVGDYEKAQKTIDILKNRFPENINWMAA 563
DmIDA           ---------RDRFGSGSAGLGSALASFALWLQLQGEPQLSKVLLHHAKQRFP---RLPSA 529
HsANAPC5        -------VNAGVQQNNTESFAVALCHLAELHAEQGCFAAASEVLKHLKERFPP--NSQHA 491
                          .     : . . *   :.      *    :. :.    *:***  .   *

CeSUCH-1        RHVDICSKIVNFERNFLLNKYSECSRHLAGLETSAPLEFVLRKSLLLAATGKLAEAVLLL 623
DmIDA           EGWMISQCHVVIQAGIYQCRWHDALKACDQLYLIDPSDSLFQRASIYVAKREFFNARRLL 589
HsANAPC5        QLWMLCDQKIQFDRAMNDGKYHLADSLVTGITALNSIEGVYRKAVVLQAQNQMSEAHKLL 551
                .   :..  : ::  :   ::  .      :    . : : ::: :  *  :: :*  **

CeSUCH-1        GTYECGDVR---GSMRIHMQMATIHTAYGQFETAEIQIQEAGKVAVN----AHFLDANLL 676
DmIDA           DKLAVQDNIPFLLRMRVQVLLGYCGMANGRFSSETTMLLLRVSEEMS-EAQMDYELALVD 648
HsANAPC5        QKLLVHCQKLKNTEMVISVLLSVAELYWRSSSPTIALPMLLQALALSKEYRLQYLASETV 611
                 .            * : : :.         ..            :.     .:  :

CeSUCH-1        VVRRVGSLMLGRFMAREAYQVLHALSAKIEHFGSFIEKAIYHVSMARCLRLMHKD----P 732
DmIDA           LLLALQLLLLG--MPQKAYQAIKRCMHDIHINGGLYERAKTDFVFVRCLLAVNQNDAEKR 706
HsANAPC5        LNLAFAQLILG--IPEQALSLLHMAIEPILADGAILDKGRAMFLVAKCQVASAASYDQPK 669
                :   .  *:**  :..:* . ::     *   *.: ::.   . ..:*      .

CeSUCH-1        RVHLKQCKAQIIGNKWPAMEKL-----LLTELTILHHSGGLYPDEQKEMKAKERFGKIEA 787
DmIDA           KAQLLKSQVI-LQRAAQSFKKLSAHAKVLDVYVFLAQRFNEFGDRNLRNKYAGEFRRYFT 765
HsANAPC5        KAEALEAAIENLNEAKNYFAKVDCKERIRDVVYFQARLYHTLGKTQERNRCAMLFRQLHQ 729
                :..  :.    : .    : *:     :     :  :      . : . :    * :

CeSUCH-1        DFPGPCTWMFI-- 798
DmIDA           DHPIPREYLGSP- 777
HsANAPC5        ELPSHGVPLINHL 742
                : *     :
```

EMB-30

```
                                                        ax2003 splicing∨
CeEMB-30        MDFSIVKTQILNNRRTFRTPFKVSSMCFSSQNDLIALGSKTGEILLKRTSWKMIWKTNIN 60
DmAPC-4         ----MAQTSSMKLLGARNMSCIVERMEWNNKMDLIAYGTEKGEVVIQRLN--------WQ 48
HsANAPC4        MLRFPTCFPSFRVVGEKQLPQEIIFLVWSPKRDLIALANTAGEVLLHRLAS-------FH 53
                     .    :.     . .  :  : :. : **** ..  **::::*           :
```

```
CeEMB-30        MIQAVGTECKLDSSVSALHFSPDGRFLAAATSK-GIIHLLDVETGKVRFSVKAASEKIAK 119
DmAPC-4         KIVTFPTPGED-VRVRSLSWQMDETLLAVGYSN-GKVALLDAESGTI-ISGLIYEDDIKK 105
HsANAPC4        RVWSFPPNENTGKEVTCLAWRPDGKLLAFALADTKKIVLCDVEKPES-LHSFSVEAPVSC 112
                 : :. .  :    * .* :  *  :** . :.   : * *.*.    :     .  :

CeEMB-30        LHWNCVREKPFISNLGEFTTRIKNVEAIEGAIELAETTPNISQEEIAFVYQRLDEDGSSF 179
DmAPC-4         VYFSKAIN---------------SQENLGTYTCNVKDKHRRFLPKLQPMTN-IDPCLKTL 149
HsANAPC4        MHWMEVTV---------------ESSVLTSFYN-AEDESNLLLPKLPTLPKNYSNTSKIF 156
                :::  .                 . . :      .:   .    ::  : :  .    . :

CeEMB-30        KHEDAHKESLERTLISTETFRESLQNTILLATDDMDSKIIVLVAGVFPYMEIDISDTLLQ 239
DmAPC-4         DQKS-----------------FPKGSPCFLVVIMRSGKVHLLLLGALQAGSIDLTQHILH 192
HsANAPC4        SEENSDEI-------------IKLLGDVRLNILVLGGSSGFIELYAYGMFKIARVTGIAG 203
                ..:.                     .   *     ...  .:   .     .*     :

CeEMB-30        YNQSLLMLYDMHYSSAFGGVSFLATTYGPFLDCKQNELKPPGAEPKKDGQGCHTLLFNVK 299
DmAPC-4         PHEFDVYDVRMNGDCNAIYALLRDGQELILLHFQNQVLQDCMAPMLELATHCAHILETKN 252
HsANAPC4        TCLALCLSSDLKSLSVVTEVSTNGASEVSYFQLETNLLYSFLPEVTRMARKFTHISALLQ 263
                          ::  .    .          :. : : *     .   . .      :    :

CeEMB-30        LNINSSLWDTALRYIRLLFGFNLYSISLETTKRNWEEQIDNLHSLFDTKTKAVKIGNVLL 359
DmAPC-4         Y-INDTQQCLTEAWETVQLEMDN-----------------KLTRYANSQ-PYGVISAHFL 293
HsANAPC4        Y-INLSLTCMCEAWEEILMQMDS-----------------RLTKFVQEKNTTTSVQDEFM 305
                  ** :       :  : : ::                 .*     : :      :   ::

CeEMB-30        EMLLSGSTDAAGEAFLERGLGTDGLDKIELFATKHMPEVCRIARGQLSTSARNLCFQRCE 419
DmAPC-4         ELHVFGFATFEVEEFLFETLSEKGFKKIANSVDLSLSNLQSLVFKQLNGAAINMFYFLNT 353
HsANAPC4        HLLLWGKASAELQTLLMNQLTVKGLKKLGQSIESSYSSIQKLVISHLQSGSESLLYHLSE 365
                .: : * :    : :* . *  .*:.*:         ..:  :.  :*. .: .: :

CeEMB-30        FSTSLSRYAKFIQLKDDDSFLYDEDPPAYLSESNIWLNTLEEKINILDMKTRHLGIQCLT 479
DmAPC-4         IAGFGRMSHFFESL------ISPDVANEAMRACGGFVLKVHELQRTIDTLAYDMKLFHAW 407
HsANAPC4        LKGMASWKQKYEPLG-----LDAAGIEEAITAVGSFILKANELLQVIDSSMKNFKAFFRW 420
                :         :  *      :        :   . :: . .*  . :*    .:

CeEMB-30        MMQELGHLVKWISMTKPFAKTMKVNALMKIKRMNIAKILLYIVRNFIPDPEAVKDIENRL 539
DmAPC-4         MIFTILRLSHQE-----IPDDLVLTEEENIAMADFICAMEPELDDRSDDEDELDSQSQTP 462
HsANAPC4        LYVAMLRMT----------EDHVLPELNKMTQKDITFVAEFLTEHFNEAPDLYNRKG--- 467
                :   : ::            .   :    ::   ::          .      :  .

CeEMB-30        FNLKELVSKQKKKDLEDKFDEVEYLYRLERIAEVERKKLIETFHEKFRFADLDDDDLTPL 599
DmAPC-4         PPARSKFNLERVG---------QYLDNAYLTQLYPRDP-VQLWEEMVTDNECLSN--CKL 510
HsANAPC4        ----KYFNVERVG---------QYLKDEDDDLVSPPNTEGNQWYDFLQNSSHLKE--SPL 512
                    . .. ::           :**             .   : : :  .   .  .:    *

CeEMB-30        LFQELDHPIDIFTDSMMEQNDSSPFLEEDEGEPEQEEQKPDEEPEPEGEPQPACDLDRVG 659
DmAPC-4         FVPHDVNLSLVQQRDKMFN------------AIDAVFHKPTESISGSFKVSSSTVICNDLP 558
HsANAPC4        LFPYYPRKSLHFVKRRMEN------------IIDQCLQKPADVIG---KSMNQAICIPLY 557
                :.    .         * :              :   :** :       :          :

CeEMB-30        SFFEKELSQKALEVLPKCDETFDCVLNERGSRMNSIEIQKMGILRVLEDLAATLSSPQKI 719
DmAPC-4         PMEPGEDHRDRDLVTCTYYVNEASRTDMLACTISGQ---EAMILEFSRAGDECVRCT-RI 614
HsANAPC4        RDTRSEDSTRRLFKFPFLWNNKTSNLHYLLFTILEDSLYKMCILRRHTDISQSVSNG-LI 616
                     *            .  .  .     :      :  **.        :     *

CeEMB-30        HCDKAGNQKKLEVSFIYEITSTPTHQGYQDAKISSVTPSYYKRHPFNQLSGMGRSIQVSV 779
DmAPC-4         TLEPGLFTTSVSEDF--CYLRFVDLQFYNESSLSILAQSINAGPGMRPHS---FFIQFSL 669
HsANAPC4        AIKFGSFTYATTEKVRRSIYSCLDAQFYDDETVTVVLKDTVGREGRDRLL---VQLPLSL 673
                  . .         ..          * *:: .:: :  .                : .*:

CeEMB-30        LQKNEFSSIVIVPTSDVLPEDENEVNDHVEKLKRCYEFEKIDVTVDKSSAENRPPGETDA 839
DmAPC-4         TAALNYSTQHRMG--PLVKLSEATVSQSIHDIADGAAFKGLDGFSDMLAVS--------- 718
HsANAPC4        VYNSEDSAEYQFTGTYSTRLDEQCSAIPTRTMHFEKHWRLLESMKAQYVAGN-------- 725
                    : *:   .        .*        . :      :. ::        .
```

```
CeEMB-30        MEVDSNELRIDVDLGPVVPEYDTLIELSQVHPLHHGELVLLGKFTSAQEGTGVMSQMMRS 899
DmAPC-4         ---------------------------------------------GSRKVATVLSDRKRK 733
HsANAPC4        ---------------------------------------------GFRKVSCVLSSNLRH 740
                                                             . :: : *:*.  *

CeEMB-30        ISSYPHEIPEDKDVFAKNSSEDGSSEIPIETLIIHPTIQLAAYLHDDGSKITIGDMKPEV 959
DmAPC-4         MTIFEMEIEEE----EDDTEMSQASFLDIS--------------------------KESVL 764
HsANAPC4        VRVFEMDIDDEWELDESSDEEEEASNKPVK--------------------------IKEEV 775
                :  :  :* ::     .. . . :*   :.                          :  :

CeEMB-30        PPIADYEEKTVRKTFRDYQRRRERYREDMGVMNMNDVQYDVLVQEAMDSGRDLTDNGESD 1019
DmAPC-4         AGVPDAEKQEA------------------------------------------------- 775
HsANAPC4        LSESEAENQQAGAAALAPEIVIKVEKLDPELDS--------------------------- 808
                   .: *:: .
```

MAT-2

```
CeMAT-2         ----------------------------------------------MRKYVLFFISG--- 11
DmSHTD-PA       QLKAAVHPDDDHLTAICVMDQDALRVYCSNGEDFLANLDFPVSQLWQTKYGLLLEKDSSN 240
HsANAPC1        DKSEKAYSSNEVEKCICILQSSCINMHSIEGKDYIASLPFQVANVWPTKYGLLFERSASS 195
                                                                ** *::  .

CeMAT-2         ---------------------------------------------NNDSQIWATTPNTPR 26
DmSHTD-PA       ALISHMS--IPMPRLFSMSHPLHEACPVVLKT----ATGSTGYMTEPEYTVVFTTEESDL 294
HsANAPC1        HEVPPGSPREPLPTMFSMLHPLDEITPLVCKSGSLFGSSRVQYVVDHAMKIVFLNTDPSI 255
                                                             :    :   . :.

CeMAT-2         VIARGGLERNIHTRTLARMVNEDAPGTSTPAAQS--RLQTTASPFHRTHLTQMCRRGDTN 84
DmSHTD-PA       VMLYDAKFFKHFVARLRKVTPEEINYVSQQMELG--QTLMGPRSMAGNSFSSTKQTGATP 352
HsANAPC1        VMTYDAVQNVHSVWTLRRVKSEEENVVLKFSEQGGTPQNVATSSSLTAHLRSLSKGDSPV 315
                *:  ..      .  * ::  *:   .      .       . .     : .  : . .

CeMAT-2         ASLLRDFTRMIRDTPRN-----FSKNTQHGNDRDFGDLERDPD----------------- 122
DmSHTD-PA       KATNLSFAARNINTTTTGMGNQFGLSQSQSFSGVLGQSNRASLGTPLSQLQSSISQQSMS 412
HsANAPC1        TSPFQNYSSIHSQSRSTSSPSLHSRSPSISNMAALSRAHSPALGVHSFSGVQRFNISSHN 375
                 :   .::    ::  .     .. . . .    :.  .  .

CeMAT-2         ------------------------VDLLLSKVCLECVYVEPKEGAIPKANKIFISNFLSD 158
DmSHTD-PA       VKDMRKLTHVKP------------AKPIEPELCMEHIWTENIYGTQREFCEMATRAFIHT 460
HsANAPC1        QSPKRHSISHSPNSNSNGSFLAPETEPIVPELCIDHLWTETIT-NIREKNSQASKVFITS 434
                                        .. : .::*:: ::.*       :  .     *:

CeMAT-2         MYINLVSVTGEVMKIIPIWKNAETTRKNLLEKGKHEPCVVDCVDAAFVMKSGITVVLGSD 218
DmSHTD-PA       DLVGQTFLCYLLARSCRLQLVRLTGYGRGEVQLSTHASTLAAKDAVGLKRMHMIAVLDPS 520
HsANAPC1        DLCGQKFLCFLVESQLQLRCVKFQESNDKTQLIFGSVTNIPAKDAAPVEKIDTMLVLEGS 494
                   .   :   :     :                     : . **. : :     **  .

CeMAT-2         FTTAMFGGNERIAPIFIK-------------------------------------EMSNQRV 243
DmSHTD-PA       GSLLLYTGTVLISKVHITPFLAPTSIPTPLVTPMTAAPSPSASPAPSHVKTPMAAAGPAS 580
HsANAPC1        GNLVLYTGVVRVGKVFIP------GLPAPSLTMSNTMPRPSTPLDGVSTPKPLSKLLGSL 548
                 .  :: *   :. :.*

CeMAT-2         GRKFRLFSFAENR----------------------------------------------- 256
DmSHTD-PA       GIPSGSSSFVEVRRSSLLPTKAPGDVAAFEEELHMLSPIQPQPVSYTQRQAHNVCKSLRD 640
HsANAPC1        DEVVLLSPVPELRDSSKLHDSLYNEDCTFQQLGTYIHSIR-------------------D 589
                .      .. * *

CeMAT-2         ------IFAVNEMRCIVVEIPETVTCKSATELMRTCFLHLDRDLSRKLLIKWRSVKRDVD 310
DmSHTD-PA       PAGNRLTLVYATGRMLRIALPFLNDTRLLTRCVATLRQVLSPTQFLDFVIRWYSDRNPPG 700
HsANAPC1        PVHNRVTLELSNGSMVRITIPEIATSELVQTCLQAIKFILPKEIAVQMLVKWYNVHSAPG 649
                       :       : : :*     .     : :    *      .::::* . :   .

CeMAT-2         TERLDLDRKEMIDVAIFMLDN--------------VGVRVTNVVAQERADSPEGHGGKQM 356
DmSHTD-PA       SRNYSIEQEWLLFRSTLLALMGLTAAPDVDAGENYARCATPPLHTQFGGGATATESSDGS 760
HsANAPC1        GP--SYHSEWNLFVTCLMNMMGYNTDRLAWTRNFDFEGSLSPVIAPKKARPSETGSDDDW 707
                    . . :  :  : ::                      . : :   . ..   ...
```

```
CeMAT-2         RPRMSDSEVLSMMR----------QFFEEMTFRPKSEVITEDGYKCHLSVELDPNGEGFV 406
DmSHTD-PA       SCSSNSTLGGQDEPKKRRIYNDCDDFTDDDWEFLLLQTTLAPCGADGHSYSVNIGALLFR 820
HsANAPC1        EYLLNSDYHQNVES----------HLLNRSLCLSPSEASQMKDEDFSQNLSLDSSTLLFT 757
                    ..    .             .: :        :.          . .:: .   *

CeMAT-2         HTQDLLHAFHSQCEDWSINTMMHSILLELIPYAYLLAKVMNYRAFEEYYVQLFKHLLSQI 466
DmSHTD-PA       MIPAIFFSLHLLYEDLKLDADFYGALPYLATFLHQLAIDMQLESYVLHYILDFPELSNRT 880
HsANAPC1        HIPAIFFVLHLVYEELKLNTLMGEGICSLVELLVQLARDLKLGPYVDHYYRDYPTLVRTT 817
                    ::. :*   *: .::: :   :  *      **  ::  .:  :*   :  *

CeMAT-2         AIEFKIPPEIHEKFVGAIHIPKPCWSLNNVIAHIICERTTPETMESIPKFISKSSVRLLT 526
DmSHTD-PA       GKLSLLGAEHGAMMLHQELLRVPAPSVFAQLEHIIVGEEEVMPYTFLECVNERSRILLQL 940
HsANAPC1        GQVCTIDPGQTGFMHHPSFFTSEPPSIYQWVSSCLKGEG-MPPYPYLPGICERSRLVVLS 876
                .    : .     :     :     *:   :   :  .    .   :  . .:* : :

CeMAT-2         ILAVGRKFIGMG---------------------------TNIDMDCERWLGKDWKRRIGL 559
DmSHTD-PA       VSLVTHGHERLN------YWWQLLEIPGAVQAN--FTRRSKRNITADAPRSHQMLQLLLA 992
HsANAPC1        IALYILGDESLVSDESSQYLTRITIAPQKLQVEQEENRFSFRHSTSVSSLAERLVVWMTN 936
                :         :                            :  .  .    ..     :

CeMAT-2         SGDILKSFRRIMNGKSSNSAGRASQLIELFEIGSITIDFMVLAVKVLMLKFQTDAFAGAQ 619
DmSHTD-PA       MRLTRRDIERFPAAVHLIVAEALEEARLSPPMGCSMATYELILRPELAAHAQLPFLETST 1052
HsANAPC1        VGFTLRDLETLPFGIALPIRDAIYHCREQPASDWPEAVCLLIGRQDLSKQACEGNLPKGK 996
                     :.:. :  .          .       .       ::    *  :     :  .

CeMAT-2         -----------SIEPKKCIYATADEMIS---------------IAHLRWKNDIRMHNVQL 653
DmSHTD-PA       GQPHCGRVYKEDSLSARCPPTGGSETDSPAQLRRDDMDNMDTKLLRLRFPDDMRVDEVRR 1112
HsANAPC1        -----------SVLSSDVPSGTETEEED------DGMNDMNHEVMSLIWSEDLRVQDVRR 1039
                           .  .         *  .               :  * : :*:*:.:*:

CeMAT-2         MLNSSRPILIATNILRKNEDDNMKELQDRFLTQTSYRTFSQPFGRAFLDFRTAVPSLLTS 713
DmSHTD-PA       LLNSSEPVVIEVQQAPGTSDHEFIEEQEKQLFALCSRTMTLPVGRGMFTLRTMLPRPSES 1172
HsANAPC1        LLQSAHPVRVNVVQYPELSDHEFIEEKENRLLQLCQRTMALPVGRGMFTLFSYHPVPTEP 1099
                :*:*:.*: : .      .*.:: * ::. *   . **:: *.**.:: : :  *    .

CeMAT-2         IYIPRLNVGGMIYPSRVTCDPP--TTEIFKLCTEWGNFYNSLASALRIGSSETVRIDNEW 771
DmSHTD-PA       LTMPKLCLLGKEPLKGTTIEMQ--QIEFPANMQMWPSFHNGVATGLKISPQ-AQDIDSNW 1229
HsANAPC1        LPIPKLNLTGRAPPRNTTVDLNSGNIDVPPNMTSWASFHNGVAAGLKIAP--ASQIDSAW 1157
                : :*:* : *      .* :      :.      * .*:*.:*:.*:*..  :  **. *

CeMAT-2         IVMVSKN--IKSTAVIGGMTLGFGLNGHLAPFNMYHAHQMLSTFDKFHSVALLIGLSASN 829
DmSHTD-PA       IVYNKPKTHSHNALEHAGFLMALGLNGHLKTLSFMSVYKYLVKCDEMTNVGLLLGISAAH 1289
HsANAPC1        IVYNKPK-HAELANEYAGFLMALGLNGHLTKLATLNIHDYLTKGHEMTSIGLLLGVSAAK 1216
                **  . :   . :   .*: :.:******  :     :. * . .:: .:.**:*:**::

CeMAT-2         FTTCDVQIHKILATYLSFLMGPTPLEIKLDFTIQTAAISGLGLLFADSGNMTIAKKLVNE 889
DmSHTD-PA       RGTMDTKTTKLLSVHLEALLPATAMELDIPQSTQVAAIMGVGLLYQGSAKRHIAEVLLQE 1349
HsANAPC1        LGTMDMSITRLLSIHIPALLPPTSTELDVPHNVQVAAVVGIGLVYQGTAHRHTAEVLLAE 1276
                  * * .  ::*: ::  *: .*. *:.:  . *.**: *:**:: .:.:   *: *: *

CeMAT-2         IGRAPNRDEEPVTDRNAYKLSAGFSLGLIMLGKGNGSASTVIPFKQNIPPMSQRLIYMMN 949
DmSHTD-PA       IGRPPGPEMENSIERESYAMTAGLSLGLVTLGQGESPAG-----LRDLQLPDTLHYYMVG 1404
HsANAPC1        IGRPPGPEMEYCTDRESYSLAAGLALGMVCLGHGSNLIG-----MSDLNVPEQLYQYMVG 1331
                ***.*. : *   :*::* ::**::**:: **:*..  .       ::   .    **:.

CeMAT-2         GMRRDKCVFLPQVAPPVVNDVPNLPFSNGGMMTSSQVANHVKESEYINIHQSAEPAAIAL 1009
DmSHTD-PA       GVKR--------------------PIGGSQKEKYRLASFQVREGDTVNIDVTAPGATLAL 1444
HsANAPC1        GHRR--------------------FQTGMHREKHKSPSYQIKEGDTINVDVTCPGATLAL 1371
                * :*                       .    .    : :::*.: :*:. :.  *::**

CeMAT-2         GMMFMKMNNEFIANALALPGTITELERLKPDSMYSRVLAQCLVMWDSIEPTHDFVKSLIP 1069
DmSHTD-PA       GLMFFNSGNAAIAEWMQPPDSRYLLDMVRPDFLLLRTISRGLILWQDVRPDNAWFQAQFP 1504
HsANAPC1        AMIYLKTNNRSIADWLRAPDTMYLLDFVKPEFLLLRTLARCLILWDDILPNSKWVDSNVP 1431
                .::::: .*  **: :  *.:   *: ::*: :  *.::: *::*:.: *   :..: .*
CeMAT-2         PVIREYATAALHFGVPIRRDEDGEEVHEAINDAEEKYWAEIVDKGTVSQTFLYAVSAACM 1129
DmSHTD-PA       RALRAHLKLPFYENEYAPEDYD-------------------VDYEAISQAYCNIMAGAAF 1545
HsANAPC1        QIIRENSIS--LSEIELPCSED-------------------LNLETLSQAHVYIIAGACL 1470
                  :*               . *                   ::  ::**:.   ::.*.:
```

```
CeMAT-2         AIALKFSSCGGPNEKNIVNTAFRIIEYYTKIVMPDGKSNKDMGSIRMCIYSGAYTRTSCL 1189
DmSHTD-PA       CIGLKYAGTEN-------LVAFATLRSVIKDFLR-------FPSRPMGECAGRTTVESCL 1591
HsANAPC1        SLGFRFAGSEN-------LSAFNCLHKFAKDFMT-------YLSAPNASVTGPHNLETCL 1516
                .:.::::.  .         **  :.   * .:           *      :*  .  :**
                               ax2012 V1208M
CeMAT-2         SMLITAMAILRVGTGDLEVMRYARLLRLCDKPESDWIATGKKHFEQMVAHQALGILMLGE 1249
DmSHTD-PA       MVLLISISLVFAGSGNCEILRIIRFLRSRVGPQYPHITYG----SHMAIHMSLGLLFLGA 1647
HsANAPC1        SVVLLSLAMVMAGSGNLKVLQLCRFLHMKTG---GEMNYG----FHLAHHMALGLLFLGG 1569
                 ::: ::::: .*:*: ::::  *:*:          :  *     ::. * :**:*:**

CeMAT-2         GRYAFKKDDLSIALTIISTFPTIPQSVSDNSHYHQPLRFLWSMAVEPRLLVPFDIAESCV 1309
DmSHTD-PA       GRFTISQTPESIAALVCAFFPKFPIHSNDNRYHLQALRHLYVLAVEPRLFLPRDIDTNKL 1707
HsANAPC1        GRYSLSTSNSSIAALLCALYPHFPAHSTDNRYHLQALRHLYVLAAEPRLLVPVDVDTNTP 1629
                **:::.    ***  : : :* :*   .** :: *.**.*: :*.****::* *:  .

CeMAT-2         VEVDVTIVMKPKDGNEPIVYKEKAPYLLPPLEDLQSISIGGGNYQLVHISLQ---SEDQV 1366
DmSHTD-PA       CLANISVLEVG---ATELRRLPIAPCILPVLSTLQQVVVDDENYWPVCFERSRNWDQLEK 1764
HsANAPC1        CYALLEVTYKGTQWYEQTKEELMAPTLLPELHLLKQIKVKGPRYWELLIDLSKGTQHLKS 1689
                  . : :                ** :** *  *:.: : . .*  : :. .   .. :

CeMAT-2         KVMKDIMTIGQGRVMLKRYGVDSSEMKIKEATTLYDDTPSLMSMFNNEDTAVELDEYEIQ 1426
DmSHTD-PA       ALEMSAPIDIKKRTGCLSHLEDPDRLKSMLAQTLTMEQSICWQIDMNDLQQFASERMVKQ 1824
HsANAPC1        ILSKDGVLYVKLRAGQLSYKEDPMGWQSLLAQTVANRNSEARAFKPETISAFTSDPALLS 1749
                 :  .     : *.    :  *.   :   * *:    .    :  :    .  :    .

CeMAT-2         CMMEKIDEGINLNSSDEYPNVQIELSCVRDVTERTTMDLAQLQKRSLKLLSES------- 1479
DmSHTD-PA       FLSRCLDTKGTDLSPPELMKRHQVMLLFYNAVVKDRMHFLPVYLTLYDHVTKS----MPN 1880
HsANAPC1        FAEYFCKPTVNMGQKQEILD--LFSSVLYECVTQETPEMLPAYIAMDQAIRRLGRREMSE 1807
                      .   .  .  *  .       . : . :   .:        . : .

CeMAT-2         -LDLWQDEVN---------------------------------VSNTINGLADAVQDMQI 1505
DmSHTD-PA       NIDVWQMKLIDAYLSRSQESE----HPLISVELIQMMQELFKQEMEDSTRELCLPLREFLS 1937
HsANAPC1        TSELWQIKLVLEFFSSRSHQERLQNHPKRGLFMNSEFLPVVKCTIDNTLDQWLQVGGDMCV 1868
                  ::** ::                                    :.::          ::
```

MUS-101

```
CeMUS-101         MEAPPAPKKARRSEVSRMQDESVLCDDDEEDSPFTLYFVDLPNEPTVKEIRNLEELFKTA 60
DmMUS-101-PA      -----------------------MSRNDQE--PFLVKFLKSSDN--SECFFKALESIKEL 33
HsTOPBP1          -----------------------MSRNDKE--PFFVKFLKSSDN--SKCFFKALESIKEF 33
                                         :. :*:*  ** : *:. .::   : : :  * :*

CeMUS-101         KAVGIMPEWIDSDALEDLQKSEDFFVLPCFRGKLFRKLQARKLKVYGPPIVLESIEDGKQ 120
DmMUS-101-PA      QSEDYLQIITDEEALKIRENDKSLYICDRFSGTVFDHLKQLGCRIVGPQVVTFCMRHQQC 93
HsTOPBP1          QSEEYLQIITEEEALKIKENDRSLYICDPFSGVVFDHLKKLGCRIVGPQVVIFCMHHQRC 93
                  ::   :    :.:**:  ::...:::   * * :* :*:    :: ** :*  .:.. :

CeMUS-101         LPQWNHPVYSSVFQDVKISFTGLNLTKKQELYEKIGWMCGVVGDALYHETTHLVTEKAEQ 180
DmMUS-101-PA      VPRAEHPVYNMIMSDVTVSCTSLDKDKREEVHKYVQMMGGRVYRDLNVSVTHLIAGEVGS 153
HsTOPBP1          VPRAEHPVYNMVMSDVTISCTSLEKEKREEVHKYVQMMGGRVYRDLNVSVTHLIAGEVGS 153
                  :*: :****. ::.**.:* *.*:  *::*::: :  * * *   *  ..***:: :. .

CeMUS-101         TEKYKAAVNNSIKLMRIGWIDDLWETSQTTMGRFSALSRDSVNSYALRVFEGLEMAITSI 240
DmMUS-101-PA      -KKYLVAANLKKPILLPSWIKTLWEKSQEKK--ITKYTDVNMEDFKCPIFLGCIICVTGL 210
HsTOPBP1          -KKYLVAANLKKPILLPSWIKTLWEKSQEKK--ITRYTDINMEDFKCPIFLGCIICVTGL 210
                   :** .*.* .  ::  .**. ***.** .   ::  :  .::.:   :* *  :.:*.:
                                                  ax2011 S273N
CeMUS-101         DGADRTNFIQLIEDHGGKVPGTMSKTRCSYLISDKITGVKYAKAVEWKSMQIVQSRWIRK 300
DmMUS-101-PA      NGIHRKTVQQLTAKHGGQYMGQLKMNECTHLIVQEPKGQKYECARRWN-VHCVTLQWFHD 269
HsTOPBP1          CGLDRKEVQQLTVKHGGQYMGQLKMNECTHLIVQEPKGQKYECAKRWN-VHCVTTQWFFD 269
                   * .*. . **  .***:  * :. ..*::** :: .* **  * .*: :: *  :*: .


CeMUS-101         CVDLGHLVDAGKYHPKYLTADHIR--SSTPKRDAN--VTESVPDISSIAGHGG-RLGTSS 355
DmMUS-101-PA      SIEKGFCQDESIYKAETRVEAKMVPDTSTPTAQSN-AESHTLADVSHISNINGSCVNETM 328
HsTOPBP1          SIEKGFCQDESIYKTEPRPEAKTMPNSSTPTSQINTIDSRTLSDVSNISNINASCVSESI 329
                  .:: *.  * . *:.:     :    :***. : *   :.::.*:* *:. ..  :. :
```

```
CeMUS-101         FNSSIPQMDQSYQHQSGSSFISFASTSKIPSSTFTNNDSTLGR----------SGSGVR- 404
DmMUS-101-PA      FGSTTSKLECSLENLENLDISMFQAPEDLLDGCRIYLCGFSGRKLDKLRRLINSGGGVRF 388
HsTOPBP1          CNSLNSKLEPTLENLENLDVSAFQAPEDLLDGCRIYLCGFSGRKLDKLRRLINSGGGVRF 389
                   .*  .::: : :: .. ..  * :...: ..       .  **          **.***

CeMUS-101         DGISTPIARVQT------------------------------------------------ 416
DmMUS-101-PA      NQLNEDVTHVIVGDYDDDVRQFWSKSSHRPHVVGAKWLLECFTKGYILPEESYIHTNYQP 448
HsTOPBP1          NQLNEDVTHVIVGDYDDELKQFWNKSAHRPHVVGAKWLLECFSKGYMLSEEPYIHANYQP 449
                  : :.  :::* .

CeMUS-101         --TPLIRYPTTQTASVQNIADVISDP---IDELRKNIDDGLGDLFEN------------- 458
DmMUS-101-PA      AGIAVSDQPGNQTAVLDK-SGSFSKSALVPAERLQQADEDLLAQYGNDDSTMVEAKLSEA 507
HsTOPBP1          VEIPVSHKPESKAALLKKKNSSFSKKDFAPSEKHEQADEDLLSQYENGSSTVVEAKTSEA 509
                     .:   * .::* :.:  . :*.      *  :: *:.*   : *

CeMUS-101         ------------------------------CMFYICGVDESRMEKWRRFLNETGATRVAK 488
DmMUS-101-PA      LEPEVGPCPGSAHREPCDDSTHISVQEENKSSVSHCILDDSTVREEGLFSQKSFLVLGFS 567
HsTOPBP1          R-----PFNDSTHAEPLNDSTHISLQEENQSSVSHCVPDVSTITEEGLFSQKSFLVLGFS 564
                                                . .  *  * * : :   * :::  .   .

CeMUS-101         FTSATNVVVVSPNQQERITIRKHLHQEDIAIVTVG---------------WVVECVKQRK 533
DmMUS-101-PA      VENKCNIVDIIREHAGKIVSLPSRIVADYAVVPLLGCEVDVTVGEVVTNTWLVTCIDNQT 627
HsTOPBP1          NENESNIANIIKENAGKIMSLLSRTVADYAVVPLLGCEVEATVGEVVTNTWLVTCIDYQT 624
                   .  *:. :  ::  :*         * *:*.:               *:* *:. :.

CeMUS-101         MISVEG------------------------------------------------------ 539
DmMUS-101-PA      LVDPKSNPLFTPVSVMSGVTPLEDCVISFSQCVGAERDSLVFLANHLGASVQEFFVRKAN 687
HsTOPBP1          LFDPKSNPLFTPVPVMTGMTPLEDCVISFSQCAGAEKESLTFLANLLGASVQEYFVRKSN 684
                  :.. :.

CeMUS-101         ----------------------------------YQWTENTADESQSSQSQLQPVRPQPLP 566
DmMUS-101-PA      AKKGMLASTHLIVKEPTGSKYEAAKKWSLPAVNISWLLETARIGKRADENHFLVDNAPKQ 747
HsTOPBP1          AKKGMFASTHLILKERGGSKYEAAKKWNLPAVTIAWLLETARTGKRADESHFLIENSTKE 744
                                                   *  :**  .: ::..   :   .

CeMUS-101         RTSSKSTIPYGKTSGP--------------SSTSTQVGIFSYHTYCVHCSVDQEVSDD-- 610
DmMUS-101-PA      EQVLETKIPNGVSSNPDLPAHPDAHLEIHRKKAVTPLDMNRFQSRAFRAVISQQRGQDPT 807
HsTOPBP1          ERSLETEITNGINLNSDTAEHPGTRLQTHRKTVVTPLDMNRFQSKAFRAVVSQHARQVAA 804
                  .   :: *. * . ..              ... * :.:  ::: ..:. :.*.  :

CeMUS-101         ---LKEKIPLNGGKLMDDPD--YAEFVIFGHSGPMHELIS-------------------F 646
DmMUS-101-PA      FPPVRQPLTKEPSLHLDTPSKFLSKDKLFKPSFDVTDALAALETPNAASQ-KRKLSSPLS 866
HsTOPBP1          SPAVGQPLQKEPSLHLDTPSKFLSKDKLFKPSFDVKDALAALETPGRPSQQKRKPSTPLS 864
                     : : :  : .  :* *.   ::  :*  *  : : ::

CeMUS-101         DAVVTDFYIYASIGNNRFLNRSCYPLFVPLPRPPILIFNQRGFQLKCKDPFLRDR---VR 703
DmMUS-101-PA      EVIVRNLTVALANSSRNTDSHSASPQLKGAHLEEEETRKPLDSVVVCVSKKLSKKQSELN 926
HsTOPBP1          EVIVKNLQLALANSSRNAVALSASPQLKEAQSEKEEAPKPLHKVVVCVSKKLSKKQSELN 924
                  :.:* :: :  : ....    *. * :             :     : * .  * .:   :.

CeMUS-101         DIIEDNGGRIVEQLEP--KDFIIMIDAEDNPPRYHSR------TLDFSWIIASVSRCRLQ 755
DmMUS-101-PA      GVAASLGAEYRWSFDETVTHFIYQGRANDSNREYKSAKERGVHIVSEHWLLECAQEYKHL 986
HsTOPBP1          GIAASLGADYRWSFDETVTHFIYQGRPNDTNREYKSVKERGVHIVSEHWLLDCAQECKHL 984
                  .:  . *.    .::   ..**    .:*.  .*:*        :.  *:: .... :

CeMUS-101         PIDNFLYKNN-------TRPLSGFQRDDDLWEKCSREKNNSTQEMEVEVEDRHRVQDLPV 808
DmMUS-101-PA      PESLYPHTYNPKMSLDINTVQDGRLCNSRAPLAVSASKDDGPDHLSVEGNETNTMGTNDK 1046
HsTOPBP1          PESLYPHTYNPKMSLDISAVQDGRLCNSRLLSAVSSTKDDEPDPLILEENDVDNMATNNK 1044
                  * . : :. *        .   .*   :.      *  *:: .: : :* :: . :

CeMUS-101         ETGNGN-IGDMTTPYVNPYFPDLRKPYTMNLQLDGVSEYINNMESPARETQETLTTSRVG 867
DmMUS-101-PA      ESPLLNGSGRDDCKGALTQALEMRENFQKQLQEIMSATCIVKTPAQKTCMSRSSCNSASS 1106
HsTOPBP1          ESAPSNGSGKNDSKGVLTQTLEMRENFQKQLQEIMSATSIVKPQGQRTSLSRSGCNSASS 1104
                  *:   *  *       . .   ::*: :  :**    :  * :  .     ..:  .*  .
CeMUS-101         SILRKAVVNTGRN---EDFEDEPSTCHLIRPRVPENRKTVSSTPVLVRDVNRSMRYMPMD 924
DmMUS-101-PA      TPDSARSVRSGRSRVLEALRQSRQAVPDVNTEPSQNEQIIWDDPTAREERARLASNLQWP 1166
HsTOPBP1          TPDSTRSARSGRSRVLEALRQSRQTVPDVNTEPSQNEQIIWDDPTAREERARLASNLQWP 1164
                  :      ..:**.   * :.:. .:   :... .:*.: : . *.  .:  *    :
```

```
CeMUS-101         ESFADQNQEHEDLNR--------------------------------------------- 939
DmMUS-101-PA      SDPTQHSELQVEIKMPDDSPSRKPVYHSEIAEQASC------VTQAPGHPGSEEPEPPVA 1220
HsTOPBP1          SCPTQYSELQVDIQNLEDSPFQKPLHDSEIAKQAVCDPGNIRVTEAPKHPISEELETPIK 1224
                  .  :: .: : :::

CeMUS-101         ------------------------------------RYAMNPRFLLSVSNMDPQRAADLQ 963
DmMUS-101-PA      ERPLIPEPQAPAVASPLAKPPVAPQPADKIETQEETHRKVKKQYVFQMSSLNSQERIDYC 1280
HsTOPBP1          DSHLIPTPQAPSIAFPLANPPVAPHPREKIITIEETHEELKKQYIFQLSSLNPQERIDYC 1284
                                                      :  :: ::::.:*.::.*.  *

CeMUS-101         ETIMKLGGTIEREFN--KDVTHLIASNMQRAPKVLCSIAAGKWCLTPDYVTKSAEVGRWL 1021
DmMUS-101-PA      RLIKDLGGSVIEKQCSDPSCTHMVVGYPLRNEKYLASMAAGKWVLHRSYLDACKTAGRFV 1340
HsTOPBP1          HLIEKLGGLVIEKQCFDPTCTHIVVGHPLRNEKYLASVAAGKWVLHRSYLEACRTAGHFV 1344
                  . * .*** : .:       **::..   *  * *.*:***** *  .*:  .  .*:::

CeMUS-101         DEKSFEWTREKLLACSKKNESLKERDSRKVSENLAAVCGLWRQRVAEMPVTFSVSMENRQ 1081
DmMUS-101-PA      QEEDYEWGSSSILDALPDVTEHQQK--------LALAAMRWRKRIQQS------QESGIV 1386
HsTOPBP1          QEEDYEWGSSSILDVLTGINVQQRR--------LALAAMRWRKKIQQR------QESGIV 1390
                  :*:.:**  ..:*         :.:        ** ..  **::: :       . ..

CeMUS-101         NGAFSDWRCVIHLDDKRAGVFSSILEAGGAIVHSISEYVEVSTLKPNIVLASKEFPWNPQ 1141
DmMUS-101-PA      EGAFSGWKAILRVDRPREAGFKRLLQAGGAKVLSG---HPEPLLKDATHLFCDFNKLKPD 1443
HsTOPBP1          EGAFSGWKVILHVDQSREAGFKRLLQSGGAKVLPG---HSVPLFKEATHLFSDLNKLKPD 1447
                  :****.*: ::::*  * . *. :*::*** * .       . :*    * ..    :*:

CeMUS-101         SAVLLKRDNIPIYVFDILYDFLIDRDNLNRTKFWHNVYMKA------------------- 1182
DmMUS-101-PA      DCRVFIAEATAQNMVCLKTEYIADYLMLESPPCADNYRVSEAALFH--NKKGGPGLPQKR 1501
HsTOPBP1          DSGVNIAEAAAQNVYCLRTEYIADYLMQESPPHVENYCLPEAISFIQNNKELGTGLSQKR 1507
                  .. :   :  .  :  :  ::: *    : .   .*  :
```

XPO-2

```
CeXPO-2           -------MEQIGAALQQTLEPDAAIRKRGEEALRSLQSNPGYIIQILQLVVNEQQQIAPQ 53
DmCAS-PA          MEVTEANLQLLAGYLQQTLSADPNVRRPAEKLLESTELQQNYPILLLNLIDKAQM--DMT 58
HsCSE1L           MELSDANLQTLTEYLKKTLDPDPAIRRPAEKFLESVEGNQNYPLLLLTLLEKSQ---DNV 57
                         :: :   *::**..*. :*: .*: *.* : : .* : :* *: : *

CeXPO-2           IRIAAAVALKNFVKRNWGPAPEV----EMGQEDEEQFRSMLLEAMFNTKSNVQEILSNAL 109
DmCAS-PA          TRVAGAIAFKNYIKRNWAAHLDSDGPDRIHESDRNTIKTLIVTLMLHSPVALQKQLSDAV 118
HsCSE1L           IKVCASVTFKNYIKRNWRIVEDE--PNKICEADRVAIKANIVHLMLSSPEQIQKQLSDAI 115
                   ::..::::**::****    :     .: : *.  ::: ::  *: :   :*: **:*:

CeXPO-2           YLIAQRDFPEKWPDLVPYLSKFLNGADLNHLVASLASMEQIFRKFRFESKSAELWKELKK 169
DmCAS-PA          SIIGKYDFPKKWPQLIDEMVERFASGDFNVINGVLQTAHSLFKRYRYEFKSQALWEEIKF 178
HsCSE1L           SIIGREDFPQKWPDLLTEMVNRFQSGDFHVINGVLRTAHSLFKRYRHEFKSNELWTEIKL 175
                   :*.: ***:***:*:  : : : ..*:: : . * : ..:*:::*.* **  ** *:*

CeXPO-2           CLLSTQEPLTLLLRNMMEVGQRKDQLGADEIAQWLRVLLLIAKVYHSLCSQEIPEYFEDH 229
DmCAS-PA          VLDRMAKPLTDLLQATMQLTKVHENN-AGALKVIYGSLVLVNKVFFSLNSQDLPEFFEDN 237
HsCSE1L           VLDAFALPLTNLFKATIELCSTHAND-ASALRILFSSLILISKLFYSLNFQDLPEFFEDN 234
                   *     *** *::  ::: . : :  *. :      *:*: *::.**  *::**:***:

CeXPO-2           LKDWMPHFLHLVQIDAP-TQTSNSGEPTTLDELKHEICEIFTLYSQRYEEEISEFVPDII 288
DmCAS-PA          INTWMGAFIQQLAADVPSLRTADDEDAGVLEHLRAQVCENICLYAKKYDEEFKPFMEQFV 297
HsCSE1L           METWMNNFHTLLTLDNKLLQTDDEEEAGLLELLKSQICDNAALYAQKYDEEFQRYLPRFV 294
                  :: **  *   :  *    :* :. :.  *: *: ::*: **:::*:**:. ::  ::

CeXPO-2           LAVWNLLKSTGPDTRYDTMVCAALEFLSMVSQRQYYEGHFTGEGVLKTLAENVCVQNLLL 348
DmCAS-PA          TAVWELLVKTSLHTKYDSLVSHALQFLSVVADRQHYQSIFENPEILAQICDKVVIPNLDI 357
HsCSE1L           TAIWNLLVTTGQEVKYDLLVSNAIQFLASVCERPHYKNLFEDQNTLTSICEKVIVPNMEF 354
                   *:*:** .*. ..:** :*. *::**: *.:* :*:. * .   *  :.::* : *: :

CeXPO-2           RQQDMELFEDEPLDYMKRDIEGTDVGTRRRGAIDLARGLCRRFEAQMLPCLGEIVQNLLG 408
DmCAS-PA          RPSDEEIFEDSPEEYIRRDIEGSDIDTRRRAACDLVKTLSINFEQKIFGIFGQYLERLLT 417
HsCSE1L           RAADEEAFEDNSEEYIRRDLEGSDIDTRRRAACDLVRGLCKFFEGPVTGIFSGYVNSMLQ 414
                  *  * * ***.. :*::**:**:*:.****.* **.: *.  **  :   :.  :: :*
```

```
CeXPO-2         SG------DWIKIDIVYSLITAIAVKTETAKSGVTATNPLVDINDFFITQVATHLN-ADV 461
DmCAS-PA        KYKENPATNWRSKDTAIYLVTSWASRGGTQKHGITQTSELVPLPEFCAQQIIPELERPNI 477
HsCSE1L         EYAKNPSVNWKHKDAAIYLVTSLASKAQTQKHGITQANELVNLTEFFVNHILPDLKSANV 474
                .       :*   * .  *:*: * :  * * *:* :. ** : :*   :: ..*: .::

CeXPO-2         NQTPILKADALKFAVTFRKQLAPEHLMTAIKSADALLSSNTPILHKYAAYAIEKILLADS 521
DmCAS-PA        NEFPVLKAAAIKYVMVFRSILGPQVLASCLPQLIRHLPAESSVVHSYAACSVEKILSMRD 537
HsCSE1L         NEFPVLKADGIKYIMIFRNQVPKEHLLVSIPLLINHLQAESIVVHTYAAHALERLFTMRG 534
                *: *:*** .:*: : **. :  : *  .:      * ::: ::*.*** ::*:::   .

CeXPO-2         NK---IFSAQNLP--VSSILQNLVTAFDKDAKAQNSPYLIKAILRIIVILDDDTIRHADA 576
DmCAS-PA        ASNAIVFGPQILAPYTTELISGLFATLSLPGSGENE-YVMKAIMRSFSVLQSAAMPFMGV 596
HsCSE1L         PNNATLFTAAEIAPFVEILLTNLFKALTLPGSSENE-YIMKAIMRSFSLLQEAIIPYIPT 593
                 .   :* .  :.  .  :: .*. ::   ...:*. *::***:* : :*:.  : .  .

CeXPO-2         IAVKLAQLVESATKNPADSVHTHFLFETICVLITKTRTIG----ASLDAQLLPLIEVIFR 632
DmCAS-PA        ALPRLTEILTQVAKNPSRPQFNHYLFETLALCIKIVCHADSSAVSSFEEALFPVFQGILQ 656
HsCSE1L         LITQLTQKLLAVSKNPSKPHFNHYMFEAICLSIRITCKANPAAVVNFEEALFLVFTEILQ 653
                   :*:: :  .:***: . ..*::**::.: *  .   .     .::  *: ::  *::
                                                           ax2013 S679L
CeXPO-2         EDLEDLIPYALQITGVLVSSCIARNSSIDQFSPFLPFLLSERLWARSANVPAALSVLEVI 692
DmCAS-PA        QDIVEFMPYVFQMLSVLLEMREGTGTIPEPYWALFPCLLSPALWDRTGNVTPLIRLISAF 716
HsCSE1L         NDVQEFIPYVFQVMSLLLETHKN--DIPSSYMALFPHLLQPVLWERTGNIPALVRLLQAF 711
                :*: :::**.:*: .:*:.         . : .::* **.  ** *:.*:.. : ::..:

CeXPO-2         LSVNAQRVVS---ENSGLILSHLARLLGSKTLDQYGFQLAATILPSIEHFEG-SAMTFVL 748
DmCAS-PA        IKQGSAQIQ--ALGKLSGILGIFQKMIASKANDHEGFYLLQNLLSYYPPAEIQTNLRQIF 774
HsCSE1L         LERGSNTIASAAADKIPGLLGVFQKLIASKANDHQGFYLLNSIIEHMPPESVDQYRKQIF 771
                :. .:  :      :   :*. : :::.**: *: ** *  .::      .       ::

CeXPO-2         NTMFRRVQSSKTPKFMKLFIVFLCRFTIARSAQDLVQSCENIQTGMFGMLIEKVVCIDLP 808
DmCAS-PA        GLLFQRLSLSKTPKYLSGIIIFFSFYVIKFSGSQMAQLIDEIQPNLFGMLLDRVFITEMG 834
HsCSE1L         ILLFQRLQNSKTTKFIKSFLVFINLYCIKYGALALQEIFDGIQPKMFGMVLEKIIIPEIQ 831
                  :*:*:. ***.*::. :::*:  : *  ..  : :  : **. :***:::::.  ::

CeXPO-2         GLKQTTTGPEKRIIAIGMGNLLADVTQQLVGQYG-ILSYEVAMLLEAASASDRAVLSPEE 867
DmCAS-PA        ---KIPKEQDRKMVAVGVTKLLTETPEILQQQYATFWPRLLHSLIDLFERPPEKLMGLEI 891
HsCSE1L         ---KVSGNVEKKICAVGITKLLTECPPMMDTEYTKLWTPLLQSLIGLFELPEDDTIPDEE 888
                   : .   :::: *:*: :**:: .  :  :*  : .  :  *:   . .    :  *

CeXPO-2         EQASMYNAEG--EFVNPFCRLSYAPK--QPPVAANIANHKAYFAQAVLVRGPGNCPETLR 923
DmCAS-PA        GETAGVAEDPDAGYQVAFAQLTHAQPNQQDHLAEIKD-ARQFLATSLSKFAQARAGEFST 950
HsCSE1L         -HFIDIEDTP--GYQTAFSQLAFAGKKEHDPVGQMVNNPKIHLAQSLHKLSTACPGRVPS 945
                 .           :  .*.:*:.*    :  :.      : .:* ::   . .   .

CeXPO-2         SVPP----EIVTYLQSIQQ------- 938
DmCAS-PA        LLSP-LEPEYKQVLQKYCDQAGVRIA 975
HsCSE1L         MVSTSLNAEALQYLQGYLQAASVTLL 971
                 :..    *    **   :
```

Figure S4 Multiple alignment of suppressor genes**.** Protein sequences of suppressor genes were aligned with their *D. melanogaster* and human orthologs by ClustalW2 using Gonnet PAM 250 matrix. Amino acids mutated in *dd5* and suppressor alleles are highlighted. If amino acids are conserved, a symbol "*" (identical amino acid in all three species) or ":" (strong conservation that scores >0.5) or "." (weak conservation that scores ≤0.5) is shown below the sequence.

Table S1 Nematode strains used in this study

Strain	Genotype	Description
**TH48**	*mbk-2(dd5)*	
**EU1065**	*und-119(ed3; orIs1 III [unc-119; pie-1 promoter::GFP::MEI-1 fusion]*	
**CB4856**	Hawaiian (Hw)	
**JH2291**	*mbk-2(dd5);* Hw	N2 gaps on I (1.5-12.8Mb) and IV (0-15.8Mb)
**JH2996**	*mbk-2(dd5);cdc-37(ax2001)*	
**JH2997**	*mbk-2(dd5);plk-1(ax2002)*	
**JH2998**	*mbk-2(dd5);emb-30(ax2003)*	
**JH2999**	*mbk-2(dd5ax2004)*	
**JH3000**	*mbk-2(dd5ax2005)*	
**JH3001**	*mbk-2(dd5ax2006)*	
**JH3002**	*mbk-2(dd5ax2007)*	
**JH3003**	*mbk-2(dd5);plk-1(ax2008)*	
**JH3004**	*mbk-2(dd5);tat-4(ax2009)*	
**JH3005**	*mbk-2(dd5);such-1(ax2010)*	
**JH3006**	*mbk-2(dd5);mus-101(ax2011)*	
**JH3007**	*mbk-2(dd5);mat-1(ax2012)*	
**JH3008**	*mbk-2(dd5);xpo-2(ax2013)*	
**JH3009**	*mbk-2(dd5);fzy-1(ax2014)*	
**JH3010**	*mbk-2(dd5);unc-119(ed3); orIs1 III*	cross JH1570 with TH48
**JH3011**	*mbk-2(dd5);cdc-37(ax2001);unc-119(ed3); orIs1 III*	cross JH3010 with JH2996
**JH3012**	*mbk-2(dd5);plk-1(ax2002);unc-119(ed3); orIs1 III*	cross JH3010 with JH2997
**JH3013**	*mbk-2(dd5ax2004); unc-119(ed3); orIs1 III*	cross JH3010 with JH2999
**JH3014**	*mbk-2(dd5ax2005); unc-119(ed3); orIs1 III*	cross JH3010 with JH3000
**JH3015**	*mbk-2(dd5);plk-1(ax2008); unc-119(ed3); orIs1 III*	cross JH3010 with JH3003
**JH3075**	*mbk-2(dd5);tat-4(ax2009); unc-119(ed3); orIs1 III*	cross JH3010 with JH3004
**JH3076**	*mbk-2(dd5);such-1(ax2010); unc-119(ed3); orIs1 III*	cross JH3010 with JH3005
**JH3077**	*mbk-2(dd5);mus-101(ax2011); unc-119(ed3); orIs1 III*	cross JH3010 with JH3006
**JH3078**	*mbk-2(dd5);mat-1(ax2012); unc-119(ed3); orIs1 III*	cross JH3010 with JH3007
**JH3079**	*mbk-2(dd5);xpo-2(ax2013); unc-119(ed3); orIs1 III*	cross JH3010 with JH3008
**JH3080**	*mbk-2(dd5);fzy-1(ax2014); unc-119(ed3); orIs1 III*	cross JH3010 with JH3009
**JH3086**	*mbk-2(dd5);emb-30(ax2003); unc-119(ed3); orIs1 III*	cross JH3010 with JH2998
**JH3087**	*mbk-2(dd5);xpo-2(ax2013); unc-119(ed3); orIs1 III*	cross JH3010 with JH3008
**CB5584**	*mIs12 II [myo-2::GFP, pes-10::GFP, F22B7.9::GFP]*	strong GFP in 4-cell embryos, pharynx muscle and gut.
**JH1279**	*mIsXX IV [myo-2::GFP, pes-10::GFP, F22B7.9::GFP]*	PD4790
**JH3085**	*mbk-2(dd5);mIs12 II*	cross CB5584 with TH48
