# Supplementary material for: Identification of Suppressors of mbk-2/DYRK by Whole-Genome Sequencing
Source: G3 (Bethesda). 2013 Dec 17;4(2):231–41. doi: 10.1534/g3.113.009126 (PMC3931558; doi:10.1534/g3.113.009126)
Supplement: Supporting Information [file supp_g3.113.009126_FigureS1.pdf]

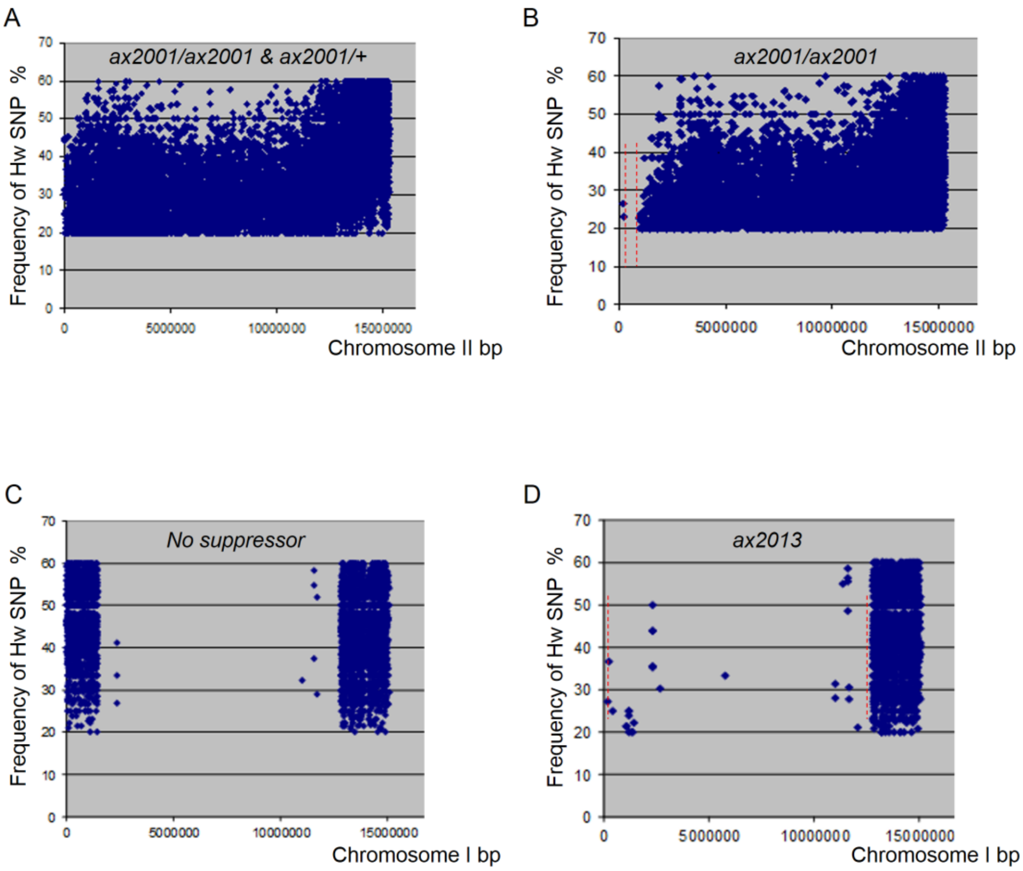

Figure S1 Mapping of *ax2001* and *ax2013*.

(A-D) Graphs showing the percentage of Hawaiian SNPs (Y axis) along the linkage group indicated (X axis). Each dot represents a unique Hawaiian SNP, only SNPs with frequencies between 20-60% are displayed.

(A) 250 progeny of F1 N2/Hw hybrids (*mbk-2(dd5)/mbk-2(dd5)*Hw; *ax2001*/Hw) were grown at 25°C for two generations on one plate and sequenced in one pool. Since ax2001 is dominant, the recombinants are a mixture of *ax2001*/Hw and *ax2001/ax2001* animals. As a result, no distinct region with low Hw SNP frequencies was detected.

(B) Same as in A, except that the recombinants were screened for homozygosity at the suppressor locus before sequencing (Methods). 26 *ax2001/ax2001* F2 recombinants were grown for 2 more generations and sequenced in one pool. A 1Mb region with low Hw SNP frequency was detected at the extreme right end of LGII.

(C) Same as in A showing linkage group I for the introgressed *mbk-2(dd5)*Hw strain. Note the large gap lacking Hw SNPs. This gap is likely due to the N2/Hw incompatibility at the peel-1/zeel-1 locus on LGI and the fact that we used Hw/N2 males to make the introgressed *mbk-2(dd5)*Hw strain .

(D) Same as in C for a population of F2 recombinants carrying suppressor *ax2013*. Note the loss of Hw SNPs denoting linkage to the right end of LGI. However because the large gap in the introgressed strain (C), the entire region demarcated by red lines was scanned for novel mutations.
