# Supplementary material for: Identification of Suppressors of mbk-2/DYRK by Whole-Genome Sequencing
Source: G3 (Bethesda). 2013 Dec 17;4(2):231–41. doi: 10.1534/g3.113.009126 (PMC3931558; doi:10.1534/g3.113.009126)
Supplement: Supporting Information [file supp_g3.113.009126_FigureS2.pdf]

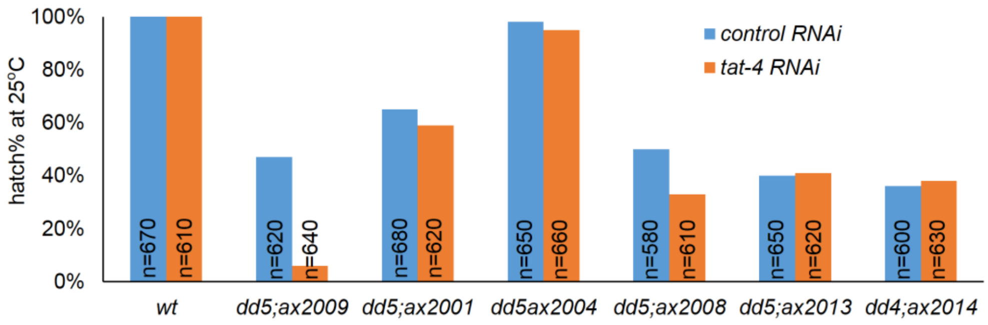

Figure S2 RNAi of *tat-4* reverses the suppression of *ax2009* but of no other suppressor. Percentage of hatched embryos among the progeny of hermaphrodites of the genotypes shown and fed with blank (control) or *tat-4* dsRNA.
