# Supplementary material for: Identification of Suppressors of mbk-2/DYRK by Whole-Genome Sequencing
Source: G3 (Bethesda). 2013 Dec 17;4(2):231–41. doi: 10.1534/g3.113.009126 (PMC3931558; doi:10.1534/g3.113.009126)
Supplement: Supporting Information [file supp_g3.113.009126_FigureS3.pdf]

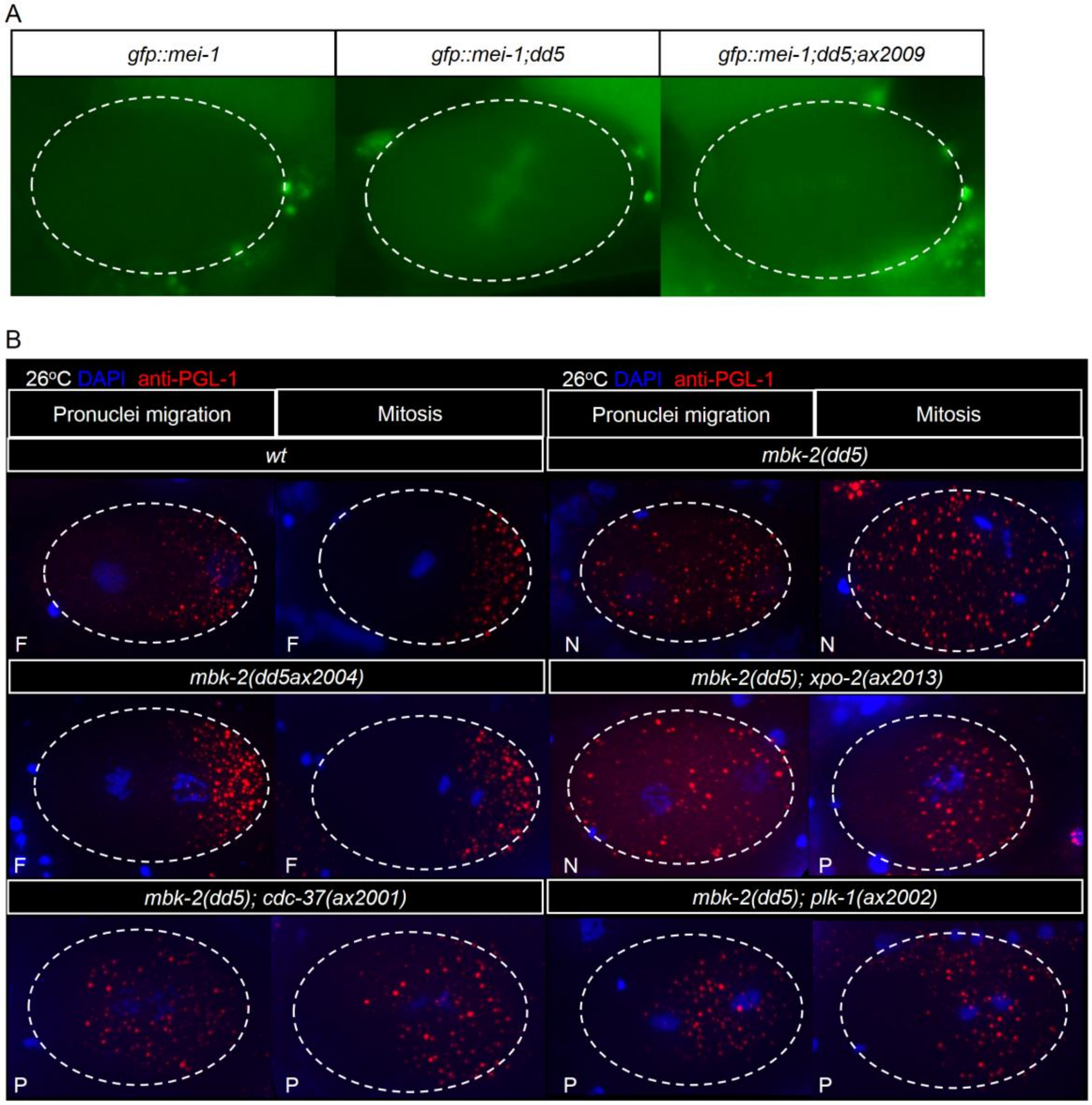

Figure S3 GFP::MEI-1 and P granules in suppressors. (A) Examples of zygotes expressing GFP::MEI-1. Note GFP::MEI-1 on the spindle in the dd5 zygotes. (B) Examples of zygotes stained with anti-PGL-1 antibody. Example of full (F), partial (P) and no segregation (N).
