# Supplementary material for: Identification of Suppressors of mbk-2/DYRK by Whole-Genome Sequencing
Source: G3 (Bethesda). 2013 Dec 17;4(2):231–41. doi: 10.1534/g3.113.009126 (PMC3931558; doi:10.1534/g3.113.009126)
Supplement: Supporting Information [file supp_g3.113.009126_FigureS4.pdf]

MBK-2

```
CeMBK-2.a       -----------MTLFEPSTSG------NRMG--------------------YRGSSNSSS 23
DmDYRK3-PE      MVGSQEKKNNHIELSETPATDKNNLNTTHLENTQLSKALSPPTSLPQIQIQMINQNLTHT 60
HsDYRK2         -----------MLTRKPSAAAPAAYPTGRGGDSAVRQLQASP---------GLGAGATRS 40
                           :   :..::       :                        . . : :

CeMBK-2.a       GVGSG------------GSGSLMTQSIGGP----NKHLSASHSTLNTAS----------- 56
DmDYRK3-PE      GIAQNNTEKANRHQYRDSGLQYLTRCFEPLAMLNDSKEDFPTQPSNNIANYPGDIQILPI 120
HsDYRK2         GVGTGPPSPIALPPLRASNAAAAAHTIGGSKHTMNDHLHVGSHAHGQIQ----------- 89
                *:. .            ..    :: :       :.:      . .

CeMBK-2.a       --THDMMHSKIPKSPSNESLSRSHTSS------------------SGGSQGGHNSNSGS- 95
DmDYRK3-PE      FDCCEISESIQAISLPNVTSPSKTKDVPGLFLRTISENSKSKSEPECESLISVKESSVME 180
HsDYRK2         --VQQLFEDNSNKRTVLTTQPNGLTTVGKTGLPVVPERQLDSIHRRQGSSTSLKSMEGMG 147
                    :: ..          : .    .                       *  . :. .

CeMBK-2.a       -------------------------NSGFRPEDAVQTFGAKLVPFEKNEIYNYTRVFFVG 130
DmDYRK3-PE      NHTFLFHEQIIMSGQQKCELHEKPKVLVVSPQQVMILYMNKLTPYERTEILTYPQIYFIG 240
HsDYRK2         ---------------------KVKATPMTPEQAMKQYMQKLTAFEHHEIFSYPEIYFLG 185
                                        . *::.:  :  **..:*: **  .*..::*:*

CeMBK-2.a       SHAKKQAGVIGGANNGGYDDENGSYQLVVHDHIAYRYEVLKVIGKGSFGQVIKAFDHKYQ 190
DmDYRK3-PE      ANAKKRPGVYG-PNNSEYDNEQGAYIHVPHDHVAYRYEMLKIIGKGSFGQVIKAYDHKTH 299
HsDYRK2         LNAKKRQGMTGGPNNGGYDDDQGSYVQVPHDHVAYRYEVLKVIGKGSFGQVVKAYDHKVH 245
                 :***: *: * .**. **:::*:*  * ***:*****:**:*********:**:*** :

CeMBK-2.a       QYVALKLVRNEKRFHRQADEEIRILDHLRRQDSDGTHNIIHMLDYFNFRNHKCITFELLS 250
DmDYRK3-PE      EHVALKIVRNEKRFHRQAQEEIRILHHLRRHDKYNTMNIIHMFDYFTFRNHTCITFELLS 359
HsDYRK2         QHVALKMVRNEKRFHRQAAEEIRILEHLRKQDKDNTMNVIHMLENFTFRNHICMTFELLS 305
                ::****:*********** ******.***::*. .* *:***:: *.**** *:******
                                            ax2005 L283F
CeMBK-2.a       INLYELIKRNKFQGFSLMLVRKFAYSMLLCLDLLQKNRLIHCDLKPENVLLKQQGRSGIK 310
DmDYRK3-PE      INLYELIKKNGFKGFSLQLVRKFAHSLLQCLDALYKNDIIHCDMKPENVLLKQQGRSGIK 419
HsDYRK2         MNLYELIKKNKFQGFSLPLVRKFAHSILQCLDALHKNRIIHCDLKPENILLKQQGRSGIK 365
                :*******:* *:**** ******:*:* *** * ** :****:****:***********
                ax2007 V311I                       ax2006 L353F
CeMBK-2.a       VIDFGSSCFDDQRIYTYIQSRFYRAPEVILGTKYGMPIDMWSLGCILAELLTGYPLLPGE 370
DmDYRK3-PE      VIDFGSSCFENQRIYTYIQSRFYRAPEVILGGKYGRAIDMWSLGCILAELLSGHALFPGE 479
HsDYRK2         VIDFGSSCYEHQRVYTYIQSRFYRAPEVILGARYGMPIDMWSLGCILAELLTGYPLLPGE 425
                ********::.**:*****************  :** .**************:*:.*:***
                 dd5 D374N
CeMBK-2.a       DENDQLALIIELLGMPPPKSLETAKRARTFITSKGYPRYCTATSMPDGSVVLAGARSKRG 430
DmDYRK3-PE      NESDQLACIIEVLGMPNKNILASSKRSKSFFSPKGYPRYCTVRTMSDGMVVLIGGQSRRG 539
HsDYRK2         DEGDQLACMIELLGMPSQKLLDASKRAKNFVSSKGYPRYCTVTTLSDGSVVLNGGRSRRG 485
                :*.**** :**:****   : * ::**::.*.:.********. ::.** *** *.:*:**
                ax2004 R433C
CeMBK-2.a       KMRGPPASRSWSTALKNMGDELFVDFLKRCLDWDPETRMTPAQALKHKWLRRRLPNPPRD 490
DmDYRK3-PE      KQRGPPCSKSLSKALDGCKDPLFLNFIRGCLEWDADKRLTPSEALKHPWLRRRLPRPPSS 599
HsDYRK2         KLRGPPESREWGNALKGCDDPLFLDFLKQCLEWDPAVRMTPGQALRHPWLRRRLPKPPTG 545
                * **** *:. ..**..  * **::*:: **:**.  *:**.:**:* *******.** .

CeMBK-2.a       ---------------------GLESMGGLADHEVCFIIF-------------------- 508
DmDYRK3-PE      SSGCGGVSGLCSSRNESPVTGQNRNFAAETTASSTSATSISLTIKRENSHSSLRLHHGAV 659
HsDYRK2         ---------------------EKTSVKRITESTGAITSISKLPPPSSSASKLRTNLAQM 583
                                        :    .   :
```

CDC-37

```
CeCDC-37.a       MPIDYSKWKDIEVSDDEDDTHPNIDTPSLFRWRHQARLERMAEKKMEQEKIDKEKGTTSK 60
DmCDC-37-PA      -MVDYSKWKNIEISDDEDDTHPNIDTPSLFRWRHQARVERMAEMDHEKDELKKKRQSYQA 59
HsCDC-37         -MVDYSVWDHIEVSDDEDETHPNIDTASLFRWRHQARVERMEQFQKEKEELDRGCRECKR 59
                   :*** *..**:*****:*******.**********:***  : . *::::.:     .

CeCDC-37.a       KMEELEKKLAAADVTDKSDIQKQIDEVKAQEEAWRKKEA-------ELEEKERLEPWNVD 113
DmCDC-37-PA      RLMDVKERISKKDGDEE-ALKKELEKIEAEGKELDRIES-------EMIKKEKKTPWNVD 111
HsCDC-37         KVAECQRKLKELEVAE--GGKAELERLQAEAQQLRKEERSWEQKLEEMRKKEKSMPWNVD 117
                 :: : :.::   :  :    : :::.::*: :   : *         *: :**:  *****
CeCDC-37.a       TIGHEAFSTSRINKITEKKPQAPKTDEEDTHAMSTFFETHESLLEKMAVLKNGAKSTELF 173
DmCDC-37-PA      TISKPGFEKTVINKKAGRKPDENLSEEEREQRMKQFVKENEKLCQQYGMLRKYDDS-KRF 170
HsCDC-37         TLSKDGFSKSMVNTKPEKTEED--SEEVREQKHKTFVEKYEKQIKHFGMLRRWDDS-QKY 174
                 *:.: .*..: :*. . :. :    ::*    :  . *.:  *.  :: .:*:.  .* : :
```

```
                                                       ax2001 L221F
CeCDC-37.a     LAEHPHMASEYTANWLTIEALNAAIDFNEEKMKTMAEQCIIIQYLLELSKSLNAVATNTT 233
DmCDC-37-PA    LQEHLHLVGEETANYLVIWSINLEMEEKHELMAHVAHQCICMQYILELAKQLDVDPR--A 228
HsCDC-37       LSDNVHLVCEETANYLVIWCIDLEVEEKCALMEQVAHQTIVMQFILELAKSLKVDPR--A 232
               * :: *:. * ***:*.* .::  :: :   *  :*.* * :*::***:*.*.. .   :

CeCDC-37.a     VQKQFFKKFEAAEPVYMKHYQDEVKAFEDRLRTRAQTKRDAAMEEAEAEEKAERMKSAPG 293
DmCDC-37-PA    CVSSFFSKIQHCHPEYRAQFDSEIEGFKGRIQKRAQEKIQEAIAQAEEEERKERLG--PG 286
HsCDC-37       CFRQFFTKIKTADRQYMEGFNDELEAFKERVRGRAKLRIEKAMKEYEEEERKKRLG--PG 290
                  .**.*:: ..  *   ::.*::.*: *:: **: : : *: : * **: :*:    **

CeCDC-37.a     GIDPQEVFEQLPEEMRKCFEAHDIEALKGVAQKMDEEVFKYHFDRCIASGLWVPG-KADD 352
DmCDC-37-PA    GLDPADVFESLPDELKACFESRDVELLQKTIAAMPVDVAKLHMKRCVDSGLWVPNAADLE 346
HsCDC-37       GLDPVEVYESLPEELQKCFDVKDVQMLQDAISKMDPTDAKYHMQRCIDSGLWVPNSKASE 350
               *:** :*:*.**:*:: **: :*:: *: .    *     * *:.**: ******.    :

CeCDC-37.a     DDDDEEAAPAE----------EEPTTSS---------------- 370
DmCDC-37-PA    GDKKEEDDSDD-VAGGEEKTDDAKSESAAKEEPIYTGVSTEDVD 389
HsCDC-37       AKEGEEAGPGDPLLEAVPKTGDEKDVSV---------------- 378
                .. **  . :          :    *
```

TAT-4

```
CeTAT-4.a      -------------------------------------------------MTTPR------- 5
DmCG33298-PB   MPSANTEDLRRKFLVVQQQRSAPPNLTGDTLSSGIPNASASSATTSLMMTTSTGLGGVAM 60
HsATP10B       -----------------------------------------------MALSVDS------- 7
                                                                 ::.

CeTAT-4.a      ------------------------------------PTHLHRRSSSK------------- 16
DmCG33298-PB   GAGEPERTYVFPGGNPVVGSAPGGGGQGIPGVAMAAPSRGHARSISHGGGAIVGANGRPI 120
HsATP10B       ------------------------------------SWHRWQWRVRDG------------ 19
                                                    . :       .

CeTAT-4.a      ----------------------------------------WVPP---SAPLHNP-----I 28
DmCG33298-PB   KSAMKGHQRAFSQGQITDSPPGSAAPAGRGHSRVGSKTDFILPPGHKEEPAREPSAPTSA 180
HsATP10B       ----------------------------------------FPHCPSETTPLLSP------ 33
                                                                *  .*

CeTAT-4.a      SIFRGRSNRETGSRVIRPNHLFESP---------------RYELNNYR------------ 61
DmCG33298-PB   TGGRGHSRQASRSESIYTLRRTEAPPWWKRLTLCNYNTGDKFEERSYRTVVPNHTVPPKT 240
HsATP10B       -EKGRQSYNLTQQRVVFPNNSIFHQ---------------DWEEVSRR------------ 65
                    :* . : .. : . .                     :*  . *

CeTAT-4.a      --------DFTDNRISTTKYTFWNFIPLNIWHQVSTKYANLYFIFIAILNWVPFFDAYTR 113
DmCG33298-PB   PKRDHPNGQFVGNKIRTTKYTLLSFIPKNLLEQFH-RVANLYFIFIVLLNWVPEISAFGK 299
HsATP10B       ---------YPGNRTCTTKYTLFTFLPRNLFEQFH-RWANLYFLFLVILNWMPSMEVFHR 115
                         : .*:  *****: .*:* *: .*.  : *****:*:.:***:* :..: :

CeTAT-4.a      YVGLIPICFVLGTTLIKDGIEDYRRYKFDNQINKKTCHVWDRDRCAFRKTEWRYILVGDF 173
DmCG33298-PB   EVAMIPVLFVLGVTAVKDLFEDRRRRASDKRINNTTCRVYDGETERYKKVKWQELRVGDI 359
HsATP10B       EITMLPLAIVLFVIMIKDGMEDFKRHRFDKAINCSNIRIYERKEQTYVQKCWKDVRVGDF 175
                : ::*: :** .  :** :** :*   *: ** .. :::: .   : :  *: : ***:

CeTAT-4.a      VHISNNQDVPADIIILLRSSSESGTCYIETCNLDGETSLKQRMVPAKVIDYSKKDSTFKPP 233
DmCG33298-PB   VHLSNNETVPADILLLRTSDPQGVCYIDTCDLDGETNLKRREV---VRGFEEMQSIFVPS 416
HsATP10B       IQMKCNEIVPADILLLFSSDPNGICHLETASLDGETNLKQRCV---VKGFSQQEVQFEPE 232
               :::. *: *****:** :*. .* *:::*..*****.**:* *   * .:.: :  * *

CeTAT-4.a      DFTGVVTCEKPDKSIYTIRAKVEFEPGQSDVIIKENMLLRGSRIKNTTFVEGIVVYAGHD 293
DmCG33298-PB   KFVSRVEADAPTTKLYRFHGALIHPTGERVPISTECLLLRESRLKNTDYIEGIVVYAGHE 476
HsATP10B       LFHNTIVCEKPNNHLNKFKGYMEHPDQTRTGFGCESLLLRGCTIRNTEMAVGIVIYAGHE 292
                * . : .: * . :  ::. : .        :  * :*** . ::**    ***:****:

CeTAT-4.a      TKVMQNNGRAPHKTSGIEKLTNKFIIACFIMLLLMVLYGAITSAVWVGQHPVDEQIPFIL 353
DmCG33298-PB   TKSMLNNSGPRYKRSQVEQQMNIDVIWCVIILLILCVVGAIGCRMWLSSF-THFPVPYLP 535
HsATP10B       TKAMLNNSGPRYKRSKIERRMNIDIFFCIGILILMCLIGAVGHSIWNGTFEEHPPFDVPD 352
               ** * **. . :* * :*:  *  :: *. :*::: : **:   :* .. . .  .  .
```

```
CeTAT-4.a        SN--TPRPFIEGFIGIGAFFINYQLLVPISLYITVEIIKALQIYFISNDIQLYDQKSDRA 411
DmCG33298-PB     PN--KLTANMESMWIFWTYIVILQVMIPLSLYVTIELCKILQVFHIHNNVDLFDAETNKQ 593
HsATP10B         ANGSFLPSALGGFYMFLTMIILLQVLIPISLYVSIELVKLGQVFFLSNDLDLYDEETDLS 412
                 .*     . : .:  : : ::  *:::*:***:::*: *  *::.: *:::*:* :::

CeTAT-4.a        IDCRSLSIPEELGTVTHVLSDKTGTLTENMMIFRNCAFDETDYGSNGS-----QSNPDKP 466
DmCG33298-PB     TECRAMNITEELGQIQHIFTDKTGTLTENKMIFRRCVVNGSDYNHPPSELEKIYSKPGAP 653
HsATP10B         IQCRALNIAEDLGQIQYIFSDKTGTLTENKMVFRRCTIMGSEYSHQENAKRLETPKELDS 472
                  :**::.*.*:** : ::::********* *:**.*..  ::*.   .      .:   .

CeTAT-4.a        VKSDELYQRILTSMQNPVQ-----------KHFFANILLNNSVVVNHIPHTDVLELGNFD 515
DmCG33298-PB     APPLIPNDNLNSDMAQLTQGTYLTPHAQRIQEFLVVLAICNTVIVGAAPHRDMMNASGII 713
HsATP10B         DGEEWTQYQCLSFSARWAQDP----ATMRSQKGAQPLRRSQSARVPIQGHYRQRSMGHRE 528
                          .  :   . .*           :.    :   ::. *    *    . .
                                        ax2008 G544S
CeTAT-4.a        GGVYNIGNSCFYDVTEEKYKQKLAAIGKGGVDDDDVSRP-DELGLPTTSIQFDDRLTVIV 574
DmCG33298-PB     E-VQQIGNS-PANLKHGKQRQKLLASSTSTTTTTTIING-PTTQPQVVSIP-ADRYIRLA 769
HsATP10B         SSQPPVAFSSSIEKDVTPDKNLLTKVRDAALWLETLSDSRPAKASLSTTSSIADFFLALT 588
                      :. *   :      :: *     .       :           .:    *    :.

CeTAT-4.a        EEDTPSDSPAP-----------------------------------DASPRDLPETPTP- 598
DmCG33298-PB     ESRSVTPSPPPNLLFALPAQSHQPTL-------------SPISSSAESSPNSESESPSPP 816
HsATP10B         ICNSVMVSTTTEPRQRVTIKPSSKALGTSLEKIQQLFQKLKLLSLSQSFSSTAPSDTDLG 648
                    :   *...                                   :: .   .. .

CeTAT-4.a        ---------TSPIYRPLSSLSSFSRKLSTVVRRSILRPISDIIPVRKRLISFKQQAMNP- 648
DmCG33298-PB     MKNKSLSNSISPTGRAKAVINSKITSIATFLNAKTQGKRMKLPSSKTGTIYRTADGRPL- 875
HsATP10B         ESLGANVATTDSDERDDASVCSGGDSTDDGGYRSSMWDQGDILESGSGTSLEEALEAPAT 708
                               ..  *  : : *    .        .       .:    .

CeTAT-4.a        --------YEAESPDELALIEGAALYDYVLLERAATSVTISTPEK-AEKRYEILLTLPFD 699
DmCG33298-PB     --------YEAESPDELALVNAAYSYDCCLLNRSPNQILVSMPMAGATREYEILKVLPFD 927
HsATP10B         DLARPEFCYEAESPDEAALVHAAHAYSFTLVSRTPEQVTVRLPQG-TCLTFSLLCTLGFD 767
                         ******** **:..*  *.  *:.*:. .: :  *   :   :.:* .* **

CeTAT-4.a        ATRKRMSVIVNSQKGP--LMYCKGADSAIISRLS---------SDSLESKRVQDLKDHLD 748
DmCG33298-PB     SSRKCMSIVVRQIGSQEIVLYTKGADSSIMPVLVP------CSHNSPEGILREQTQQLLD 981
HsATP10B         SVRKRMSVVVRHPLTGEIVVYTKGADSVIMDLLEDPACVPDINMEKKLRKIRARTQKHLD 827
                 : ** **::*.        ::* ***** *:  *             :.          :. **

CeTAT-4.a        NYAKKGLRTLCFAMKYISKEDFEDFLDSYRFLMEDATSEREKMLSEKADELETNLKLSGV 808
DmCG33298-PB     RYAREGLRILVMAKRTLNSADYTDWWARH-QEIEMSLENRERRLRDSFAKLESNLTLLGA 1040
HsATP10B         LYARDGLRTLCIAKKVVSEEDFR-RWASFRREAEASLDNRDELLMETAQHLENQLTLLGA 886
                  **:.*** * :* : :.. *:       .     * : .:*:. * :.  .**.:*.* *.

CeTAT-4.a        TGIEDRLQDGVPDTLRALRDAGIQVWVLTGDKLETAQNIATSSGLFHPQRSLKVIET--- 865
DmCG33298-PB     TGIEDRLQDGVPETIASLLSAGISVWVLTGDKPETAINIAYSAKLFTQQMELIRLTARSR 1100
HsATP10B         TGIEDRLQEGVPDTIATLREAGIQLWVLTGDKQETAVNIAHSCRLLNQTDTVYTINTENQ 946
                 ********:***:*: :* .***.:******* *** *** *. *:      :  : :

CeTAT-4.a        ------------ETDAEEASESAG--------------LNIIMSPAAIRLAQDGN----- 894
DmCG33298-PB     DAAETAINFYLTDMENDKTTSTLG--------------YGQSLRKKQRALVVDGKTLTFI 1146
HsATP10B         ETCESILNCALEELKQFRELQKPDRKLFGFRLPSKTPSITSEAVVPEAGLVIDGKTLNAI 1006
                               : .  .  .. .                          *. **:

CeTAT-4.a        --------AHLMEALKKAKTVLCYRMTPSEKATIVNTVKKRIKGNVLAIGDGANDVPMIQ 946
DmCG33298-PB     LDPKSKLILPFLRLSKRCASVLCCRSTPLQKAYLVKVVKEELNLRTLAIGDGANDVSMIQ 1206
HsATP10B         FQG--KLEKKFLELTQYCRSVLCCRSTPLQKSMIVKLVRDKLRVMTLSIGDGANDVSMIQ 1064
                               ::.  : . :*** * ** :*: :*: *:..:.  .*:********.***

CeTAT-4.a        AAHVGIGIAGKEGLQAAMACDFAIARFKFLSRLLLVHGHWSYYRLANTFLYFLYKNANAV 1006
DmCG33298-PB     MADVGVGISGQEGMQAVMAADFTLPRFRYLERLLLAHGYWCYDRLSRMILYFFYKNAAFV 1266
HsATP10B         AADIGIGISGQEGMQAVMSSDFAITRFKHLKKLLLVHGHWCYSRLARMVVYYLYKNVCYV 1124
                  *.:*:**:*:**:**.*:.**::.**:.*.:***.**:*.* **:. .:*::***.  *
CeTAT-4.a        FIIFYYQFYNGASGTNIVDPIWGVIYPIIFTSVQPVVVGVLDQDYDDQTLMNKPELYVIG 1066
DmCG33298-PB     FLIFWYQLYCGFSGQVMMDQMYLMLYNLIFTSLPPLAIGVYDKRVAEDLLLKNPYLYKNG 1326
HsATP10B         NLLFWYQFFCGFSSSTMIDYWQMIFFNLFFTSLPPLVFGVLDKDISAETLLALPELYKSG 1184
                  ::*:**:: * *.  ::*    ::: ::***: *:..** *:    : *:  * **  *
```

```
CeTAT-4.a         RENQLYTWKHFFRDVIDGIYQAAVIYYVAYLTLDNSTSSLWEMGFYIATSSILVNSGHLA 1126
DmCG33298-PB      RLGVAYRPHDFWLILLDALYQSLVIFFVALCAYAESDVGIWEFGTTITASCLFANLVHCA 1386
HsATP10B          QNSECYNLSTFWISMVDAFYQSLICFFIPYLAYKGSDIDVFTFGTPINTISLTTILLHQA 1244
                  : .  *    *:  ::*.:**: : :::.  :   *  .:: :*  * : .: .   * *

CeTAT-4.a         LQVRYW-----------------------------------------------HWQLVAL 1139
DmCG33298-PB      IEIRSWTVLHVLSIVLSLGSFYLFAIVYDSVCMNCFGVRSSYWVIFVCFASAVHWLVIML 1446
HsATP10B          MEMKTWTIFHGVVLLGSFLMYFLVSLLYNATCVICNSPTNPYWVMEGQLSNPTFYLVCFL 1304
                  :::: *                                               .: :  *
```

PLK-1

```
CePLK-1.a       ---MNRLPNIAKPPQKSNQR------------KEKAPPEVPALIADKDRGTYYEKGRFLG 45
DmPOLO-PB       ---------MAAKPEDK-------------------STDIPDRLVDINQRKTYKRMRFFG 32
HsPLK1          MSAAVTAGKLARAPADPGKAGVPGVAAPGAPAAAPPAKEIPEVLVDPRSRRRYVRGRFLG 60
                         :*  * .                    . ::*  :.*      * : **:*
                                          ax2008 L74F   ax2002 V87M
CePLK-1.a       KGGFAHCYELTNRATREVVAGKVVPKSMLVKQYQRDKMTQEVQIHRELGHINIVKLFNFF 105
DmPOLO-PB       KGGFAKCYEIIDVETDDVFAGKIVSKKLMIKHNQKEKTAQEITIHRSLNHPNIVKFHNYF 92
HsPLK1          KGGFAKCFEISDADTKEVFAGKIVPKSLLLKPHQREKMSMEISIHRSLAHQHVVGFHGFF 120
                *****:*:*: :  * :*.***:*.*.:::*  *::* : *: ***.* * ::* :..:*

CePLK-1.a       EDNLNVYITLELCARRSLMELHKRRKAVTEPEARYFTHQIVDGVLYLHDLNIIHRDMKLG 165
DmPOLO-PB       EDSQNIYIVLELCKKRSMMELHKRRKSITEFECRYYIYQIIQGVKYLHDNRIIHRDLKLG 152
HsPLK1          EDNDFVFVVLELCRRRSLLELHKRRKALTEPEARYYLRQIVLGCQYLHRNRVIHRDLKLG 180
                **.  :::.**** :**::*******::** *.**:  **: *  ***  .:****:***

CePLK-1.a       NLFLNDDLVVKIGDFGLATTVNGD-ERKKTLCGTPNYIAPEVLNKAGHSFEVDIWAVGCI 224
DmPOLO-PB       NLFLNDLLHVKIGDFGLATRIEYEGERKKTLCGTPNYIAPEILTKKGHSFEVDIWSIGCV 212
HsPLK1          NLFLNEDLEVKIGDFGLATKVEYDGERKKTLCGTPNYIAPEVLSKKGHSFEVDVWSIGCI 240
                *****: * ********** :: : ****************:*.* *******:*::**:

CePLK-1.a       LYILLFGQPPFESKSLEETYSRIRHNNYTIPSIATQPAASLIRKMLDPEPTRRPTAKQVQ 284
DmPOLO-PB       MYTLLVGQPPFETKTLKDTYSKIKKCEYRVPSYLRKPAADMVIAMLQPNPESRPAIGQLL 272
HsPLK1          MYTLLVGKPPFETSCLKETYLRIKKNEYSIPKHINPVAASLIQKMLQTDPTARPTINELL 300
                :* **.*:****:. *::** :*:: :* :*.     **.::  **:.:*  **:  ::

CePLK-1.a       RDGFFKSGFMPTRLPVSCLTMVPKFGGHETSMMEENVAPRGVDARSQRPLNGRAGLSALP 344
DmPOLO-PB       NFEFLKGSKVPMFLPSSCLTMAPRIG----------SNDTIEDSMHRKPLMEMNGIRPDD 322
HsPLK1          NDEFFTSGYIPARLPITCLTIPPRFS----------IAPSSLDPSNRKPLTVLN------ 344
                .  *:.. :*  ** :***: *::.                *.  ::**

CePLK-1.a       QHIVSNNADR---ERAQQQAAEATFREPEDAYLSQLFHQVAVLLEQRIPGLEEEEAALDG 401
DmPOLO-PB       TRLESTFLKANLHDAITASAQVCRHSEDYRSDIESLYQQLTNLINGKPR--ILQGNLGDE 380
HsPLK1          KGLENPLPER---PREKEEPVVRETGEVVDCHLSDMLQQLHSVNASKPS--ERGLVRQEE 399
                  :  .         ..      *   . :..: :*:  :   :           :

CePLK-1.a       YQSPECLPVFWISKWVDYSDKYGIGYQLCDNSVGVLFNDNSRIMLDQAGNELTYIEKSNK 461
DmPOLO-PB       NTDPAAQPLFWISKWVDYSDKYGFGYQLCDEGIGVMFNDTTKLILLPNQINVHFIDKDGK 440
HsPLK1          AEDPACIPIFWVSKWVDYSDKYGLGYQLCDNSVGVLFNDSTRLILYNDGDSLQYIERDGT 459
                  .* . *:**:***********:******:.:**:***.::::*     .: :*::...

CePLK-1.a       EHYFSMHSGEMPGLLNKKVTLLKYFRSYMNDHLVKAGEGSEQRAGDDLARLPTLRVWFRT 521
DmPOLO-PB       ETYMTTTD--YCKSLDKKMKLLSYFKRYMIEHLVKAGANNVNIESDQISRMPHLHSWFRT 498
HsPLK1          ESYLTVSS--HPNSLMKKITLLKYFRNYMSEHLLKAGANITPREGDELARLPYLRTWFRT 517
                * *::  .       * **:.**.**: ** :**:*** .     .*:::*:* *: ****

CePLK-1.a       KSAIVLHLSNGTVQINFFNDHVKMMMCPLMQAVTFIDQNKRMLTYKLNNLQRNGCPEKFL 581
DmPOLO-PB       TCAVVMHLTNGSVQLN-FSDHMKLILCPRMSAITYMDQEKNFRTYRFSTIVENGVSKDLY 557
HsPLK1          RSAIILHLSNGSVQINFFQDHTKLILCPLMAAVTYIDEKRDFRTYRLSLLEEYGCCKELA 577
                 .*:::**:**:**:* *.** *:::** * *:*::*::::: **::. : . *  :.:

CePLK-1.a       HRLKYAKTMIERLMSDANVVSQNPARQPDMPRSMAAARSASAGSRGPNQAASHLPQSASG 641
DmPOLO-PB       QKIRYAQEKLRKMLEKMFT----------------------------------------- 576
HsPLK1          SRLRYARTMVDKLLSSRSASNRLKAS---------------------------------- 603
                 :::**:  : :::..  .
```

FZY-1

```
CeFZY-1         ---------------MNNKGR--TPGSAGRTVRSSAQQNGLTMR-------KRDMTPTRN 36
DmFZY-PA        MSQFNFVSDLQNALIMDGETR-GPAPRWKKKLEASLNGSVNTTRSVLSVSYNTSFSGVQA 59
HsCDC20         MAQFAFESDLHSLLQLDAPIPNAPPARWQRKAKEAAGPAPSPMR-----AANRSHSAGRT 55
                               ::       ..    :. . :      . *       : . :  :

CeFZY-1         TNLLPNATFVGDRFLGVRLDQDELDHANHLMTSKLYSNKENLNNSMSEP----------- 85
DmFZY-PA        PTKTPGKSSEGKTKKSNTTPSKTPGGGDRFIPNRAATNFELAHFLVNKDSGDKSDEENDK 119
HsCDC20         PGRTPGKSSS----KVQTTPSKP--GGDRYIPHRSAAQMEVASFLLSKE----------- 98
                .   *. :             ..    .:: :. :  :: *     :.:

CeFZY-1         ----NSPEKKSVEGEALKQMMRHKSTGALTDADDGDRILCYKKNLAPPPAIGYINQAKVL 141
DmFZY-PA        ATSSNSNESNVQASAHKGDRQKLISEVAQVGDSKGGRILCYQNKAPAAPET-HNNPLKVV 178
HsCDC20         ------NQPENSQTPTKKEHQKAWALNLNGFDVEEAKILRLSGKPQNAPEG-YQNRLKVL 151
                       : :         :  :  :         .  :**  . :   .*   : *  **:

CeFZY-1         YSTNSVINPASSVKKSTRHVKETATKVLDGPGLTKDLYSRHLDWGCHNWVAVALGHELYL 201
DmFZY-PA        YSIK----TPISTKSGSRYIPTTSERILDAPDFINDYYLNLMDWSADNIVAVALGSCVYL 234
HsCDC20         YSQKA---TPGSSRKTCRYIPSLPDRILDAPEIRNDYYLNLVDWSSGNVLAVALDNSVYL 208
                ** :    .. * :.  *::   . ::**.* : :* * . :**.. * :****.  :**

CeFZY-1         WNTETCVIKNLFEDNAPTNEGLITSVRWSQEGRYISLGYASGAVKIYDPNRPKTTEYVRE 261
DmFZY-PA        WNAQTGNIEQLTEFEE---GDYAGSLSWIQEGQILAIGNSTGAVELWDCSK------VKR 285
HsCDC20         WSASSGDILQLLQMEQP--GEYISSVAWIKEGNYLAVGTSSAEVQLWDVQQ------QKR 260
                *.:.:  * :* : :         *: * :**. :::* ::. *:::* .:       :.

CeFZY-1         LRTLRVGGASRCASIAWRKQGVMTCGYKSGDIVNHDVRISQHVVSSWGGDNGHCRDVTAL 321
DmFZY-PA        LRVMDG-HSARVGSLAWN-SFLVSSGSRDGTIVHHDVRAREHKLSTLSG---HTQEVCGL 340
HsCDC20         LRNMTS-HSARVGSLSWN-SYILSSGSRSGHIHHHDVRVAEHHVATLSG---HSQEVCGL 315
                ** :    ::* .*::*. . :::.* :.* * :****  :* ::: .*   * ::* .*

CeFZY-1         EWSADENMCVSGSSDRTAKIWDGRHVRGSTVIQDPEPMFTIDEHTGQVRTAQFCSFRDGI 381
DmFZY-PA        KWSTDFKYLASGGNDNLVNVWS---AASGGVGTATDPLHKFNDHQAAVRALAWCPWQPST 397
HsCDC20         RWAPDGRHLASGGNDNLVNVWP---SAPGEGG--WVPLQTFTQHQGAVKAVAWCPWQSNV 370
                .*:.* .  .**..*. .::*       .       *: .: :* . *::  :*.:: .
                                                                 ax2014 D434N
CeFZY-1         LATGGGINDGTVKLWDVKRQFQKVRELNVCETGGVGGIVFNRPYSEMLTAS--DDGFLRI 439
DmFZY-PA        LASGGGTADRCIKFWNVN---NGTLMKSVDSKSQVCSLLFSRHYKELISAHGFANNQLTI 454
HsCDC20         LATGGGTSDRHIRIWNVC---SGACLSAVDAHSQVCSILWSPHYKELISGHGFAQNQLVI 427
                **:***  *  :::*:*    . .     *    . * .:::.  *.*:::.    :. * *

CeFZY-1         YRFNANYKLSHEIQASNEPIMDLVGSPFDEVLIGDMEETLKVFQLFNVDKSTNILDRTAP 499
DmFZY-PA        WKYPTMVKQADLTGHTSRVLQMAMSPDGSTVISAGADETLRLWNCFAPDPLASKKAVSTS 514
HsCDC20         WKYPTMAKVAELKGHTSRVLSLTMSPDGATVASAAADETLRLWRCFELDPARRREREKAS 487
                ::: :  * :.    :.. :   :..    *  .  :***:::. *  *        .:.

CeFZY-1         KNVGLNVR---- 507
DmFZY-PA        KGKQSVFRQSIR 526
HsCDC20         AAKSSLIHQGIR 499
                      .:
```

SUCH-1

```
                             ax2010 L17F
CeSUCH-1        -MPPKKAQTRRIVSLDSLFGHTLLNITGEPVTP--------------TKIAIFQLIRTLF 45
DmIDA           MILCLHFAGKSKIGFRGPKLTMNLFEELDTLDPSFKLEPPRIETPTAHKITVLILLKQYV 60
HsANAPC5        -MASVHESLYFNPMMTNGVVHANVFGIKDWVTP--------------YKIAVLVLLNEMS 45
                 :   :         : .      :    : : *              **::: *:.

CeSUCH-1        HAHFGVGAVPSLKPFDKDEKTRVFTVLYGLIIMKSEISYDDFRCIVRILNDGLGRSIYYR 105
DmIDA           INKKNC---LDTGISMRTQRRRMFYMLVFKLIQEQDKSYNELHSLLTTGKYKLDTLMLES 117
HsANAPC5        RTG-------EGAVSLMERRR--LNQLLLPLLQGPDITLSKLYKLIEESCPQLAN----S 92
                          .      .:   :  *   ::   : : ..:  ::       *
CeSUCH-1        FVTSMEKLAHGEDQIEMLFEDAFYTAKRPNHEKVLKRE--DSWLDELTFMNSNSFLYIWI 163
DmIDA           FEKAMSEFCAGSIEALFDFSEIQNIDEILNENYGISQFSMVGVYVRRVGVVLERLSFPEM 177
HsANAPC5        VQIRIKLMAEGELKDMEQFFDDLS-DSFSGTEPEVHKTSVVGLFLRHMILAYSKLSFSQV 151
                .   :. :. *. :    * :      .  . :  : :     .    .    :  . : :  :
```

```
CeSUCH-1        KRVMMQYTRTSQNGTFEIAEKFQKWIISG--------------------TLEIISHPILS 203
DmIDA           MDMYKNVCSYYERGVRAAASGSRMAVAGGILHREETPPPNPITEKPEDPKPKKKVEAVAR 237
HsANAPC5        FKLYTALQQYFQNGEKKTVEDADMELTS----RDEG-----------ERKMEKEELDVSV 196
                  :        :.*    ..     : .                     . :     :

CeSUCH-1        GINRALPTEIDCSIRARHWCAAQLRLVQLCPTKAMSYFQILDWCDTIHRRHHDVVDVHLL 263
DmIDA           IAQERNPLSKWAPKQAKFFINKQSELLENNERKALPPLELQKKVQEIIHDLPLTTTPYFL 297
HsANAPC5        REEEVSCSGPLSQKQAEFFLSQQASLLKNDETKALTPASLQKELNNLLKFNPDFAEAHYL 256
                  :.       .  :*..:   *  *::    **:.  .: .  : : :     .  : *

CeSUCH-1        RAAIFVQLKNSSDAVKSLKQFFDMSMLEITENSKHALETLKLMSPSQVALRFGPILQGRV 323
DmIDA           GYMNQLRVRDYYNALSALHRALDRSPVR----LMSQEKGY----------QYFCVNLAVL 343
HsANAPC5        SYLNNLRVQDVFSSTHSLLHYFDRLILTGAESKSNGEEGYGRS------LRYAALNLAAL 310
                     :::::  .:  :* : :*   :          :            ::  :  . :

CeSUCH-1        HRIFGERQIASALFAESIQQSQVNVDDMCNRIANMEVTINSIYMSGPLLQRLSGETFAAG 383
DmIDA           HATFGHRDEALAALRESIMLAQEHGDKRS-----LNLANTWYCLLRDELPLSA------- 391
HsANAPC5        HCRFGHYQQAELALQEAIRIAQESNDHVC-----LQHCLSWLYVLG-------------- 351
                *  **. : *   : *:*  :*   *. .     ::   .   :

CeSUCH-1        KKEEESVENERRVQQNSVHAAVDLNVPTLRSLQKNFREDYELHAFLVSMCKFLLCIQDMM 443
DmIDA           -------------VQKSVQDANEVDGSLLQNYTLALHFAVKLGTVAGYQPLRLFDLLQHS 438
HsANAPC5        ------------------QKRSDSYVLLEHSVKKAVHFGLPR-AFAGKTANKLMDALKDS 392
                                  :   :      :.    .:      :.       *:   .

CeSUCH-1        DGKFFKHNSTADYVSVGFHRLRLLLDMNNKGFVLQAFANAIMTSGLIQSGMYHQAKRVAE 503
DmIDA           DNLTNRN-NFADHASEALALRSAVWSAYGRHELAALYSQVLLTG---------------- 481
HsANAPC5        DLLHWKH-SLSELIDISIAQKTAIWRLYGRSTMALQQAQMLLSMNSLEA----------- 440
                *    :: . ::  . .:     :    .:  :    :: :::

CeSUCH-1        TMIVSNCDAPNSPILETESHAVAGVNLVYSLAAVGDYEKAQKTIDILKNRFPENINWMAA 563
DmIDA           ---------RDRFGSGSAGLGSALASFALWLQLQGEPQLSKVLLHHAKQRFP---RLPSA 529
HsANAPC5        -------VNAGVQQNNTESFAVALCHLAELHAEQGCFAAASEVLKHLKERFPP--NSQHA 491
                          .     : . . *   :.      *    :.  :.  *:***   .   *

CeSUCH-1        RHVDICSKIVNFERNFLLNKYSECSRHLAGLETSAPLEFVLRKSLLLAATGKLAEAVLLL 623
DmIDA           EGWMISQCHVVIQAGIYQCRWHDALKACDQLYLIDPSDSLFQRASIYVAKREFFNARRLL 589
HsANAPC5        QLWMLCDQKIQFDRAMNDGKYHLADSLVTGITALNSIEGVYRKAVVLQAQNQMSEAHKLL 551
                .   :..  : ::  :   ::  .      :    . : : ::: :  *  :: :*  **

CeSUCH-1        GTYECGDVR---GSMRIHMQMATIHTAYGQFETAEIQIQEAGKVAVN----AHFLDANLL 676
DmIDA           DKLAVQDNIPFLLRMRVQVLLGYCGMANGRFSSETTMLLLRVSEEMS-EAQMDYELALVD 648
HsANAPC5        QKLLVHCQKLKNTEMVISVLLSVAELYWRSSSPTIALPMLLQALALSKEYRLQYLASETV 611
                 .            * : : :.         ..            :.     .:  :

CeSUCH-1        VVRRVGSLMLGRFMAREAYQVLHALSAKIEHFGSFIEKAIYHVSMARCLRLMHKD----P 732
DmIDA           LLLALQLLLLG--MPQKAYQAIKRCMHDIHINGGLYERAKTDFVFVRCLLAVNQNDAEKR 706
HsANAPC5        LNLAFAQLILG--IPEQALSLLHMAIEPILADGAILDKGRAMFLVAKCQVASAASYDQPK 669
                :   .  *:**  :..:* . ::     *   *.: ::.   . ..:*      .

CeSUCH-1        RVHLKQCKAQIIGNKWPAMEKL-----LLTELTILHHSGGLYPDEQKEMKAKERFGKIEA 787
DmIDA           KAQLLKSQVI-LQRAAQSFKKLSAHAKVLDVYVFLAQRFNEFGDRNLRNKYAGEFRRYFT 765
HsANAPC5        KAEALEAAIENLNEAKNYFAKVDCKERIRDVVYFQARLYHTLGKTQERNRCAMLFRQLHQ 729
                :..  :.    : .    : *:     :     :  :      . : . :    * :

CeSUCH-1        DFPGPCTWMFI-- 798
DmIDA           DHPIPREYLGSP- 777
HsANAPC5        ELPSHGVPLINHL 742
                : *     :
```

EMB-30

```
                                                        ax2003 splicing∨
CeEMB-30        MDFSIVKTQILNNRRTFRTPFKVSSMCFSSQNDLIALGSKTGEILLKRTSWKMIWKTNIN 60
DmAPC-4         ----MAQTSSMKLLGARNMSCIVERMEWNNKMDLIAYGTEKGEVVIQRLN--------WQ 48
HsANAPC4        MLRFPTCFPSFRVVGEKQLPQEIIFLVWSPKRDLIALANTAGEVLLHRLAS-------FH 53
                     .    :.     . .  :  : :. : **** ..  **::::*           :
```

```
CeEMB-30        MIQAVGTECKLDSSVSALHFSPDGRFLAAATSK-GIIHLLDVETGKVRFSVKAASEKIAK 119
DmAPC-4         KIVTFPTPGED-VRVRSLSWQMDETLLAVGYSN-GKVALLDAESGTI-ISGLIYEDDIKK 105
HsANAPC4        RVWSFPPNENTGKEVTCLAWRPDGKLLAFALADTKKIVLCDVEKPES-LHSFSVEAPVSC 112
                 : :. .  :    * .* :  *  :** . :.   : * *.*.    :     .  :

CeEMB-30        LHWNCVREKPFISNLGEFTTRIKNVEAIEGAIELAETTPNISQEEIAFVYQRLDEDGSSF 179
DmAPC-4         VYFSKAIN---------------SQENLGTYTCNVKDKHRRFLPKLQPMTN-IDPCLKTL 149
HsANAPC4        MHWMEVTV---------------ESSVLTSFYN-AEDESNLLLPKLPTLPKNYSNTSKIF 156
                :::  .                 . . :      .:   .    ::  : :  .   . :

CeEMB-30        KHEDAHKESLERTLISTETFRESLQNTILLATDDMDSKIIVLVAGVFPYMEIDISDTLLQ 239
DmAPC-4         DQKS-----------------FPKGSPCFLVVIMRSGKVHLLLLGALQAGSIDLTQHILH 192
HsANAPC4        SEENSDEI-------------IKLLGDVRLNILVLGGSSGFIELYAYGMFKIARVTGIAG 203
                ..:.                    .   *     ... .:   .    .*     :

CeEMB-30        YNQSLLMLYDMHYSSAFGGVSFLATTYGPFLDCKQNELKPPGAEPKKDGQGCHTLLFNVK 299
DmAPC-4         PHEFDVYDVRMNGDCNAIYALLRDGQELILLHFQNQVLQDCMAPMLELATHCAHILETKN 252
HsANAPC4        TCLALCLSSDLKSLSVVTEVSTNGASEVSYFQLETNLLYSFLPEVTRMARKFTHISALLQ 263
                          ::  .    .          :. : : *    .   . .     :    :

CeEMB-30        LNINSSLWDTALRYIRLLFGFNLYSISLETTKRNWEEQIDNLHSLFDTKTKAVKIGNVLL 359
DmAPC-4         Y-INDTQQCLTEAWETVQLEMDN-----------------KLTRYANSQ-PYGVISAHFL 293
HsANAPC4        Y-INLSLTCMCEAWEEILMQMDS-----------------RLTKFVQEKNTTTSVQDEFM 305
                  ** :       :  : : ::                 .*    : :     :   ::

CeEMB-30        EMLLSGSTDAAGEAFLERGLGTDGLDKIELFATKHMPEVCRIARGQLSTSARNLCFQRCE 419
DmAPC-4         ELHVFGFATFEVEEFLFETLSEKGFKKIANSVDLSLSNLQSLVFKQLNGAAINMFYFLNT 353
HsANAPC4        HLLLWGKASAELQTLLMNQLTVKGLKKLGQSIESSYSSIQKLVISHLQSGSESLLYHLSE 365
                .: : * :    : :* . *  .*:.*:         ..:  :.  :*. .: .: :

CeEMB-30        FSTSLSRYAKFIQLKDDDSFLYDEDPPAYLSESNIWLNTLEEKINILDMKTRHLGIQCLT 479
DmAPC-4         IAGFGRMSHFFESL------ISPDVANEAMRACGGFVLKVHELQRTIDTLAYDMKLFHAW 407
HsANAPC4        LKGMASWKQKYEPLG-----LDAAGIEEAITAVGSFILKANELLQVIDSSMKNFKAFFRW 420
                :         :  *       :         :   . :: . .*  . :*    .:

CeEMB-30        MMQELGHLVKWISMTKPFAKTMKVNALMKIKRMNIAKILLYIVRNFIPDPEAVKDIENRL 539
DmAPC-4         MIFTILRLSHQE-----IPDDLVLTEEENIAMADFICAMEPELDDRSDDEDELDSQSQTP 462
HsANAPC4        LYVAMLRMT----------EDHVLPELNKMTQKDITFVAEFLTEHFNEAPDLYNRKG--- 467
                :   : ::            .   :    ::   ::          .      :  .

CeEMB-30        FNLKELVSKQKKKDLEDKFDEVEYLYRLERIAEVERKKLIETFHEKFRFADLDDDDLTPL 599
DmAPC-4         PPARSKFNLERVG---------QYLDNAYLTQLYPRDP-VQLWEEMVTDNECLSN--CKL 510
HsANAPC4        ----KYFNVERVG---------QYLKDEDDDLVSPPNTEGNQWYDFLQNSSHLKE--SPL 512
                    . .. ::           :**           .   : : : .   .  .:    *

CeEMB-30        LFQELDHPIDIFTDSMMEQNDSSPFLEEDEGEPEQEEQKPDEEPEPEGEPQPACDLDRVG 659
DmAPC-4         FVPHDVNLSLVQQRDKMFN------------AIDAVFHKPTESISGSFKVSSTVICNDLP 558
HsANAPC4        LFPYYPRKSLHFVKRRMEN------------IIDQCLQKPADVIG---KSMNQAICIPLY 557
                :.    .         * :               :   :** :       :          :

CeEMB-30        SFFEKELSQKALEVLPKCDETFDCVLNERGSRMNSIEIQKMGILRVLEDLAATLSSPQKI 719
DmAPC-4         PMEPGEDHRDRDLVTCTYYVNEASRTDMLACTISGQ---EAMILEFSRAGDECVRCT-RI 614
HsANAPC4        RDTRSEDSTRRLFKFPFLWNNKTSNLHYLLFTILEDSLYKMCILRRHTDISQSVSNG-LI 616
                     *             .  .  .     :      :  **.         :     *

CeEMB-30        HCDKAGNQKKLEVSFIYEITSTPTHQGYQDAKISSVTPSYYKRHPFNQLSGMGRSIQVSV 779
DmAPC-4         TLEPGLFTTSVSEDF--CYLRFVDLQFYNESSLSILAQSINAGPGMRPHS---FFIQFSL 669
HsANAPC4        AIKFGSFTYATTEKVRRSIYSCLDAQFYDDETVTVVLKDTVGREGRDRLL---VQLPLSL 673
                  . .        ..          * *:: .:: :  .                : .*:

CeEMB-30        LQKNEFSSIVIVPTSDVLPEDENEVNDHVEKLKRCYEFEKIDVTVDKSSAENRPPGETDA 839
DmAPC-4         TAALNYSTQHRMG--PLVKLSEATVSQSIHDIADGAAFKGLDGFSDMLAVS--------- 718
HsANAPC4        VYNSEDSAEYQFTGTYSTRLDEQCSAIPTRTMHFEKHWRLLESMKAQYVAGN-------- 725
                    : *:   .        .*        . :     :. ::        .
```

```
CeEMB-30        MEVDSNELRIDVDLGPVVPEYDTLIELSQVHPLHHGELVLLGKFTSAQEGTGVMSQMMRS 899
DmAPC-4         ---------------------------------------------GSRKVATVLSDRKRK 733
HsANAPC4        ---------------------------------------------GFRKVSCVLSSNLRH 740
                                                             . :: : *:*.  *

CeEMB-30        ISSYPHEIPEDKDVFAKNSSEDGSSEIPIETLIIHPTIQLAAYLHDDGSKITIGDMKPEV 959
DmAPC-4         MTIFEMEIEEE----EDDTEMSQASFLDIS-------------------------KESVL 764
HsANAPC4        VRVFEMDIDDEWELDESSDEEEEASNKPVK-------------------------IKEEV 775
                :  :  :* ::     .. . . :*   :.                          :  :

CeEMB-30        PPIADYEEKTVRKTFRDYQRRRERYREDMGVMNMNDVQYDVLVQEAMDSGRDLTDNGESD 1019
DmAPC-4         AGVPDAEKQEA------------------------------------------------- 775
HsANAPC4        LSESEAENQQAGAAALAPEIVIKVEKLDPELDS--------------------------- 808
                   .: *:: .
```

MAT-2

```
CeMAT-2         ----------------------------------------------MRKYVLFFISG--- 11
DmSHTD-PA       QLKAAVHPDDDHLTAICVMDQDALRVYCSNGEDFLANLDFPVSQLWQTKYGLLLEKDSSN 240
HsANAPC1        DKSEKAYSSNEVEKCICILQSSCINMHSIEGKDYIASLPFQVANVWPTKYGLLFERSASS 195
                                                                ** *::  .

CeMAT-2         ---------------------------------------------NNDSQIWATTPNTPR 26
DmSHTD-PA       ALISHMS--IPMPRLFSMSHPLHEACPVVLKT----ATGSTGYMTEPEYTVVFTTEESDL 294
HsANAPC1        HEVPPGSPREPLPTMFSMLHPLDEITPLVCKSGSLFGSSRVQYVVDHAMKIVFLNTDPSI 255
                                                             :    :   . :.

CeMAT-2         VIARGGLERNIHTRTLARMVNEDAPGTSTPAAQS--RLQTTASPFHRTHLTQMCRRGDTN 84
DmSHTD-PA       VMLYDAKFFKHFVARLRKVTPEEINYVSQQMELG--QTLMGPRSMAGNSFSSTKQTGATP 352
HsANAPC1        VMTYDAVQNVHSVWTLRRVKSEEENVVLKFSEQGGTPQNVATSSSLTAHLRSLSKGDSPV 315
                *:  ..      .  * ::  *:   .      .       . .     : .  : . .

CeMAT-2         ASLLRDFTRMIRDTPRN-----FSKNTQHGNDRDFGDLERDPD----------------- 122
DmSHTD-PA       KATNLSFAARNINTTTTGMGNQFGLSQSQSFSGVLGQSNRASLGTPLSQLQSSISQQSMS 412
HsANAPC1        TSPFQNYSSIHSQSRSTSSPSLHSRSPSISNMAALSRAHSPALGVHSFSGVQRFNISSHN 375
                 :  .::      ::  .     .. . . .    :.  .  .

CeMAT-2         ------------------------VDLLLSKVCLECVYVEPKEGAIPKANKIFISNFLSD 158
DmSHTD-PA       VKDMRKLTHVKP------------AKPIEPELCMEHIWTENIYGTQREFCEMATRAFIHT 460
HsANAPC1        QSPKRHSISHSPNSNSNGSFLAPETEPIVPELCIDHLWTETIT-NIREKNSQASKVFITS 434
                                        .. : .::*:: ::.*       :  .     *:

CeMAT-2         MYINLVSVTGEVMKIIPIWKNAETTRKNLLEKGKHEPCVVDCVDAAFVMKSGITVVLGSD 218
DmSHTD-PA       DLVGQTFLCYLLARSCRLQLVRLTGYGRGEVQLSTHASTLAAKDAVGLKRMHMIAVLDPS 520
HsANAPC1        DLCGQKFLCFLVESQLQLRCVKFQESNDKTQLIFGSVTNIPAKDAAPVEKIDTMLVLEGS 494
                   .   :   :     :                     : . **. : :     **  .

CeMAT-2         FTTAMFGGNERIAPIFIK------------------------------------EMSNQRV 243
DmSHTD-PA       GSLLLYTGTVLISKVHITPFLAPTSIPTPLVTPMTAAPSPSASPAPSHVKTPMAAAGPAS 580
HsANAPC1        GNLVLYTGVVRVGKVFIP------GLPAPSLTMSNTMPRPSTPLDGVSTPKPLSKLLGSL 548
                 .  :: *   :. :.*

CeMAT-2         GRKFRLFSFAENR----------------------------------------------- 256
DmSHTD-PA       GIPSGSSSFVEVRRSSLLPTKAPGDVAAFEEELHMLSPIQPQPVSYTQRQAHNVCKSLRD 640
HsANAPC1        DEVVLLSPVPELRDSSKLHDSLYNEDCTFQQLGTYIHSIR-------------------D 589
                .      .. * *

CeMAT-2         ------IFAVNEMRCIVVEIPETVTCKSATELMRTCFLHLDRDLSRKLLIKWRSVKRDVD 310
DmSHTD-PA       PAGNRLTLVYATGRMLRIALPFLNDTRLLTRCVATLRQVLSPTQFLDFVIRWYSDRNPPG 700
HsANAPC1        PVHNRVTLELSNGSMVRITIPEIATSELVQTCLQAIKFILPKEIAVQMLVKWYNVHSAPG 649
                       :       : : :*     .     : :    *      .::::* . :   .

CeMAT-2         TERLDLDRKEMIDVAIFMLDN--------------VGVRVTNVVAQERADSPEGHGGKQM 356
DmSHTD-PA       SRNYSIEQEWLLFRSTLLALMGLTAAPDVDAGENYARCATPPLHTQFGGGATATESSDGS 760
HsANAPC1        GP--SYHSEWNLFVTCLMNMMGYNTDRLAWTRNFDFEGSLSPVIAPKKARPSETGSDDDW 707
                    . . :  :  : ::                      . : :   . ..   ...
```

```
CeMAT-2         RPRMSDSEVLSMMR----------QFFEEMTFRPKSEVITEDGYKCHLSVELDPNGEGFV 406
DmSHTD-PA       SCSSNSTLGGQDEPKKRRIYNDCDDFTDDDWEFLLLQTTLAPCGADGHSYSVNIGALLFR 820
HsANAPC1        EYLLNSDYHQNVES----------HLLNRSLCLSPSEASQMKDEDFSQNLSLDSSTLLFT 757
                    ..    .             .: :        :.          . .:: .   *

CeMAT-2         HTQDLLHAFHSQCEDWSINTMMHSILLELIPYAYLLAKVMNYRAFEEYYVQLFKHLLSQI 466
DmSHTD-PA       MIPAIFFSLHLLYEDLKLDADFYGALPYLATFLHQLAIDMQLESYVLHYILDFPELSNRT 880
HsANAPC1        HIPAIFFVLHLVYEELKLNTLMGEGICSLVELLVQLARDLKLGPYVDHYYRDYPTLVRTT 817
                    ::. :*   *: .::: :   :  *      **  ::  .:  :*   :  *

CeMAT-2         AIEFKIPPEIHEKFVGAIHIPKPCWSLNNVIAHIICERTTPETMESIPKFISKSSVRLLT 526
DmSHTD-PA       GKLSLLGAEHGAMMLHQELLRVPAPSVFAQLEHIIVGEEEVMPYTFLECVNERSRILLQL 940
HsANAPC1        GQVCTIDPGQTGFMHHPSFFTSEPPSIYQWVSSCLKGEG-MPPYPYLPGICERSRLVVLS 876
                .    : .     :     :     *:   :   :  .     .   :  . .:* : :

CeMAT-2         ILAVGRKFIGMG---------------------------TNIDMDCERWLGKDWKRRIGL 559
DmSHTD-PA       VSLVTHGHERLN------YWWQLLEIPGAVQAN--FTRRSKRNITADAPRSHQMLQLLLA 992
HsANAPC1        IALYILGDESLVSDESSQYLTRITIAPQKLQVEQEENRFSFRHSTSVSSLAERLVVWMTN 936
                :         :                                      :  .  .    ..     :

CeMAT-2         SGDILKSFRRIMNGKSSNSAGRASQLIELFEIGSITIDFMVLAVKVLMLKFQTDAFAGAQ 619
DmSHTD-PA       MRLTRRDIERFPAAVHLIVAEALEEARLSPPMGCSMATYELILRPELAAHAQLPFLETST 1052
HsANAPC1        VGFTLRDLETLPFGIALPIRDAIYHCREQPASDWPEAVCLLIGRQDLSKQACEGNLPKGK 996
                     :.:. :  .          .       .       ::     *  :      :  .

CeMAT-2         -----------SIEPKKCIYATADEMIS---------------IAHLRWKNDIRMHNVQL 653
DmSHTD-PA       GQPHCGRVYKEDSLSARCPPTGGSETDSPAQLRRDDMDNMDTKLLRLRFPDDMRVDEVRR 1112
HsANAPC1        -----------SVLSSDVPSGTETEEED------DGMNDMNHEVMSLIWSEDLRVQDVRR 1039
                           .  .         *  .                  :  * : :*:*:.:*:

CeMAT-2         MLNSSRPILIATNILRKNEDDNMKELQDRFLTQTSYRTFSQPFGRAFLDFRTAVPSLLTS 713
DmSHTD-PA       LLNSSEPVVIEVQQAPGTSDHEFIEEQEKQLFALCSRTMTLPVGRGMFTLRTMLPRPSES 1172
HsANAPC1        LLQSAHPVRVNVVQYPELSDHEFIEEKENRLLQLCQRTMALPVGRGMFTLFSYHPVPTEP 1099
                :*:*:.*: : .       .*.:: * ::. *    . **:: *.**.:: : :  *    .

CeMAT-2         IYIPRLNVGGMIYPSRVTCDPP--TTEIFKLCTEWGNFYNSLASALRIGSSETVRIDNEW 771
DmSHTD-PA       LTMPKLCLLGKEPLKGTTIEMQ--QIEFPANMQMWPSFHNGVATGLKISPQ-AQDIDSNW 1229
HsANAPC1        LPIPKLNLTGRAPPRNTTVDLNSGNIDVPPNMTSWASFHNGVAAGLKIAP--ASQIDSAW 1157
                : :*:* : *       .* :       :.       * .*:*.:*:.*:*..  :  **. *

CeMAT-2         IVMVSKN--IKSTAVIGGMTLGFGLNGHLAPFNMYHAHQMLSTFDKFHSVALLIGLSASN 829
DmSHTD-PA       IVYNKPKTHSHNALEHAGFLMALGLNGHLKTLSFMSVYKYLVKCDEMTNVGLLLGISAAH 1289
HsANAPC1        IVYNKPK-HAELANEYAGFLMALGLNGHLTKLATLNIHDYLTKGHEMTSIGLLLGVSAAK 1216
                **   . :    . :    .*: :.:******  :      :. * . .:: .:.**:*:**::

CeMAT-2         FTTCDVQIHKILATYLSFLMGPTPLEIKLDFTIQTAAISGLGLLFADSGNMTIAKKLVNE 889
DmSHTD-PA       RGTMDTKTTKLLSVHLEALLPATAMELDIPQSTQVAAIMGVGLLYQGSAKRHIAEVLLQE 1349
HsANAPC1        LGTMDMSITRLLSIHIPALLPPTSTELDVPHNVQVAAVVGIGLVYQGTAHRHTAEVLLAE 1276
                  * * .  ::*: ::  *: .*. *:.:  . *.**: *:**:: .:.:    *: *: *

CeMAT-2         IGRAPNRDEEPVTDRNAYKLSAGFSLGLIMLGKGNGSASTVIPFKQNIPPMSQRLIYMMN 949
DmSHTD-PA       IGRPPGPEMENSIERESYAMTAGLSLGLVTLGQGESPAG-----LRDLQLPDTLHYYMVG 1404
HsANAPC1        IGRPPGPEMEYCTDRESYSLAAGLALGMVCLGHGSNLIG-----MSDLNVPEQLYQYMVG 1331
                ***.*. : *    :*::* ::**::**:: **:*..  .        ::    .     **:.

CeMAT-2         GMRRDKCVFLPQVAPPVVNDVPNLPFSNGGMMTSSQVANHVKESEYINIHQSAEPAAIAL 1009
DmSHTD-PA       GVKR--------------------PIGGSQKEKYRLASFQVREGDTVNIDVTAPGATLAL 1444
HsANAPC1        GHRR--------------------FQTGMHREKHKSPSYQIKEGDTINVDVTCPGATLAL 1371
                * :*                     .    .    : :::*.: :*:. :.  *::**

CeMAT-2         GMMFMKMNNEFIANALALPGTITELERLKPDSMYSRVLAQCLVMWDSIEPTHDFVKSLIP 1069
DmSHTD-PA       GLMFFNSGNAAIAEWMQPPDSRYLLDMVRPDFLLLRTISRGLILWQDVRPDNAWFQAQFP 1504
HsANAPC1        AMIYLKTNNRSIADWLRAPDTMYLLDFVKPEFLLLRTLARCLILWDDILPNSKWVDSNVP 1431
                .:::: .*  **: :  *.:   *: ::*: :  *.::: *::*.: *    :..: .*
CeMAT-2         PVIREYATAALHFGVPIRRDEDGEEVHEAINDAEEKYWAEIVDKGTVSQTFLYAVSAACM 1129
DmSHTD-PA       RALRAHLKLPFYENEYAPEDYD-------------------VDYEAISQAYCNIMAGAAF 1545
HsANAPC1        QIIRENSIS--LSEIELPCSED-------------------LNLETLSQAHVYIIAGACL 1470
                  :*                . *                    ::  ::**:.   ::.*.:
```

```
CeMAT-2         AIALKFSSCGGPNEKNIVNTAFRIIEYYTKIVMPDGKSNKDMGSIRMCIYSGAYTRTSCL 1189
DmSHTD-PA       CIGLKYAGTEN-------LVAFATLRSVIKDFLR-------FPSRPMGECAGRTTVESCL 1591
HsANAPC1        SLGFRFAGSEN-------LSAFNCLHKFAKDFMT-------YLSAPNASVTGPHNLETCL 1516
                .:.::::.  .         **  :.   * .:          *      :*  .  :**
                               ax2012 V1208M
CeMAT-2         SMLITAMAILRVGTGDLEVMRYARLLRLCDKPESDWIATGKKHFEQMVAHQALGILMLGE 1249
DmSHTD-PA       MVLLISISLVFAGSGNCEILRIIRFLRSRVGPQYPHITYG----SHMAIHMSLGLLFLGA 1647
HsANAPC1        SVVLLSLAMVMAGSGNLKVLQLCRFLHMKTG---GEMNYG----FHLAHHMALGLLFLGG 1569
                 ::: ::::: .*:*: ::::  *:*:          :  *     ::. * :**:*:**

CeMAT-2         GRYAFKKDDLSIALTIISTFPTIPQSVSDNSHYHQPLRFLWSMAVEPRLLVPFDIAESCV 1309
DmSHTD-PA       GRFTISQTPESIAALVCAFFPKFPIHSNDNRYHLQALRHLYVLAVEPRLFLPRDIDTNKL 1707
HsANAPC1        GRYSLSTSNSSIAALLCALYPHFPAHSTDNRYHLQALRHLYVLAAEPRLLVPVDVDTNTP 1629
                **:::.    ***  : : :* :*   .** :: *.**.*: :*.****::* *:  .

CeMAT-2         VEVDVTIVMKPKDGNEPIVYKEKAPYLLPPLEDLQSISIGGGNYQLVHISLQ---SEDQV 1366
DmSHTD-PA       CLANISVLEVG---ATELRRLPIAPCILPVLSTLQQVVVDDENYWPVCFERSRNWDQLEK 1764
HsANAPC1        CYALLEVTYKGTQWYEQTKEELMAPTLLPELHLLKQIKVKGPRYWELLIDLSKGTQHLKS 1689
                  . : :                ** :** *  *:.: : . .*  : :. .   .. :

CeMAT-2         KVMKDIMTIGQGRVMLKRYGVDSSEMKIKEATTLYDDTPSLMSMFNNEDTAVELDEYEIQ 1426
DmSHTD-PA       ALEMSAPIDIKKRTGCLSHLEDPDRLKSMLAQTLTMEQSICWQIDMNDLQQFASERMVKQ 1824
HsANAPC1        ILSKDGVLYVKLRAGQLSYKEDPMGWQSLLAQTVANRNSEARAFKPETISAFTSDPALLS 1749
                 :  .     : *.    :  *.   :   * *:    .     :  :    .  :    .

CeMAT-2         CMMEKIDEGINLNSSDEYPNVQIELSCVRDVTERTTMDLAQLQKRSLKLLSES------- 1479
DmSHTD-PA       FLSRCLDTKGTDLSPPELMKRHQVMLLFYNAVVKDRMHFLPVYLTLYDHVTKS----MPN 1880
HsANAPC1        FAEYFCKPTVNMGQKQEILD--LFSSVLYECVTQETPEMLPAYIAMDQAIRRLGRREMSE 1807
                      .   .  .  *  .       . : . :   .:        . : .

CeMAT-2         -LDLWQDEVN----------------------------------VSNTINGLADAVQDMQI 1505
DmSHTD-PA       NIDVWQMKLIDAYLSRSQESE----HPLISVELIQMMQELFKQEMEDSTRELCLPLREFLS 1937
HsANAPC1        TSELWQIKLVLEFFSSRSHQERLQNHPKRGLFMNSEFLPVVKCTIDNTLDQWLQVGGDMCV 1868
                  ::** ::                                    :.::         ::
```

MUS-101

```
CeMUS-101         MEAPPAPKKARRSEVSRMQDESVLCDDDEEDSPFTLYFVDLPNEPTVKEIRNLEELFKTA 60
DmMUS-101-PA      -----------------------MSRNDQE--PFLVKFLKSSDN--SECFFKALESIKEL 33
HsTOPBP1          -----------------------MSRNDKE--PFFVKFLKSSDN--SKCFFKALESIKEF 33
                                         :. :*:*  ** : *:. .::   : : :  * :*

CeMUS-101         KAVGIMPEWIDSDALEDLQKSEDFFVLPCFRGKLFRKLQARKLKVYGPPIVLESIEDGKQ 120
DmMUS-101-PA      QSEDYLQIITDEEALKIRENDKSLYICDRFSGTVFDHLKQLGCRIVGPQVVTFCMRHQQC 93
HsTOPBP1          QSEEYLQIITEEEALKIKENDRSLYICDPFSGVVFDHLKKLGCRIVGPQVVIFCMHHQRC 93
                  ::   :    :.:**:  ::...:::   * * :* :*:    :: ** :*  .:.. :

CeMUS-101         LPQWNHPVYSSVFQDVKISFTGLNLTKKQELYEKIGWMCGVVGDALYHETTHLVTEKAEQ 180
DmMUS-101-PA      VPRAEHPVYNMIMSDVTVSCTSLDKDKREEVHKYVQMMGGRVYRDLNVSVTHLIAGEVGS 153
HsTOPBP1          VPRAEHPVYNMVMSDVTISCTSLEKEKREEVHKYVQMMGGRVYRDLNVSVTHLIAGEVGS 153
                  :*: :****. ::.**.:* *.*:  *::*::: :  * * *   *  ..***:: :. .

CeMUS-101         TEKYKAAVNNSIKLMRIGWIDDLWETSQTTMGRFSALSRDSVNSYALRVFEGLEMAITSI 240
DmMUS-101-PA      -KKYLVAANLKKPILLPSWIKTLWEKSQEKK--ITKYTDVNMEDFKCPIFLGCIICVTGL 210
HsTOPBP1          -KKYLVAANLKKPILLPSWIKTLWEKSQEKK--ITRYTDINMEDFKCPIFLGCIICVTGL 210
                   :** .*.* .  ::  .**. ***.** .    ::  :  .::.:   :* *  :.:*.:
                                                   ax2011 S273N
CeMUS-101         DGADRTNFIQLIEDHGGKVPGTMSKTRCSYLISDKITGVKYAKAVEWKSMQIVQSRWIRK 300
DmMUS-101-PA      NGIHRKTVQQLTAKHGGQYMGQLKMNECTHLIVQEPKGQKYECARRWN-VHCVTLQWFHD 269
HsTOPBP1          CGLDRKEVQQLTVKHGGQYMGQLKMNECTHLIVQEPKGQKYECAKRWN-VHCVTTQWFFD 269
                   * .*. . **  .***:  * :. ..*::** :: .* **  * .*: :: *  :*: .


CeMUS-101         CVDLGHLVDAGKYHPKYLTADHIR--SSTPKRDAN--VTESVPDISSIAGHGG-RLGTSS 355
DmMUS-101-PA      SIEKGFCQDESIYKAETRVEAKMVPDTSTPTAQSN-AESHTLADVSHISNINGSCVNETM 328
HsTOPBP1          SIEKGFCQDESIYKTEPRPEAKTMPNSSTPTSQINTIDSRTLSDVSNISNINASCVSESI 329
                  .:: *.  * . *:.:     :    :***. : *   :.::.*:* *:. ..  :. :
```

```
CeMUS-101        FNSSIPQMDQSYQHQSGSSFISFASTSKIPSSTFTNNDSTLGR----------SGSGVR- 404
DmMUS-101-PA     FGSTTSKLECSLENLENLDISMFQAPEDLLDGCRIYLCGFSGRKLDKLRRLINSGGGVRF 388
HsTOPBP1         CNSLNSKLEPTLENLENLDVSAFQAPEDLLDGCRIYLCGFSGRKLDKLRRLINSGGGVRF 389
                  .*  .::: : :: .. ..  * :...: ..       .  **          **.***

CeMUS-101        DGISTPIARVQT------------------------------------------------ 416
DmMUS-101-PA     NQLNEDVTHVIVGDYDDDVRQFWSKSSHRPHVVGAKWLLECFTKGYILPEESYIHTNYQP 448
HsTOPBP1         NQLNEDVTHVIVGDYDDELKQFWNKSAHRPHVVGAKWLLECFSKGYMLSEEPYIHANYQP 449
                 : :.  :::* .

CeMUS-101        --TPLIRYPTTQTASVQNIADVISDP---IDELRKNIDDGLGDLFEN------------- 458
DmMUS-101-PA     AGIAVSDQPGNQTAVLDK-SGSFSKSALVPAERLQQADEDLLAQYGNDDSTMVEAKLSEA 507
HsTOPBP1         VEIPVSHKPESKAALLKKKNSSFSKKDFAPSEKHEQADEDLLSQYENGSSTVVEAKTSEA 509
                    .:   * .::* :.:  . :*.      *  :: *:.*   : *

CeMUS-101        ------------------------------CMFYICGVDESRMEKWRRFLNETGATRVAK 488
DmMUS-101-PA     LEPEVGPCPGSAHREPCDDSTHISVQEENKSSVSHCILDDSTVREEGLFSQKSFLVLGFS 567
HsTOPBP1         R-----PFNDSTHAEPLNDSTHISLQEENQSSVSHCVPDVSTITEEGLFSQKSFLVLGFS 564
                                               . .  *  * * : :   * :::  .   .

CeMUS-101        FTSATNVVVVSPNQQERITIRKHLHQEDIAIVTVG---------------WVVECVKQRK 533
DmMUS-101-PA     VENKCNIVDIIREHAGKIVSLPSRIVADYAVVPLLGCEVDVTVGEVVTNTWLVTCIDNQT 627
HsTOPBP1         NENESNIANIIKENAGKIMSLLSRTVADYAVVPLLGCEVEATVGEVVTNTWLVTCIDYQT 624
                   .  *:. :  ::  :*         * *:*.:                *:* *:. :.

CeMUS-101        MISVEG------------------------------------------------------ 539
DmMUS-101-PA     LVDPKSNPLFTPVSVMSGVTPLEDCVISFSQCVGAERDSLVFLANHLGASVQEFFVRKAN 687
HsTOPBP1         LFDPKSNPLFTPVPVMTGMTPLEDCVISFSQCAGAEKESLTFLANLLGASVQEYFVRKSN 684
                 :.. :.

CeMUS-101        ----------------------------------YQWTENTADESQSSQSQLQPVRPQPLP 566
DmMUS-101-PA     AKKGMLASTHLIVKEPTGSKYEAAKKWSLPAVNISWLLETARIGKRADENHFLVDNAPKQ 747
HsTOPBP1         AKKGMFASTHLILKERGGSKYEAAKKWNLPAVTIAWLLETARTGKRADESHFLIENSTKE 744
                                                    *  :**  .: ::..   :   .

CeMUS-101        RTSSKSTIPYGKTSGP--------------SSTSTQVGIFSYHTYCVHCSVDQEVSDD-- 610
DmMUS-101-PA     EQVLETKIPNGVSSNPDLPAHPDAHLEIHRKKAVTPLDMNRFQSRAFRAVISQQRGQDPT 807
HsTOPBP1         ERSLETEITNGINLNSDTAEHPGTRLQTHRKTVVTPLDMNRFQSKAFRAVVSQHARQVAA 804
                 .   :: *. * . ..              ... * :.:  ::: ..:. :.*.  :

CeMUS-101        ---LKEKIPLNGGKLMDDDPD--YAEFVIFGHSGPMHELIS-------------------F 646
DmMUS-101-PA     FPPVRQPLTKEPSLHLDTPSKFLSKDKLFKPSFDVTDALAALETPNAASQ-KRKLSSPLS 866
HsTOPBP1         SPAVGQPLQKEPSLHLDTPSKFLSKDKLFKPSFDVKDALAALETPGRPSQQKRKPSTPLS 864
                    : : :  : .  :* *.   ::  :*  *  : : ::

CeMUS-101        DAVVTDFYIYASIGNNRFLNRSCYPLFVPLPRPPILIFNQRGFQLKCKDPFLRDR---VR 703
DmMUS-101-PA     EVIVRNLTVALANSSRNTDSHSASPQLKGAHLEEEETRKPLDSVVVCVSKKLSKKQSELN 926
HsTOPBP1         EVIVKNLQLALANSSRNAVALSASPQLKEAQSEKEEAPKPLHKVVVCVSKKLSKKQSELN 924
                 :.:* :: :  : ....    *. * :            :     : * .  * .:   :.

CeMUS-101        DIIEDNGGRIVEQLEP--KDFIIMIDAEDNPPRYHSR------TLDFSWIIASVSRCRLQ 755
DmMUS-101-PA     GVAASLGAEYRWSFDETVTHFIYQGRANDSNREYKSAKERGVHIVSEHWLLECAQEYKHL 986
HsTOPBP1         GIAASLGADYRWSFDETVTHFIYQGRPNDTNREYKSVKERGVHIVSEHWLLDCAQECKHL 984
                 .:  . *.    .::   ..**    .:*.  .*:*        :.  *:: .... :

CeMUS-101        PIDNFLYKNN-------TRPLSGFQRDDDLWEKCSREKNNSTQEMEVEVEDRHRVQDLPV 808
DmMUS-101-PA     PESLYPHTYNPKMSLDINTVQDGRLCNSRAPLAVSASKDDGPDHLSVEGNETNTMGTNDK 1046
HsTOPBP1         PESLYPHTYNPKMSLDISAVQDGRLCNSRLLSAVSSTKDDEPDPLILEENDVDNMATNNK 1044
                 * . : :. *        .   .*   :.      *  *:: .: : :* :: . :

CeMUS-101        ETGNGN-IGDMTTPYVNPYFPDLRKPYTMNLQLDGVSEYINNMESPARETQETLTTSRVG 867
DmMUS-101-PA     ESPLLNGSGRDDCKGALTQALEMRENFQKQLQEIMSATCIVKTPAQKTCMSRSSCNSASS 1106
HsTOPBP1         ESAPSNGSGKNDSKGVLTQTLEMRENFQKQLQEIMSATSIVKPQGQRTSLSRSGCNSASS 1104
                 *:   *  *       . .   ::*: :  :**     :  * :  .      ..:  .*  .
CeMUS-101        SILRKAVVNTGRN---EDFEDEPSTCHLIRPRVPENRKTVSSTPVLVRDVNRSMRYMPMD 924
DmMUS-101-PA     TPDSARSVRSGRSRVLEALRQSRQAVPDVNTEPSQNEQIIWDDPTAREERARLASNLQWP 1166
HsTOPBP1         TPDSTRSARSGRSRVLEALRQSRQTVPDVNTEPSQNEQIIWDDPTAREERARLASNLQWP 1164
                 :       ..:**.   * :.:. .:   :... .:*.: : . *.  .:  *    :
```

```
CeMUS-101        ESFADQNQEHEDLNR-------------------------------------------- 939
DmMUS-101-PA     SDPTQHSELQVEIKMPDDSPSRKPVYHSEIAEQASC------VTQAPGHPGSEEPEPPVA 1220
HsTOPBP1         SCPTQYSELQVDIQNLEDSPFQKPLHDSEIAKQAVCDPGNIRVTEAPKHPISEELETPIK 1224
                 .  :: .: : :::

CeMUS-101        ----------------------------------------RYAMNPRFLLSVSNMDPQRAADLQ 963
DmMUS-101-PA     ERPLIPEPQAPAVASPLAKPPVAPQPADKIETQEETHRKVKKQYVFQMSSLNSQERIDYC 1280
HsTOPBP1         DSHLIPTPQAPSIAFPLANPPVAPHPREKIITIEETHEELKKQYIFQLSSLNPQERIDYC 1284
                                                     :  :: ::::.:*.::.*.  *

CeMUS-101        ETIMKLGGTIEREFN--KDVTHLIASNMQRAPKVLCSIAAGKWCLTPDYVTKSAEVGRWL 1021
DmMUS-101-PA     RLIKDLGGSVIEKQCSDPSCTHMVVGYPLRNEKYLASMAAGKWVLHRSYLDACKTAGRFV 1340
HsTOPBP1         HLIEKLGGLVIEKQCFDPTCTHIVVGHPLRNEKYLASVAAGKWVLHRSYLEACRTAGHFV 1344
                 . * .*** : .:       **::..   *  * *.*:***** *  .*:  .  .*:::

CeMUS-101        DEKSFEWTREKLLACSKKNESLKERDSRKVSENLAAVCGLWRQRVAEMPVTFSVSMENRQ 1081
DmMUS-101-PA     QEEDYEWGSSSILDALPDVTEHQQK--------LALAAMRWRKRIQQS------QESGIV 1386
HsTOPBP1         QEEDYEWGSSSILDVLTGINVQQRR--------LALAAMRWRKKIQQR------QESGIV 1390
                 :*:.:**  ..:*         :.:         ** ..  **::: :       . ..

CeMUS-101        NGAFSDWRCVIHLDDKRAGVFSSILEAGGAIVHSISEYVEVSTLKPNIVLASKEFPWNPQ 1141
DmMUS-101-PA     EGAFSGWKAILRVDRPREAGFKRLLQAGGAKVLSG---HPEPLLKDATHLFCDFNKLKPD 1443
HsTOPBP1         EGAFSGWKVILHVDQSREAGFKRLLQSGGAKVLPG---HSVPLFKEATHLFSDLNKLKPD 1447
                 :****.*: ::::*  * . *. :*::*** * .        . :*    * ..    :*:

CeMUS-101        SAVLLKRDNIPIYVFDILYDFLIDRDNLNRTKFWHNVYMKA------------------- 1182
DmMUS-101-PA     DCRVFIAEATAQNMVCLKTEYIADYLMLESPPCADNYRVSEAALFH--NKKGGPGLPQKR 1501
HsTOPBP1         DSGVNIAEAAAQNVYCLRTEYIADYLMQESPPHVENYCLPEAISFIQNNKELGTGLSQKR 1507
                 .. :   :  .  :  :  ::: *    : .   .*  :
```

XPO-2

```
CeXPO-2        -------MEQIGAALQQTLEPDAAIRKRGEEALRSLQSNPGYIIQILQLVVNEQQQIAPQ 53
DmCAS-PA       MEVTEANLQLLAGYLQQTLSADPNVRRPAEKLLESTELQQNYPILLLNLIDKAQM--DMT 58
HsCSE1L        MELSDANLQTLTEYLKKTLDPDPAIRRPAEKFLESVEGNQNYPLLLLTLLEKSQ---DNV 57
                      :: :   *::**..*. :*: .*: *.* : : .* : :* *: : *

CeXPO-2        IRIAAAVALKNFVKRNWGPAPEV----EMGQEDEEQFRSMLLEAMFNTKSNVQEILSNAL 109
DmCAS-PA       TRVAGAIAFKNYIKRNWAAHLDSDGPDRIHESDRNTIKTLIVTLMLHSPVALQKQLSDAV 118
HsCSE1L        IKVCASVTFKNYIKRNWRIVEDE--PNKICEADRVAIKANIVHLMLSSPEQIQKQLSDAI 115
                ::..::::**::****    :     .: : *.  ::: ::  *: :   :*: **:*:

CeXPO-2        YLIAQRDFPEKWPDLVPYLSKFLNGADLNHLVASLASMEQIFRKFRFESKSAELWKELKK 169
DmCAS-PA       SIIGKYDFPKKWPQLIDEMVERFASGDFNVINGVLQTAHSLFKRYRYEFKSQALWEEIKF 178
HsCSE1L        SIIGREDFPQKWPDLLTEMVNRFQSGDFHVINGVLRTAHSLFKRYRHEFKSNELWTEIKL 175
                :*.: ***:***:*:  : : : ..*:: : . * : ..:*:::*.* **  ** *:*

CeXPO-2        CLLSTQEPLTLLLRNMMEVGQRKDQLGADEIAQWLRVLLLIAKVYHSLCSQEIPEYFEDH 229
DmCAS-PA       VLDRMAKPLTDLLQATMQLTKVHENN-AGALKVIYGSLVLVNKVFFSLNSQDLPEFFEDN 237
HsCSE1L        VLDAFALPLTNLFKATIELCSTHAND-ASALRILFSSLILISKLFYSLNFQDLPEFFEDN 234
                *     *** *::  ::: . : :  *. :      *:*: *::.**  *::**:***:

CeXPO-2        LKDWMPHFLHLVQIDAP-TQTSNSGEPTTLDELKHEICEIFTLYSQRYEEEISEFVPDII 288
DmCAS-PA       INTWMGAFIQQLAADVPSLRTADDEDAGVLEHLRAQVCENICLYAKKYDEEFKPFMEQFV 297
HsCSE1L        METWMNNFHTLLTLDNKLLQTDDEEEAGLLELLKSQICDNAALYAQKYDEEFQRYLPRFV 294
               :: **  *   :  *    :* :. :.  *: *: *: ::*:   **:::*:**:. ::  ::

CeXPO-2        LAVWNLLKSTGPDTRYDTMVCAALEFLSMVSQRQYYEGHFTGEGVLKTLAENVCVQNLLL 348
DmCAS-PA       TAVWELLVKTSLHTKYDSLVSHALQFLSVVADRQHYQSIFENPEILAQICDKVVIPNLDI 357
HsCSE1L        TAIWNLLVTTGQEVKYDLLVSNAIQFLASVCERPHYKNLFEDQNTLTSICEKVIVPNMEF 354
                *:*:** .*. ..:** :*. *::**: *: *.:* :*:. * .   *  :.::* : *: :

CeXPO-2        RQQDMELFEDEPLDYMKRDIEGTDVGTRRRGAIDLARGLCRRFEAQMLPCLGEIVQNLLG 408
DmCAS-PA       RPSDEEIFEDSPEEYIRRDIEGSDIDTRRRAACDLVKTLSINFEQKIFGIFGQYLERLLT 417
HsCSE1L        RAADEEAFEDNSEEYIRRDLEGSDIDTRRRAACDLVRGLCKFFEGPVTGIFSGYVNSMLQ 414
               *  * * ***.. :*::**:**:*:*:.****.* **.: *.  **  :    :.  :: :*
```

```
CeXPO-2         SG------DWIKIDIVYSLITAIAVKTETAKSGVTATNPLVDINDFFITQVATHLN-ADV 461
DmCAS-PA        KYKENPATNWRSKDTAIYLVTSWASRGGTQKHGITQTSELVPLPEFCAQQIIPELERPNI 477
HsCSE1L         EYAKNPSVNWKHKDAAIYLVTSLASKAQTQKHGITQANELVNLTEFFVNHILPDLKSANV 474
                .       :*   * .  *:*: * :  * * *:* :. ** : :*   :: ..*: .::

CeXPO-2         NQTPILKADALKFAVTFRKQLAPEHLMTAIKSADALLSSNTPILHKYAAYAIEKILLADS 521
DmCAS-PA        NEFPVLKAAAIKYVMVFRSILGPQVLASCLPQLIRHLPAESSVVHSYAACSVEKILSMRD 537
HsCSE1L         NEFPVLKADGIKYIMIFRNQVPKEHLLVSIPLLINHLQAESIVVHTYAAHALERLFTMRG 534
                *: *:*** .:*: : **. :  : *  .:       * ::: ::*.*** ::*:::   .

CeXPO-2         NK---IFSAQNLP--VSSILQNLVTAFDKDAKAQNSPYLIKAILRIIVILDDDTIRHADA 576
DmCAS-PA        ASNAIVFGPQILAPYTTELISGLFATLSLPGSGENE-YVMKAIMRSFSVLQSAAMPFMGV 596
HsCSE1L         PNNATLFTAAEIAPFVEILLTNLFKALTLPGSSENE-YIMKAIMRSFSLLQEAIIPYIPT 593
                 .   :* .  :.  .  :: .*. ::   ...:*. *::***:* : :*:.  : .  .

CeXPO-2         IAVKLAQLVESATKNPADSVHTHFLFETICVLITKTRTIG----ASLDAQLLPLIEVIFR 632
DmCAS-PA        ALPRLTEILTQVAKNPSRPQFNHYLFETLALCIKIVCHADSSAVSSFEEALFPVFQGILQ 656
HsCSE1L         LITQLTQKLLAVSKNPSKPHFNHYMFEAICLSIRITCKANPAAVVNFEEALFLVFTEILQ 653
                   :*:: :  .:***: . ..*::**::.: *   .   .      .::  *: ::  *::
                                                             ax2013 S679L
CeXPO-2         EDLEDLIPYALQITGVLVSSCIARNSSIDQFSPFLPFLLSERLWARSANVPAALSVLEVI 692
DmCAS-PA        QDIVEFMPYVFQMLSVLLEMREGTGTIPEPYWALFPCLLSPALWDRTGNVTPLIRLISAF 716
HsCSE1L         NDVQEFIPYVFQVMSLLLETHKN--DIPSSYMALFPHLLQPVLWERTGNIPALVRLLQAF 711
                :*: :::**.:*: .:*:.          . : .::* **.  ** *:.*:.. : ::..:

CeXPO-2         LSVNAQRVVS---ENSGLILSHLARLLGSKTLDQYGFQLAATILPSIEHFEG-SAMTFVL 748
DmCAS-PA        IKQGSAQIQ--ALGKLSGILGIFQKMIASKANDHEGFYLLQNLLSYYPPAEIQTNLRQIF 774
HsCSE1L         LERGSNTIASAAADKIPGLLGVFQKLIASKANDHQGFYLLNSIIEHMPPESVDQYRKQIF 771
                :. .:  :       :   :*. : :::.**: *: ** *  .::       .       ::

CeXPO-2         NTMFRRVQSSKTPKFMKLFIVFLCRFTIARSAQDLVQSCENIQTGMFGMLIEKVVCIDLP 808
DmCAS-PA        GLLFQRLSLSKTPKYLSGIIIFFSFYVIKFSGSQMAQLIDEIQPNLFGMLLDRVFITEMG 834
HsCSE1L         ILLFQRLQNSKTTKFIKSFLVFINLYCIKYGALALQEIFDGIQPKMFGMVLEKIIIPEIQ 831
                  :*:*:. ***.*::. :::*:  : *  ..  : :  : **. :***:::::.  ::

CeXPO-2         GLKQTTTGPEKRIIAIGMGNLLADVTQQLVGQYG-ILSYEVAMLLEAASASDRAVLSPEE 867
DmCAS-PA        ---KIPKEQDRKMVAVGVTKLLTETPEILQQQYATFWPRLLHSLIDLFERPPEKLMGLEI 891
HsCSE1L         ---KVSGNVEKKICAVGITKLLTECPPMMDTEYTKLWTPLLQSLIGLFELPEDDTIPDEE 888
                   : .   :::: *:*: :**:: .  :  :*  : .  :  *:    . .    :  *

CeXPO-2         EQASMYNAEG--EFVNPFCRLSYAPK--QPPVAANIANHKAYFAQAVLVRGPGNCPETLR 923
DmCAS-PA        GETAGVAEDPDAGYQVAFAQLTHAQPNQQDHLAEIKD-ARQFLATSLSKFAQARAGEFST 950
HsCSE1L         -HFIDIEDTP--GYQTAFSQLAFAGKKEHDPVGQMVNNPKIHLAQSLHKLSTACPGRVPS 945
                 .           :  .*.:*:.*    :  :.      : .:* ::   . .   .

CeXPO-2         SVPP----EIVTYLQSIQQ------- 938
DmCAS-PA        LLSP-LEPEYKQVLQKYCDQAGVRIA 975
HsCSE1L         MVSTSLNAEALQYLQGYLQAASVTLL 971
                 :..    *    **   :
```

Figure S4 Multiple alignment of suppressor genes**.** Protein sequences of suppressor genes were aligned with their *D. melanogaster* and human orthologs by ClustalW2 using Gonnet PAM 250 matrix. Amino acids mutated in *dd5* and suppressor alleles are highlighted. If amino acids are conserved, a symbol “*” (identical amino acid in all three species) or “:” (strong conservation that scores >0.5) or “.” (weak conservation that scores ≤0.5) is shown below the sequence.
