# Supplementary material for: Identification of Suppressors of mbk-2/DYRK by Whole-Genome Sequencing
Source: G3 (Bethesda). 2013 Dec 17;4(2):231–41. doi: 10.1534/g3.113.009126 (PMC3931558; doi:10.1534/g3.113.009126)
Supplement: Supporting Information [file supp_g3.113.009126_TableS1.pdf]

Table S1 Nematode strains used in this study

Strain	Genotype	Description
**TH48**	*mbk-2(dd5)*	
**EU1065**	*und-119(ed3; orIs1 III [unc-119; pie-1 promoter::GFP::MEI-1 fusion]*	
**CB4856**	Hawaiian (Hw)	
**JH2291**	*mbk-2(dd5);* Hw	N2 gaps on I (1.5-12.8Mb) and IV (0-15.8Mb)
**JH2996**	*mbk-2(dd5);cdc-37(ax2001)*	
**JH2997**	*mbk-2(dd5);plk-1(ax2002)*	
**JH2998**	*mbk-2(dd5);emb-30(ax2003)*	
**JH2999**	*mbk-2(dd5ax2004)*	
**JH3000**	*mbk-2(dd5ax2005)*	
**JH3001**	*mbk-2(dd5ax2006)*	
**JH3002**	*mbk-2(dd5ax2007)*	
**JH3003**	*mbk-2(dd5);plk-1(ax2008)*	
**JH3004**	*mbk-2(dd5);tat-4(ax2009)*	
**JH3005**	*mbk-2(dd5);such-1(ax2010)*	
**JH3006**	*mbk-2(dd5);mus-101(ax2011)*	
**JH3007**	*mbk-2(dd5);mat-1(ax2012)*	
**JH3008**	*mbk-2(dd5);xpo-2(ax2013)*	
**JH3009**	*mbk-2(dd5);fzy-1(ax2014)*	
**JH3010**	*mbk-2(dd5);unc-119(ed3); orIs1 III*	cross JH1570 with TH48
**JH3011**	*mbk-2(dd5);cdc-37(ax2001);unc-119(ed3); orIs1 III*	cross JH3010 with JH2996
**JH3012**	*mbk-2(dd5);plk-1(ax2002);unc-119(ed3); orIs1 III*	cross JH3010 with JH2997
**JH3013**	*mbk-2(dd5ax2004); unc-119(ed3); orIs1 III*	cross JH3010 with JH2999
**JH3014**	*mbk-2(dd5ax2005); unc-119(ed3); orIs1 III*	cross JH3010 with JH3000
**JH3015**	*mbk-2(dd5);plk-1(ax2008); unc-119(ed3); orIs1 III*	cross JH3010 with JH3003
**JH3075**	*mbk-2(dd5);tat-4(ax2009); unc-119(ed3); orIs1 III*	cross JH3010 with JH3004
**JH3076**	*mbk-2(dd5);such-1(ax2010); unc-119(ed3); orIs1 III*	cross JH3010 with JH3005
**JH3077**	*mbk-2(dd5);mus-101(ax2011); unc-119(ed3); orIs1 III*	cross JH3010 with JH3006
**JH3078**	*mbk-2(dd5);mat-1(ax2012); unc-119(ed3); orIs1 III*	cross JH3010 with JH3007
**JH3079**	*mbk-2(dd5);xpo-2(ax2013); unc-119(ed3); orIs1 III*	cross JH3010 with JH3008
**JH3080**	*mbk-2(dd5);fzy-1(ax2014); unc-119(ed3); orIs1 III*	cross JH3010 with JH3009
**JH3086**	*mbk-2(dd5);emb-30(ax2003); unc-119(ed3); orIs1 III*	cross JH3010 with JH2998
**JH3087**	*mbk-2(dd5);xpo-2(ax2013); unc-119(ed3); orIs1 III*	cross JH3010 with JH3008
**CB5584**	*mIs12 II [myo-2::GFP, pes-10::GFP, F22B7.9::GFP]*	strong GFP in 4-cell embryos, pharynx muscle and gut.
**JH1279**	*mIsXX IV [myo-2::GFP, pes-10::GFP, F22B7.9::GFP]*	PD4790
**JH3085**	*mbk-2(dd5);mIs12 II*	cross CB5584 with TH48
